# Supplementary material for: Controllable multiple-step configuration transformations in a thermal/photoinduced reaction
Source: Nat Commun. 2022 May 23;13:2847. doi: 10.1038/s41467-022-30597-w (PMC9126889; doi:10.1038/s41467-022-30597-w)
Supplement: Supplementary file 1 — Supplementary Information [file 41467_2022_30597_MOESM1_ESM.pdf]

Supplementary Information

Controllable multiple-step configuration transformations in a thermal/photoinduced reaction

Meng-Fan Wang[1], Yan Mi[2], Fei-Long Hu[2]*, Hajime Hirao[3]*, Zheng Niu[1]*, Pierre Braunstein[4],Jian-Ping Lang[1]*

[1]College of Chemistry, Chemical Engineering and Materials Science, Soochow University, Suzhou 215123, Jiangsu, People's Republic of China.

[2]Guangxi Key Laboratory of Chemistry and Engineering of Forest Products, Guangxi University for Nationalities, Nanning, 530006, P. R. China.

[3]School of Life and Health Sciences, The Chinese University of Hong Kong, Shenzhen, Longgang Dist., Shenzhen 518172, Guangdong, People's Republic of China.

[4]Université de Strasbourg - CNRS, Institut de Chimie (UMR7177 CNRS), 4 rue Blaise Pascal-CS 90032, 67081 Strasbourg, France

Correspondence and requests for materials should be addressed to F.L.H. (email: hflphd@163.com) or to H.H. (email: hirao@cuhk.edu.cn) orto Z.N. (email: zhengniu@suda.edu.cn) or to J.P.L. (email: jplang@suda.edu.cn)

Contents

1. General information 3

Powder X-raydiffraction analysis 3

Elemental analyses 3

Thermogravimetric analysis 3

Differential scanning calorimetry analysis 3

Fourier-transform infrared (IR) analysis 4

Mass spectra (MS) analysis 4

2. Synthetic procedures 4

3. X-ray data collection and structure determination 8

4. Solution NMR spectra of F-1,3-bpeb, digested CPs and isomeric cyclic compounds. 13

5. Solution NMR spectra of the transformation processes 33

6. Mass spectra of pure compounds 1, 1', 2α and 2β 35

7. Powder X-ray diffraction of CPs 37

8. In-situ powder X-ray diffractionof the transformation processes. 38

9. Thermogravimetric analysis and differential scanning calorimetry analysis .. 39

10. Fourier-transform infrared (FT-IR) spectra analysis 41

11. Computational methods and results. 42

11.1. Stability of **CP1** in the singlet ground and triplet excited states 43

11.2. Comparison of three possible pathways for the transformation process from **CP1** to **CP1-1** 44

11.3. Reaction pathway in the transformation processes from **CP1** to **CP1-1**, **CP1-1** to **CP1-2β**, **CP1-2β** to **CP1-1'** and **CP1-1'** to **CP1-2α**. 45

11.4. XYZ coordinates of optimized geometries 50

12. Supplementary references 75

1. General information

All reagents were obtained commercially and used without further purification. 1,3-dibromo-5-fluorobenzene (Adamas, 98%), 4-vinylpyridine (Aladdin, 96%, stabilized), K_2CO_3 (Aladdin, 99.99%), $(PPh_3)_2PdCl_2$ (Adamas, 98%), 3,5-dibromobenzoic acid (TCI, 97%), sodium hydroxide (Aladdin, 96%), $3CdSO_4 \cdot 8H_2O$ (Aladdin, 99.0%) and anhydrous Na_2SO_4 (Aladdin, 99.0%) were used as received. Deoxygenated N,N-dimethylformamide (Aladdin, 99.8% Anhydrous) was degassed with N_2 and dried on molecular sieves before use. Concentrated HNO_3 was purchased from Shanghaihushi at F.W. 63.01% purity. $CDCl_3$ (CIL, 99.8%), DMSO-d_6 (CIL, 99.9%) were used to collect nuclear magnetic resonance (NMR) spectra. All other solvents, dichloromethane, trichloromethane, and ethanol were purchased from Macklin at 99.8% purity and used without further purification. Nitrogen was purchased from Airgas at 99.9% purity.

Powder X-raydiffraction analysis

Powder X-raydiffraction (PXRD) patterns were acquired on a PANalytical X'Pert PRO MPDsystem (PW3040/60) using Cu Kα radiation (λ = 1.5406Å) from 5° to 50° with a scanning step size of 0.02^o.

Elemental analyses

Elemental analyses (C, H, N) were performed using a PE 2400 II elemental analyzer.

Thermogravimetric analysis

Thermogravimetric analyses (TGA) were performed on a Mettler Toledo Star System under a nitrogen atmosphere at a heating rate of 10 ^oC min^{-1}.

Differential scanning calorimetry analysis

DSC was performed on a PerkinElmer DSC8000 system under a nitrogen atmosphere at a heating rate of 30 ^{o}C min^{-1}. Samples ranging in size from 3.0 - 15.0 mg were placed in an aluminium pan.

Fourier-transform infrared (IR) analysis

IR analyses were recorded on a Bruker VERTEX 70+HYPERION 2000 spectrometer (4000 - 600 cm^{-1}) and a Varian 1000 spectrometer using KBr disks (4000 - 400 cm^{-1}).

Mass spectra (MS) analysis

The mass spectra were recorded at Bruker micrOTOF-Q IIImass spectrometer.

2. Synthetic procedures

Synthesis of 4,4'-(5-fluoro-1,3-phenylene)bis(ethene-2,1-diyl))dipyridine (F-1,3-bpeb). Ligand F-1,3-bpeb was prepared according to the literature methods[1,2]. The synthesis of F-1,3-bpeb ligand was based on the Heck reaction and using standard Schlenk techniques. To a 200 mL Schlenk tube involving 1,3-dibromo-5-fluorobenzene (2.52 g, 0.01 mol), 4-vinylpyridine (2.31 g, 0.022 mol), and K_2CO_3 (2.76 g, 0.02 mol) was added $(PPh_3)_2PdCl_2$ (0.084 g, 0.12 mmol). The tube was degassed under vacuum and then backfilled with N_2 for three times. Deoxygenated N,N-dimethylformamide (DMF, 50 mL) was added into the tube under N_2, the tube sealed, and the mixture heated to 120 °C with stirring for 2 days. After cooling to ambient temperature, the solid dark mass was dissolved in CH_2C1_2 (100 mL), extracted thoroughly with water (3 × 50 mL), and dried over anhydrous Na_2SO_4. The organic phase was concentrated under vacuum to give F-1,3-bpeb ligand as a light yellow powder. Yield: 2.55 g (84.4%). ^1H NMR (400 MHz, DMSO-d_6): δ 8.59 (d, J = 6.0 Hz, 4H), 7.79 (s, 1H), 7.59 (d, J = 16.0 Hz, 2H), 7.58 (d, J = 6.0 Hz, 4H), 7.52 (d, J = 10.0 Hz, 2H), 7.43 (d, J = 16.0 Hz, 2H); ^{13}C NMR (100 MHz, DMSO-d_6):

δ 164.07,161.66, 150.15, 143.78, 139.14, 131.50, 128.06, 122.28, 120.98, 113.29, 113.06; ^{19}F NMR (377 MHz, DMSO-d_6): δ -113.13 ppm; analysis (calcd., found for $C_{20}H_{15}FN_2$): C (79.45, 79.29), H (5.00, 5.10), N (9.27, 9.20).

Synthesis of compound 1: Pure product **1** were isolated from the sample of **CP1-1**. To a 100 mL flask was loaded a crystalline sample (400 mg) of **CP1-1**, 5 mL 4M NaOH solution. The mixture was stirred for 1 h at room temperature. Then CH_2Cl_2 (3 × 20 mL) was added, followed by removal of the aqueous layer by extraction, washing them with H_2O (10 mL) and dried with anhydrous Na_2SO_4. The combined organic extract was concentrated to dryness under vacuo to give a white powder. Yield 85.1% based on F-1,3-bpeb.

^{1}H NMR (400 MHz, $CDCl_3$): δ 8.56 (d, J = 6.0 Hz, 4H), 8.45 (d, J = 6.0 Hz, 4H), 7.30 (d, J = 6.0 Hz, 4H), 7.15 (d, J = 16.0 Hz, 2H), 7.03 (d, J = 6.0 Hz, 4H), 7.02 (s, 4H), 6.87 (d, J = 16.0 Hz, 2H), 6.82(s, 2H), 4.49 (s, 4H); ^{13}C NMR (100 MHz, $CDCl_3$): δ 164.44, 161.98, 150.25, 150.02, 147.91, 143.90, 141.90, 141.82, 138.60, 138.52, 131.62, 127.91, 122.97, 122.71, 120.93, 115.00, 114.78, 112.05, 111.83, 46.66, 46.30; ^{19}F NMR (377 MHz, $CDCl_3$): δ -115.53 ppm; HRMS(m/z): $[M]^+$ Calcd for $C_{40}H_{30}F_2N_4$, 605.2517; found: 605.2502; analysis (calcd., found for $C_{40}H_{30}F_2N_4$): C (79.45, 79.41), H (5.00, 5.02), N (9.27, 9.29).

Synthesis of compound 1': Pure product **1'** was isolated from the sample of **CP1-1'** and prepared using the same route as compound **1**. Yield 82.1% based on F-1,3-bpeb.

^{1}H NMR (400 MHz, $CDCl_3$): δ 8.56 (d, J = 6.0 Hz, 4H), 8.45 (d, J = 6.0 Hz, 4H), 7.29 (d, J = 6.0 Hz, 4H), 7.15 (d, J = 16.0 Hz, 2H), 7.03 (d, J = 6.0 Hz, 4H), 7.02 (s, 4H), 6.87 (d, J = 16.0 Hz, 2H), 6.82 (s, 2H), 4.49 (s, 4H); ^{13}C NMR (100 MHz, $CDCl_3$): δ

164.37, 161.93, 150.42, 150.08, 147.87, 143.75, 141.91, 141.84, 138.65, 138.57, 131.54, 127.99, 122.97, 122.72, 120.89, 114.97, 114.75, 112.02, 111.83, 46.67, 46.31; ^{19}F NMR (377 MHz, $CDCl_3$): δ -115.53 ppm; HRMS(m/z): $[M]^+$ Calcd for $C_{40}H_{30}F_2N_4$, 605.2517; found: 604.2508; analysis (calcd., found for $C_{40}H_{30}F_2N_4$): C (79.45, 79.40), H (5.00, 5.03), N (9.27, 9.37).

Synthesis of compound 2α: Pure product **2α** was isolated from the sample of **CP1-2α** and prepared using the same route as compound **1**. Yield 72.8% based on F-1,3-bpeb. Colourless crystals, suitable for X-ray analysis, were obtained by slow evaporation of solvents from the its solution in CH_3Cl and H_2O one day later.

^{1}H NMR (400 MHz, $CDCl_3$): δ 8.46 (d, J = 6.0 Hz, 8H), 7.09 (d, J = 6.0 Hz, 8H), 6.53 (d, J = 9.2 Hz, 4H), 6.35 (s, 2H), 4.57 (d, J = 6.0 Hz, 4H), 4.36 (d, J = 6.0 Hz, 4H); ^{13}C NMR (100 MHz, $CDCl_3$): δ 165.47, 163.00, 149.99, 148.17, 139.27, 131.02, 123.03, 110.59, 50.27, 42.97; ^{19}F NMR (377 MHz, $CDCl_3$): δ -115.05 ppm; HRMS(m/z): $[M]^+$ Calcd for $C_{40}H_{30}F_2N_4$, 605.2517; found: 604.2516; analysis (calcd., found for $C_{40}H_{30}F_2N_4$): C (79.45, 79.47), H (5.00, 5.03), N (9.27, 9.25).

Synthesis of compound 2β: Pure product **2β** was isolated from the sample of **CP1-2β** and prepared using the same route as compound **1**. Yield 86.7% based on F-1,3-bpeb. Colourless crystals, suitable for X-ray analysis, were obtained by slow evaporation of solvents from the its solution in CH_3Cl and H_2O one day later.

^{1}H NMR (400 MHz, $CDCl_3$): δ 8.47 (d, J = 6.0 Hz, 4H), 8.44 (d, J = 6.0 Hz, 4H), 7.09 (d, J = 6.0 Hz, 4H), 7.02 (d, J = 6.0 Hz, 4H), 6.77 (s, 2H), 6.57 (d, J = 9.2 Hz, 2H), 6.34 (d, J = 8.8 Hz, 2H), 4.67 (m, 8H); ^{13}C NMR (100 MHz, $CDCl_3$): δ 164.98, 162.51, 150.05, 149.91, 148.62, 148.20, 139.25, 139.18, 138.90, 138.83, 128.12,

123.15, 123.00, 113.66, 113.45, 111.25, 111.02, 52.38, 50.47, 43.19, 43.11; ^{19}F NMR (377 MHz, $CDCl_3$): δ -115.95 ppm; HRMS(m/z): $[M]^+$ Calcd for $C_{40}H_{30}F_2N_4$, 605.2517; found: 604.2515; analysis (calcd., found for $C_{40}H_{30}F_2N_4$): C (79.45, 79.43), H (5.00, 5.07), N (9.27, 9.30).

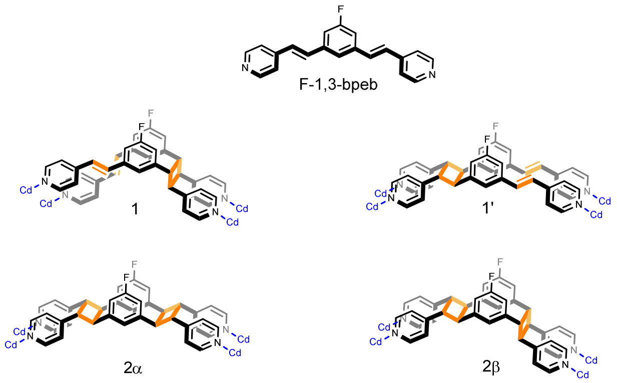

Supplementary Figure 1 | Structure determination. Model diene monomer F-1,3-bpeb and their arrangements of dimeric cyclobutane isomers in CPs.

3. X-ray data collection and structure determination

Single-crystal X-ray diffraction data for **CP1, CP1-1, CP1-2β, CP1-1'** and **CP1-2α** were obtained directly from the above preparations. Single crystals coated with Paratone oil on a Cryoloop pin were mounted on a Bruker Smart CCD diffractometer with a graphite monochromated Mo Kα radiation (λ = 0.71073 Å) at 223K. BrukerSAINT was employed for the refinement of cell parameters and the reduction of collected data, whereas absorption corrections (multi-scan) were applied. The crystal structures of these compounds were solved by Direct methods and refined by full-matrixleast-squares techniques using the *SHELXL*-2017 program[3]. The non-hydrogen atoms were refined with anisotropic displacement parameters. The lattice $CHCl_3$ molecule in **2α** was disordered over two positions with an occupancy factor of 0.5/0.5. The H atoms bonded to C and N atoms were positioned with idealized geometry and refined with fixed isotropic displacement parameters. The crystal used has cracks in this reaction, so the poor single-crystal quality of **CP1-2β** inevitably led to these B-level mistake in the Checkcif Report (PLAT342_ALERT_3_B Low Bond Precision on C-C Bonds .... 0.02996 Ang; PLAT910_ALERT_3_B Missing # of FCF Reflection(s) Below Theta(Min). 16 Note.) The positive residual density is close to that of the Cd atoms led to these B-level mistake in the Checkcif Report (PLAT973_ALERT_2_B Check Calcd Positive Resid. Density on Cd01 1.79 eA-3; PLAT973_ALERT_2_B Check Calcd Positive Resid. Density on Cd02 1.67 eA-3). A summary of the pertinent crystallographic data forthese compounds is given in Supplementary Table 1 and Supplementary Table 2. Crystallographic data for the structures reported in this Article have been deposited at the Cambridge Crystallographic Data Centre, under deposition numbers CCDC 1889155 (**CP1**), 2036562 (**CP1-1**), 2036563 (**CP1-2β**), 2036564 (**CP1-1'**), 2036565

(**CP1-2α**), 1889156 (**2β**) and 1889157 (**2α**). Copies of the data can be obtained free of charge via https://www.ccdc.cam.ac.uk/structures/.

Supplementary Table 1. Summary of crystal data and structure refinement parameters for **CP1, CP1-1, CP1-2β, CP1-1'** and **CP1-2α**

	**CP1**	**CP1-1**	**CP1-2β**	**CP1-1'**	**CP1-2α**
Emperical formula	$C_{68}H_{42}Br_8F_2N_4O_8Cd_2$	$C_{68}H_{42}Br_8F_2N_4O_8Cd_2$	$C_{136}H_{84}Br_{16}Cd_4F_4N_8O_{16}$	$C_{68}H_{42}Br_8Cd_2F_2N_4O_8$	$C_{68}H_{42}Br_8F_2N_4O_8Cd_2$
Formula weight	1945.13	1945.13	3890.27	1945.13	1945.13
Crystal system	Triclinic	Triclinic	Triclinic	Triclinic	Triclinic
Space group	$P\bar{1}$	$P\bar{1}$	$P\bar{1}$	$P\bar{1}$	$P\bar{1}$
a/Å	9.188(4)	9.0355(10)	17.2777(19)	9.6781(16)	9.2870(9)
b/Å	16.712(6)	16.7709(18)	18.363(2)	15.818(3)	15.7041(15)
c/Å	23.240(9)	23.325(2)	22.448(3)	23.273(4)	23.904(2)
α/°	88.441(10)	88.315(3)	85.563(3)	87.523(5)	86.913(3)
β/°	83.886(11)	83.794(3)	88.540(3)	88.828(4)	88.092(3)
γ/°	75.612(10)	74.989(3)	74.715(3)	73.688(4)	75.968(3)
V/Å^3	3437.0(2)	3393.8(6)	6849.3(13)	3416.1(10)	3376.6(6)
D_c/g cm^{-3}	1.880	1.903	1.886	1.891	1.913
Z	2	2	2	2	2
μ (Mo-Kα)/mm^{-1}	5.331	5.399	5.350	5.363	5.426
Total reflections	26545	87884	121803	71314	84889
Unique reflections	13579	15623	24035	12043	15406
No. observations	7442	9247	11817	5910	8448
No. parameters	857	838	1669	849	829
F(000)	1872.0	1872.0	3744.0	1872.0	1872.0
R_1^{a}	0.0525	0.0613	0.1009	0.0583	0.0676
wR_2^{b}	0.1172	0.1268	0.3151	0.1639	0.1844
GOF^c	0.951	1.009	1.055	1.016	1.021

$^a R_1 = \Sigma||F_0|-|F_c||/\Sigma|F_0|$. $^b wR_2 = \{\Sigma w(F_0^2-F_c^2)^2/\Sigma w(F_0^2)^2\}^{1/2}$. $^c \mathrm{GOF} = \{\Sigma w((F_0^2-F_c^2)^2)/(n-p)\}^{1/2}$, where n = number of reflections and p = total number of parameters refined.

Supplementary Table 2. Summary of crystal data and structure refinement parameters for **2α** and **2β**

	**2α**	**2β**
Emperical formula	$C_{82}H_{68}Cl_6F_4N_8O_3$	$C_{40}H_{36}F_2N_4O_3$
Formula weight	1502.14	658.73
Crystal system	monoclinic	monoclinic
Space group	$P2_1/c$	$P2_1/c$
a/Å	14.3270(11)	19.1539(8)
b/Å	17.7425(13)	16.2082(7)
c/Å	18.7927(11)	10.7396(4)
α/°	90	90
β/°	130.578(4)	105.334(10)
γ/°	90	90
V/Å^3	3628.3(5)	3215.4(2)
D_c/g cm^{-3}	1.375	1.361
Z	2	4
μ (Mo-Kα)/mm^{-1}	0.0304	0.095
Total reflections	41469	28754
Unique reflections	6375	5634
No. observations	4816	3769
No. parameters	496	442
F(000)	1556.0	1384.0
$R_1{}^a$	0.0602	0.0517
$wR_2{}^b$	0.1780	0.1340
GOF^c	1.027	1.044

$^aR_1 = \Sigma||F_0|-|F_c||/\Sigma|F_0|$. $^bwR_2 = \{\Sigma w(F_0{}^2-F_c{}^2)^2/\Sigma w(F_0{}^2)^2\}^{1/2}$. ^{c}GOF = $\{\Sigma w((F_0{}^2-F_c{}^2)^2)/(n-p)\}^{1/2}$, where n = number of reflections and p = total number of parameters refined.

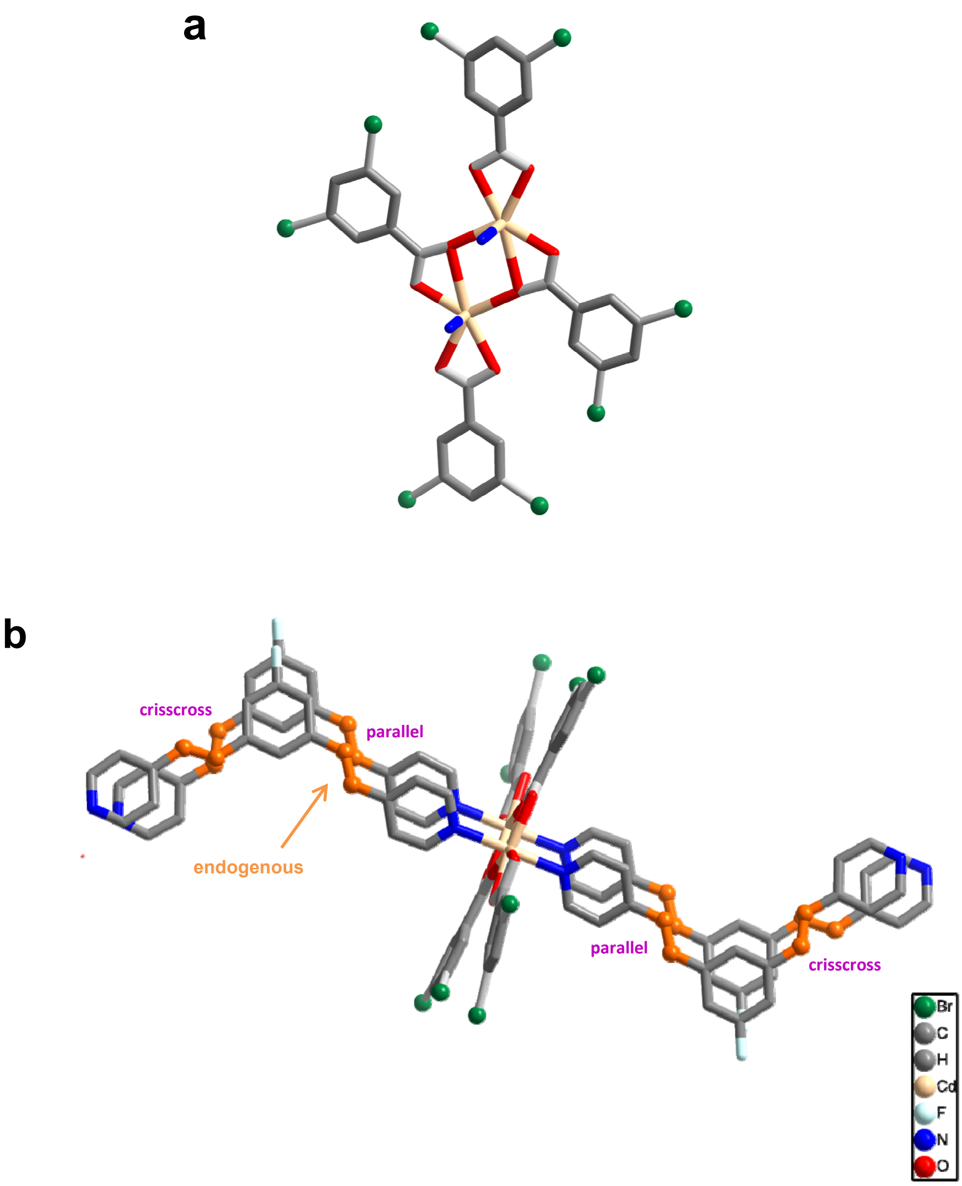

Supplementary Figure 2 | Coordination polymer structure. a Representation of the coordination environment of the Cd(II) ions in **CP1**. **b** View of a section of 1D chain structure of **CP1**, showing the face-to-face alignments of two F-1,3-bpeb ligands with one parallel pair of olefinic groups and the other crisscross pair of olefinic groups. Gray, blue, red, sky blue, dark green and pale yellow spheres represent C, N, O, F, Br and Cd atoms, respectively. Hydrogen atoms have been omitted for clarity.

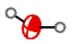

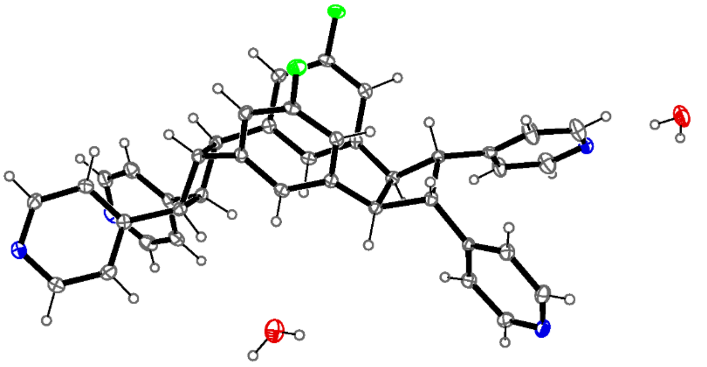

Supplementary Figure 3 | The crystal structure of **2β**.

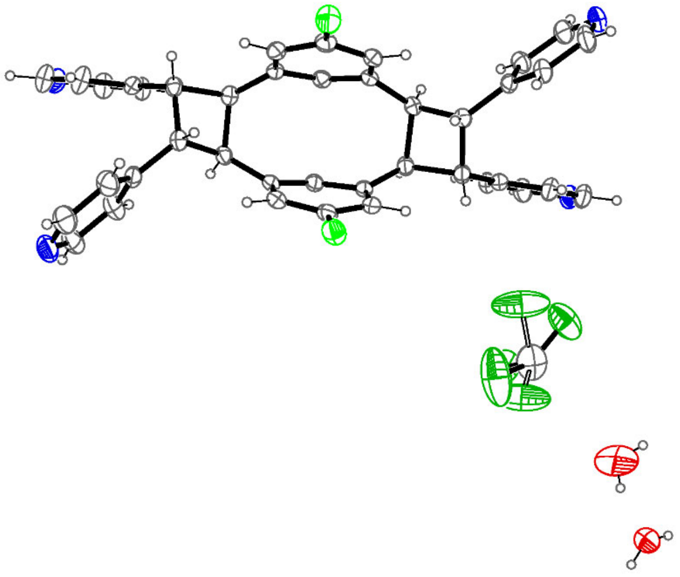

Supplementary Figure 4 | The crystal structure of **2α**.

4. Solution NMR spectra of F-1,3-bpeb, digested CPs and isomeric cyclic compounds.

The NMR spectra were recorded at ambient temperature on a Bruker AVANCEШ HD-400M spectrometer. ^{1}H NMR and ^{19}F NMR chemical shifts were referenced to the solvent signal in $CDCl_3$ or DMSO-d_6. Chemical shifts are reported in parts per million (ppm) and referenced with TMS for ^{1}H NMR and $CFCl_3$ for ^{19}F NMR.

Coordinated 3,5-dibromobenzoate (3,5-DBB) acted as modulator to control arrangement of F-1,3-bpeb in **CP1**. 3,5-DBB was retained in the final structure of the metal organic framework. Integrating the signals related to 3,5-DBB and F-1,3-bpeb molecules it is possible to estimate the ratio between 3,5-DBB and F-1,3-bpeb inside metal organic frameworks.

$$\frac{I_{F-1,3-bpeb}}{I_{3,5-DBB}} = \frac{F-1,3-bpeb}{3,5-DBB} * m_R = 1$$

where

$I_{F-1,3-bpeb}$ = *integral value of peak centered at 8.59 ppm.*

$I_{3,5-DBB}$ = *integral value of peak centered at 8.03 ppm.*

$\frac{F-1,3-bpeb}{3,5-DBB}$ = *molar ratio between 3,5-DBB and F-1,3-bpeb moieties in solution.*

a

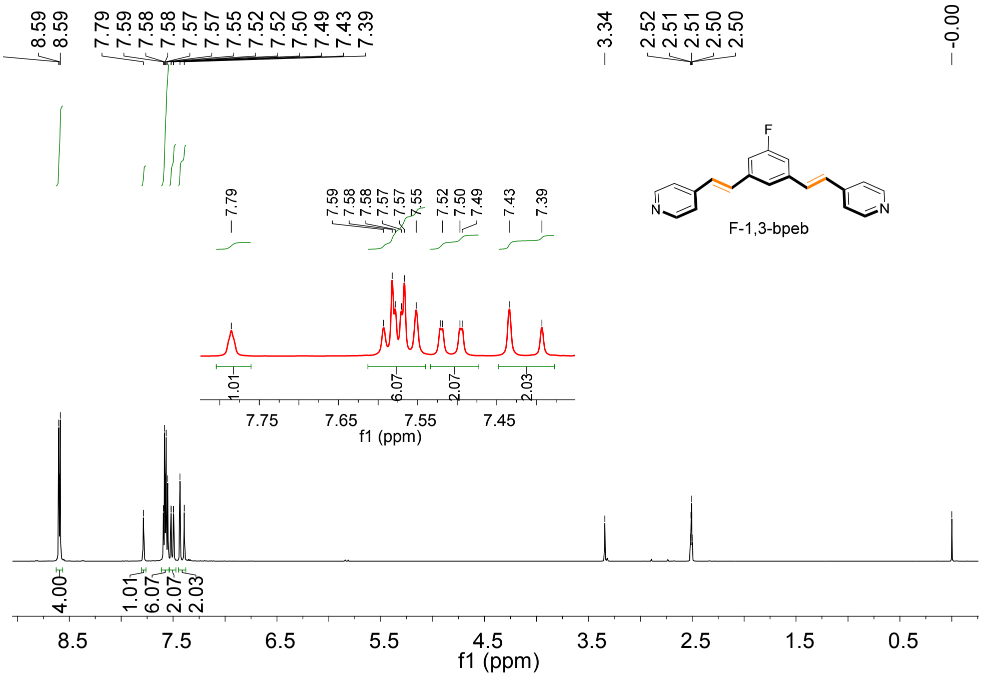

8.59
8.59
7.79
7.59
7.58
7.58
7.57
7.57
7.55
7.52
7.52
7.50
7.49
7.43
7.39
3.34
2.52
2.51
2.51
2.50
2.50
-0.00
F
N
N
F-1,3-bpeb
1.01
6.07
2.07
2.03
7.75
7.65
7.55
7.45
f1 (ppm)
4.00
8.5
7.5
6.5
5.5
4.5
3.5
2.5
1.5
0.5
f1 (ppm)

b

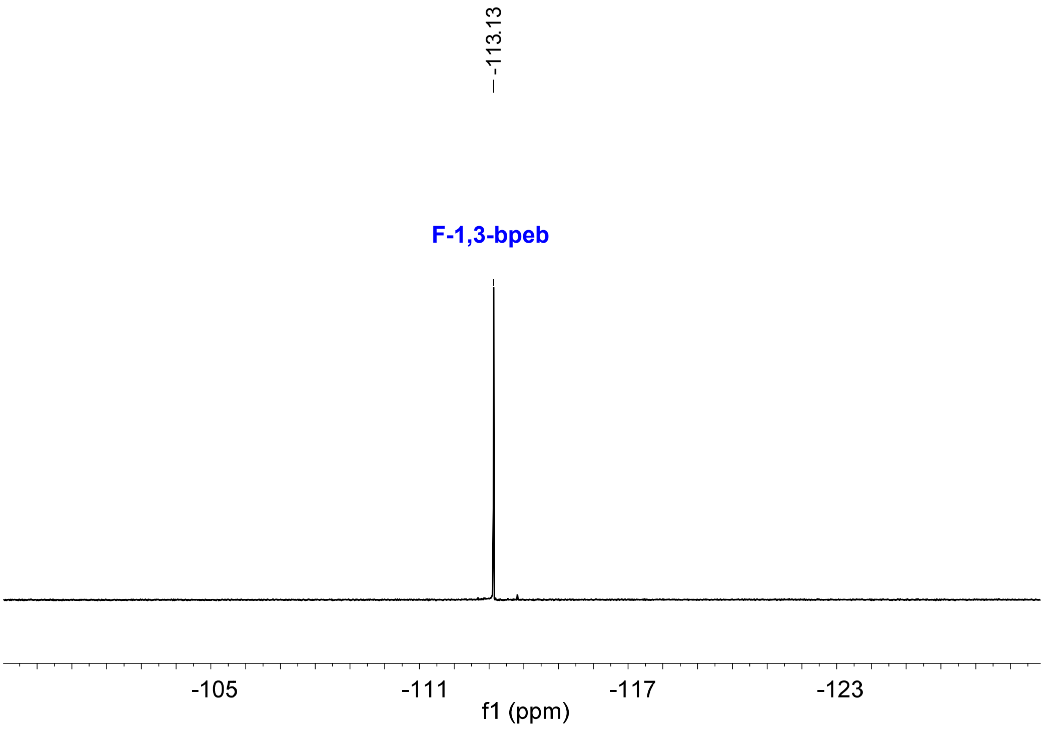

-113.13
F-1,3-bpeb
-105
-111
-117
-123
f1 (ppm)

c

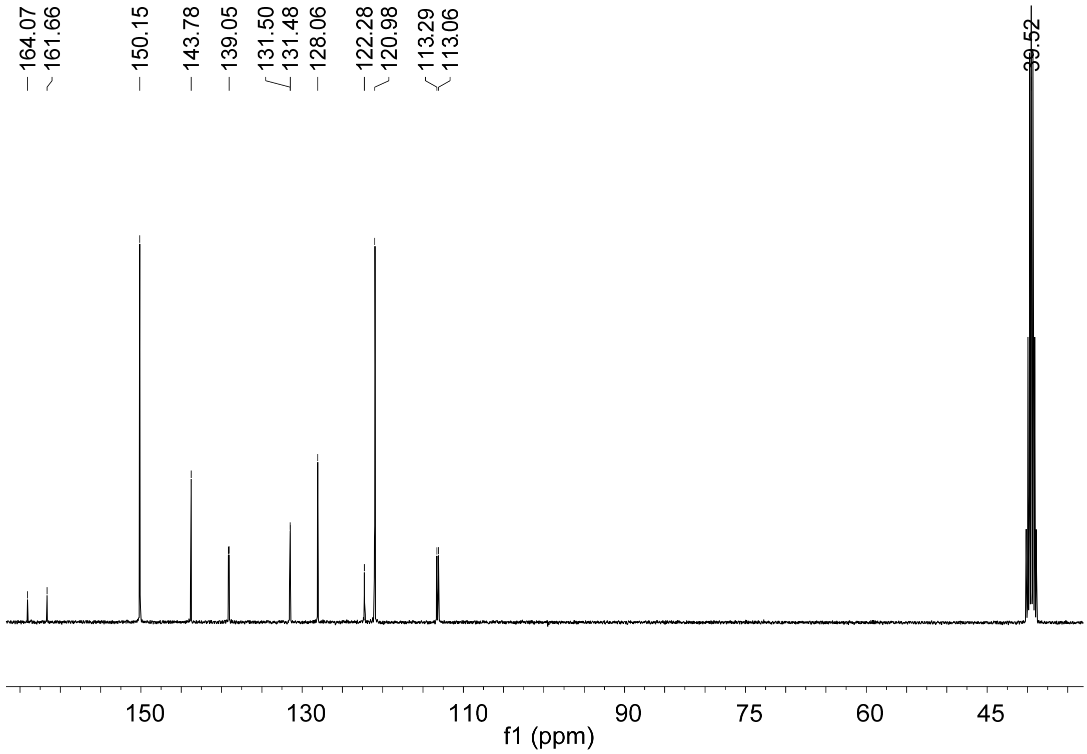

Supplementary Figure 5 | The NMR spectra of F-1,3-bpeb in DMSO-d_6. a ^{1}H. **b** ^{19}F. **c** ^{13}C.

a

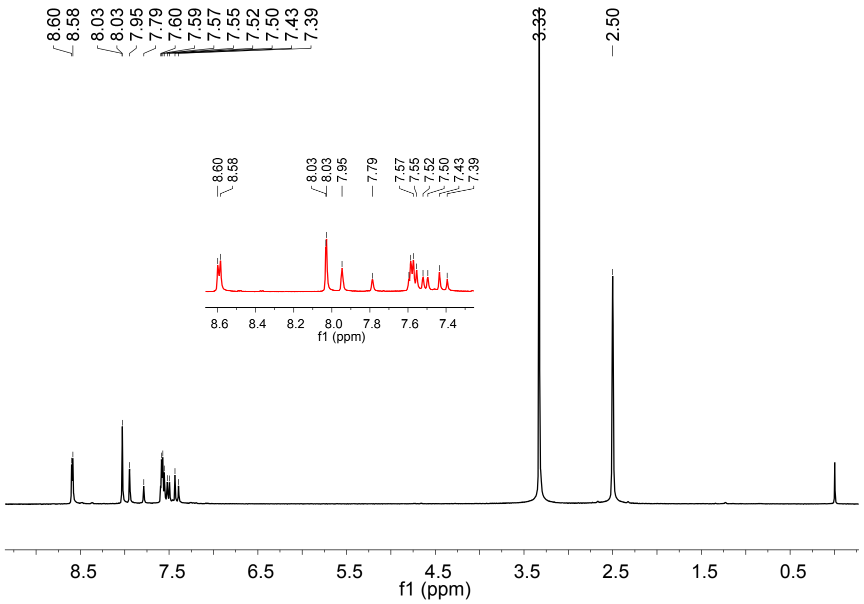

b

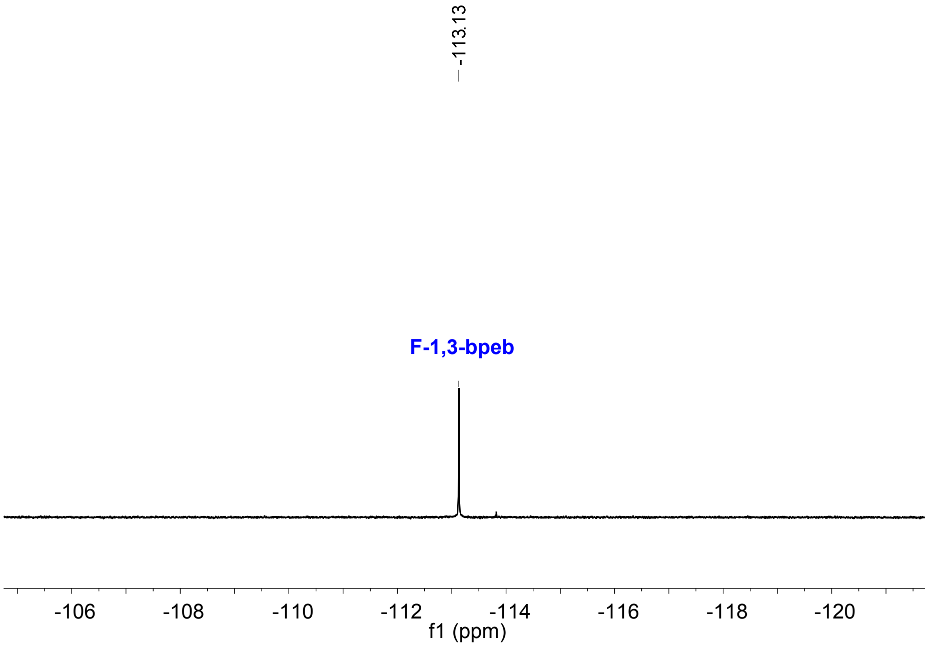

Supplementary Figure 6 | The NMR spectra of **CP1** before UV irradiation in DMSO-d_6. **a** ^{1}H. **b** ^{19}F.

a

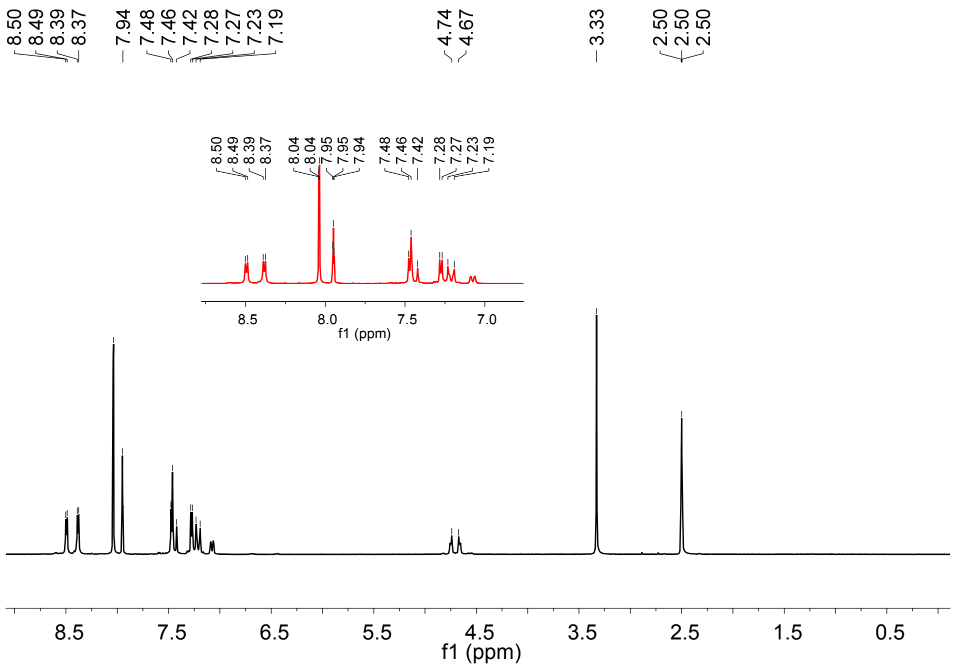

b

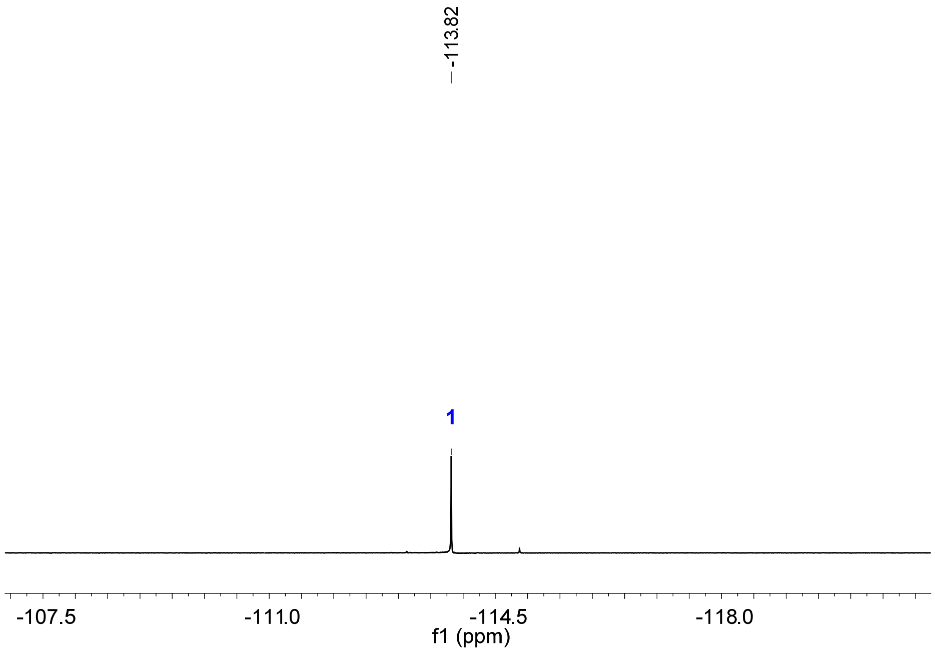

Supplementary Figure 7 | The NMR spectra of the sample obtained from **CP1** irradiated under UV light (λ = 365 nm) at -50 ℃ in DMSO-d_6. **a** ^{1}H. **b** ^{19}F.

a

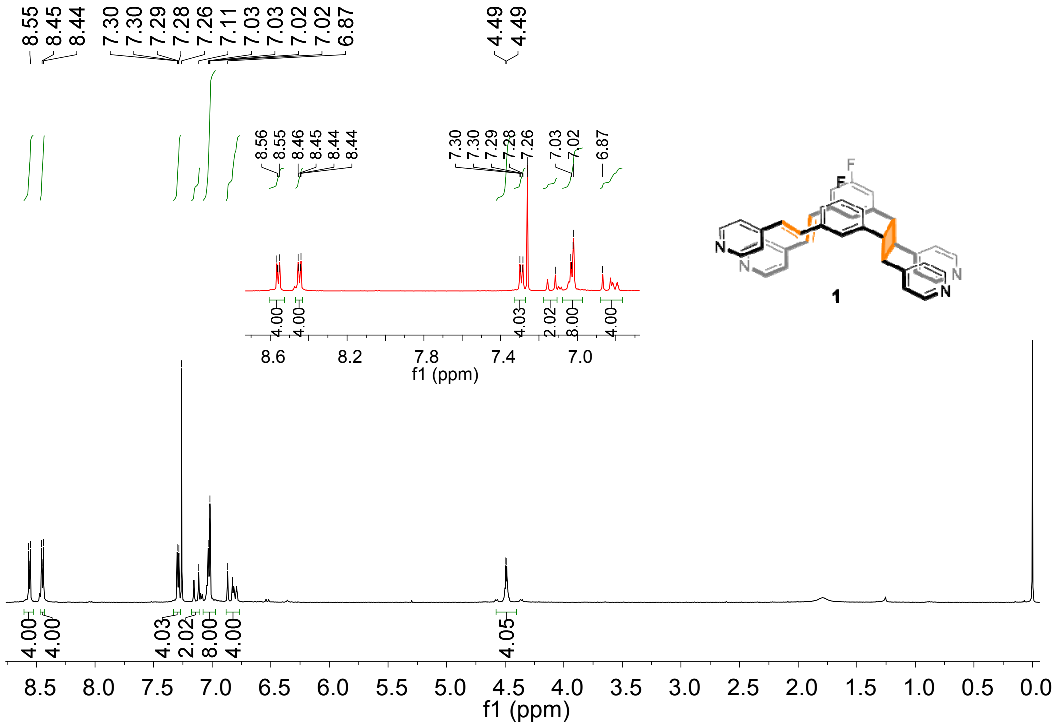

8.55
8.45
8.44
7.30
7.30
7.29
7.28
7.26
7.11
7.03
7.03
7.02
7.02
6.87
4.49
4.49
1
f1 (ppm)

b

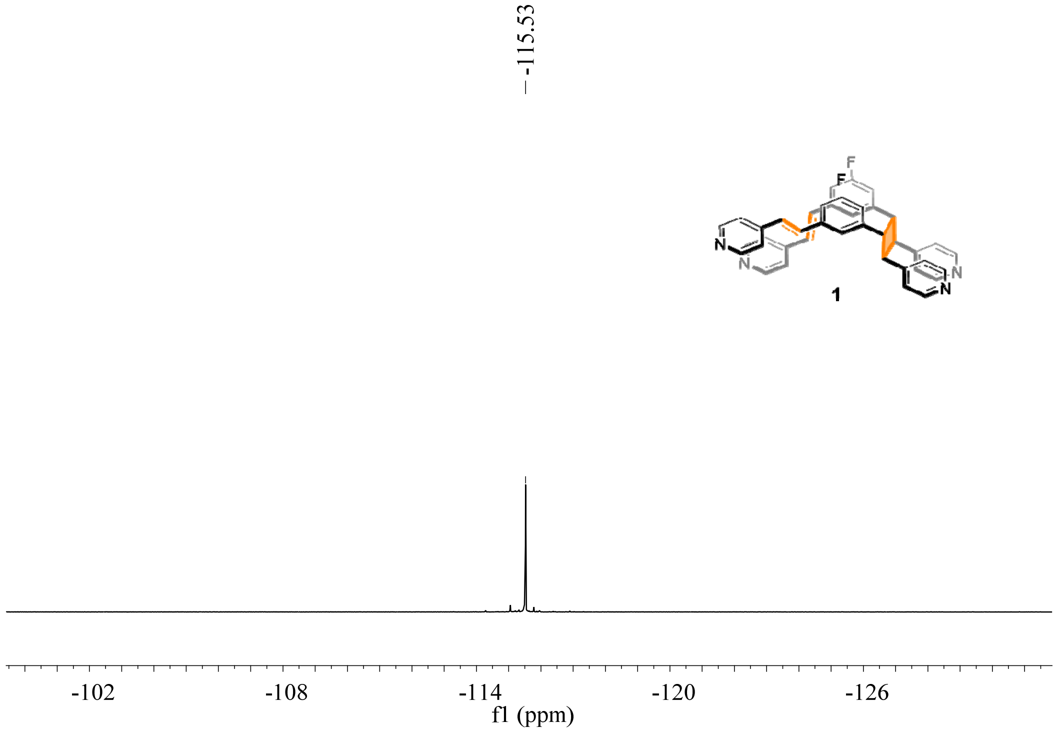

-115.53
1
-102
-108
-114
-120
-126
f1 (ppm)

c

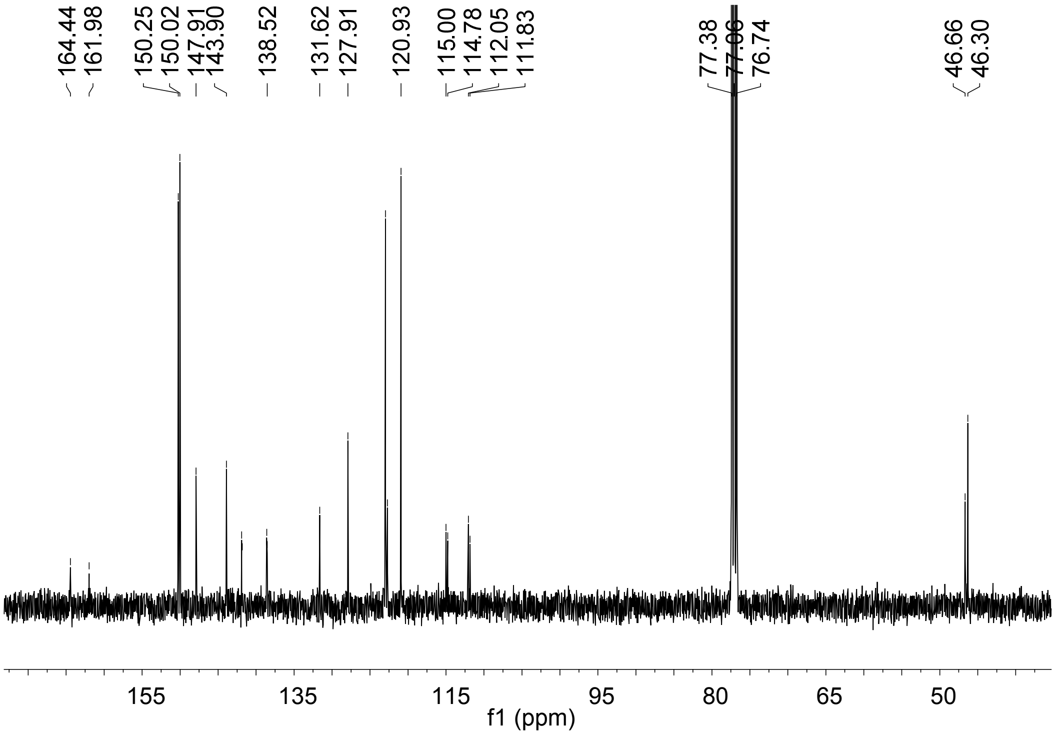
164.44
161.98
150.25
150.02
147.91
143.90
138.52
131.62
127.91
120.93
115.00
114.78
112.05
111.83
77.38
77.06
76.74
46.66
46.30
155
135
115
95
80
65
50
f1 (ppm)

d

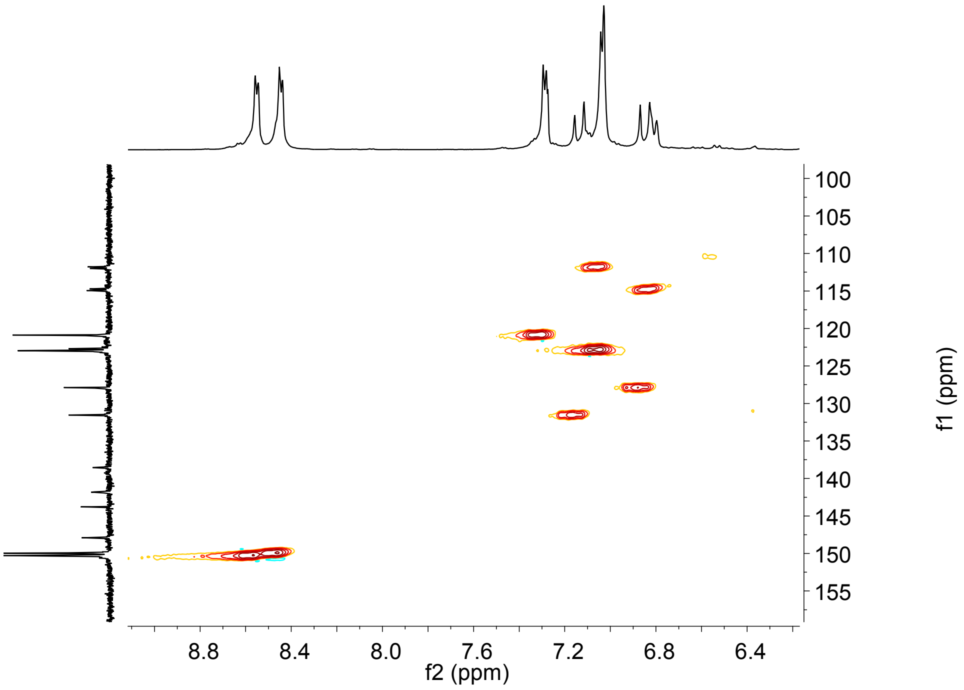
8.8
8.4
8.0
7.6
7.2
6.8
6.4
f2 (ppm)
100
105
110
115
120
125
130
135
140
145
150
155
f1 (ppm)

e

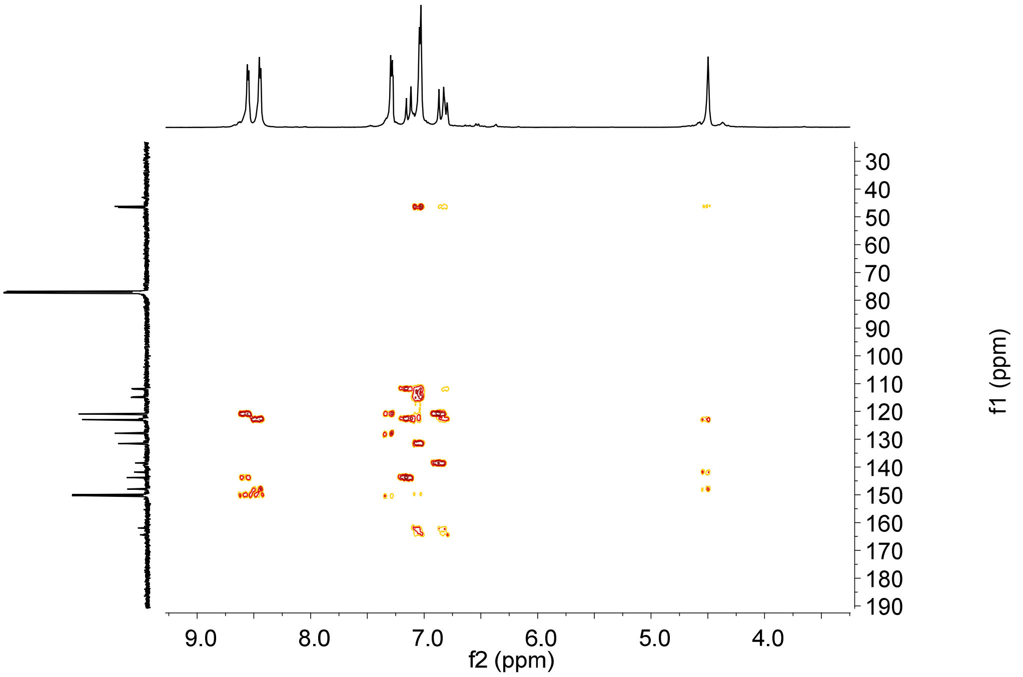

f

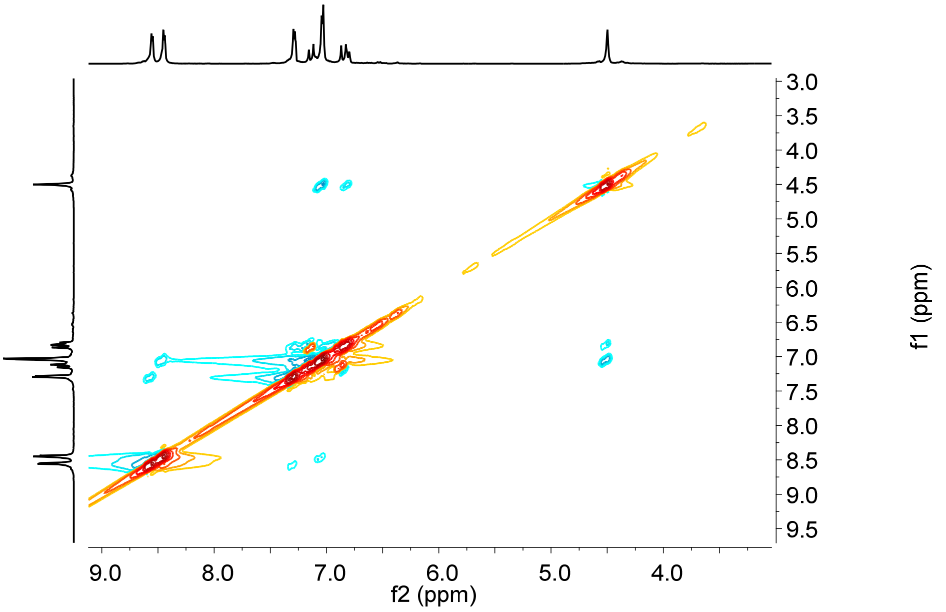

Supplementary Figure 8 | The NMR spectra of the sample isolated from decomposed **CP1-1** in $CDCl_3$. **a** ^{1}H. **b** ^{19}F. **c** ^{13}C. **d** HSQC. **e** HMBC. **f** NOESY.

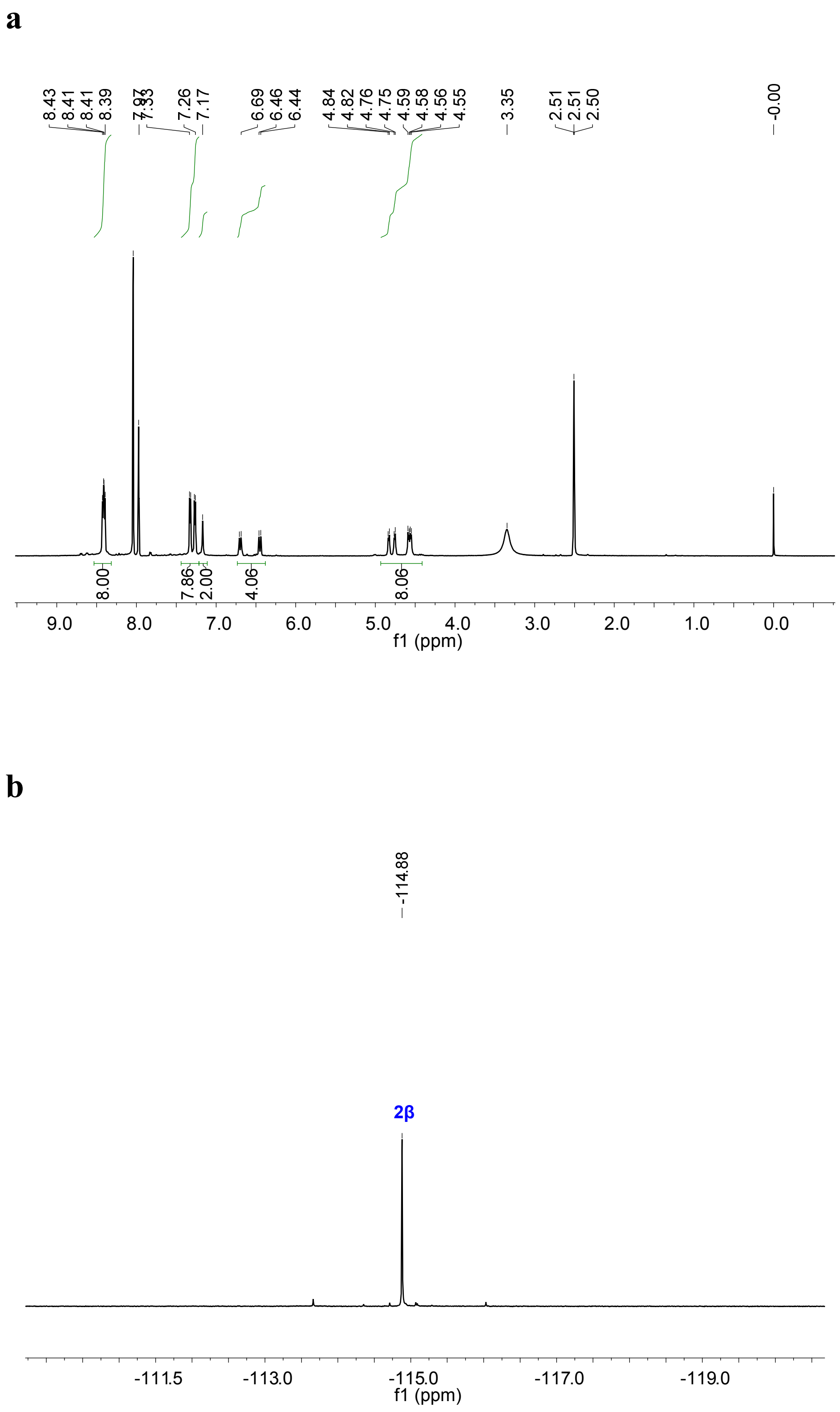

Supplementary Figure 9 | **The NMR spectra of the sample obtained from **CP1 under UV light at 25℃ (λ = 365 nm) in DMSO-d_6. **a** ^{1}H. **b** ^{19}F.

a

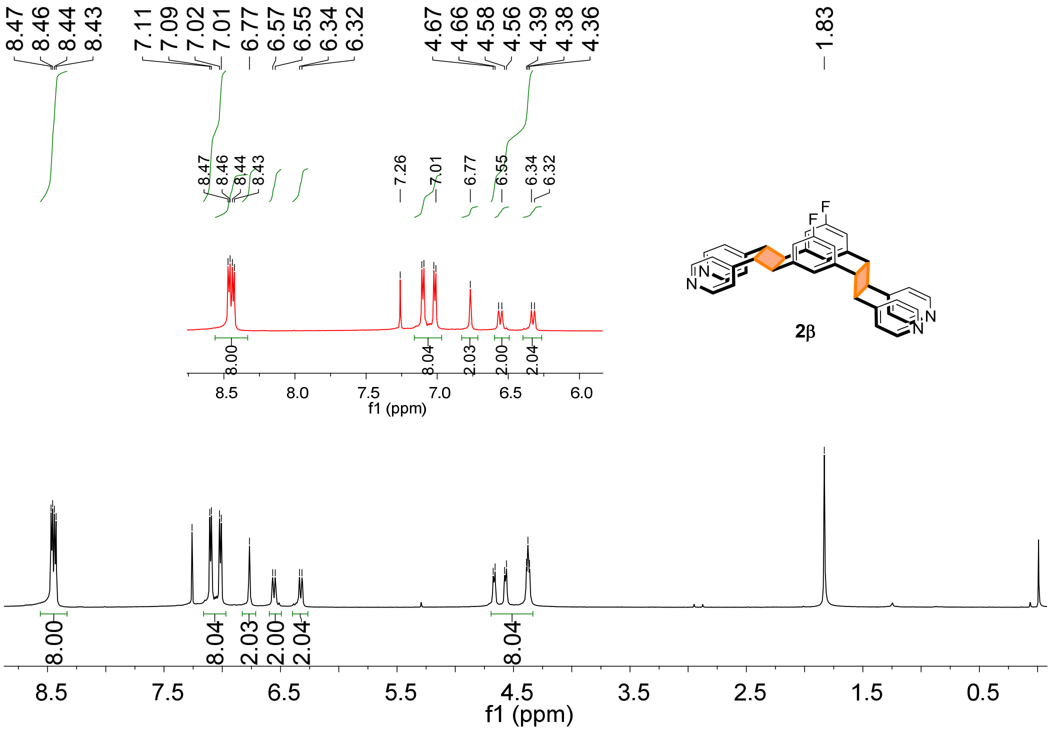
8.47
8.46
8.44
8.43
7.11
7.09
7.02
7.01
6.77
6.57
6.55
6.34
6.32
4.67
4.66
4.58
4.56
4.39
4.38
4.36
1.83
7.26
8.00
8.04
2.03
2.00
2.04
f1 (ppm)
F
N
2β

b

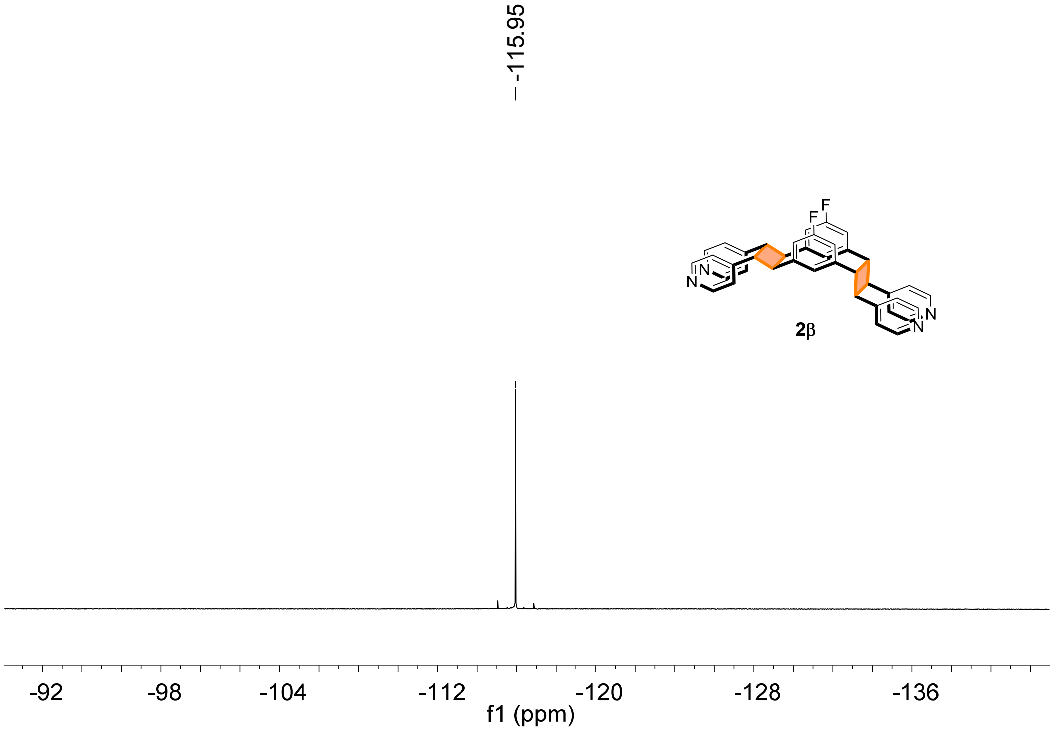
-115.95
F
N
2β
-92
-98
-104
-112
-120
-128
-136
f1 (ppm)

c

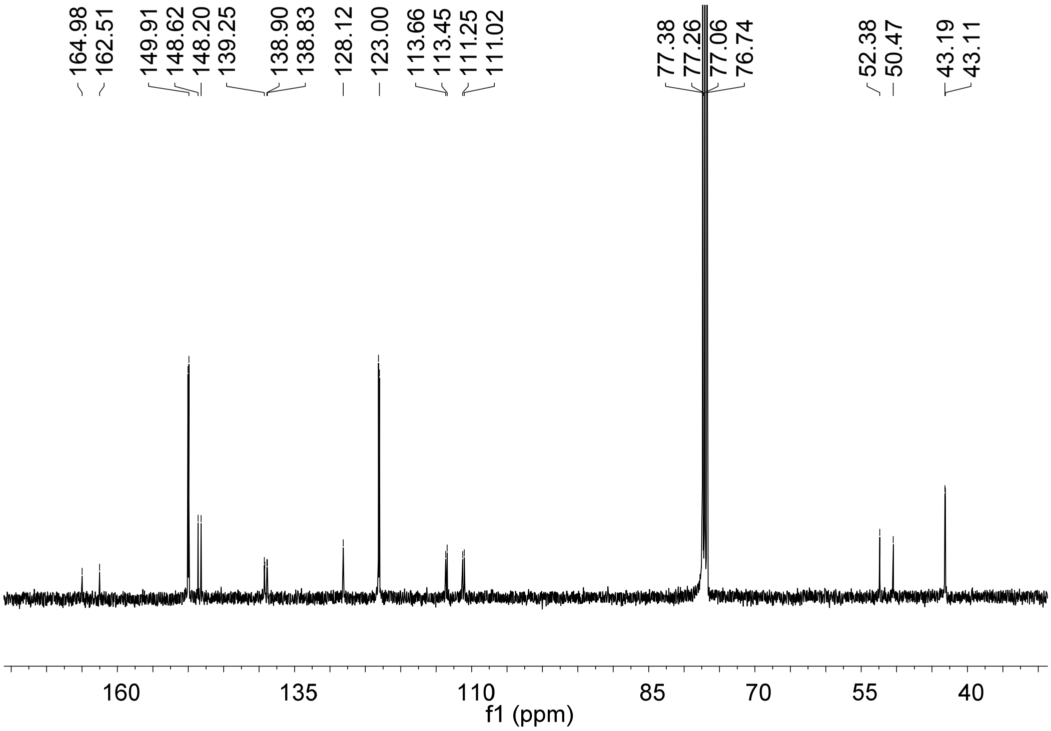
164.98
162.51
149.91
148.62
148.20
139.25
138.90
138.83
128.12
123.00
113.66
113.45
111.25
111.02
77.38
77.26
77.06
76.74
52.38
50.47
43.19
43.11
160
135
110
85
70
55
40
f1 (ppm)

d

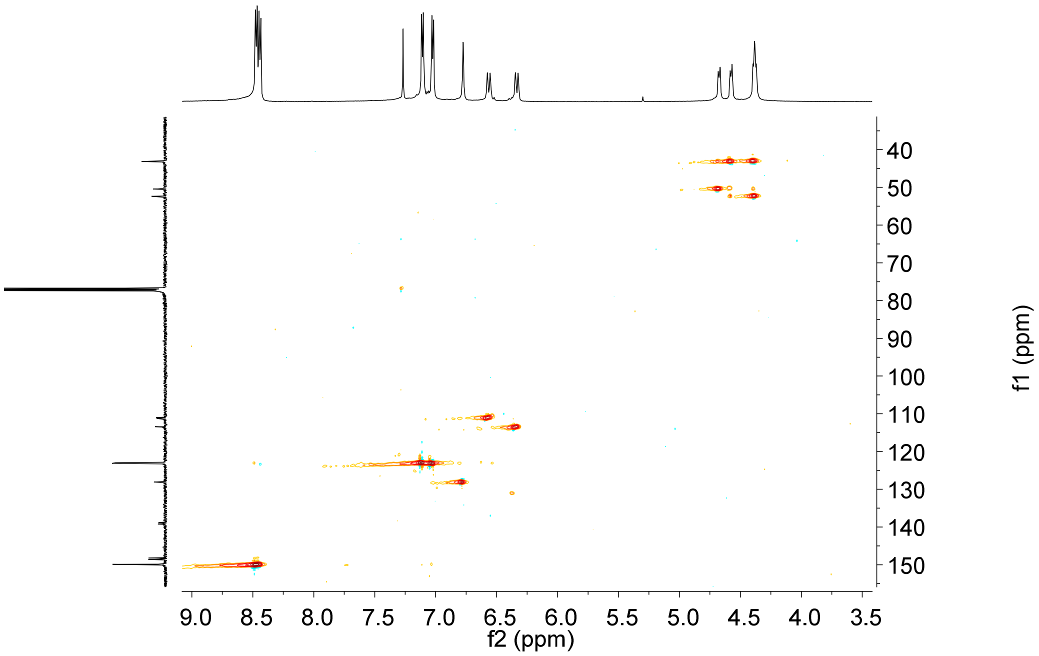
40
50
60
70
80
90
100
110
120
130
140
150
f1 (ppm)
9.0
8.5
8.0
7.5
7.0
6.5
6.0
5.5
5.0
4.5
4.0
3.5
f2 (ppm)

e

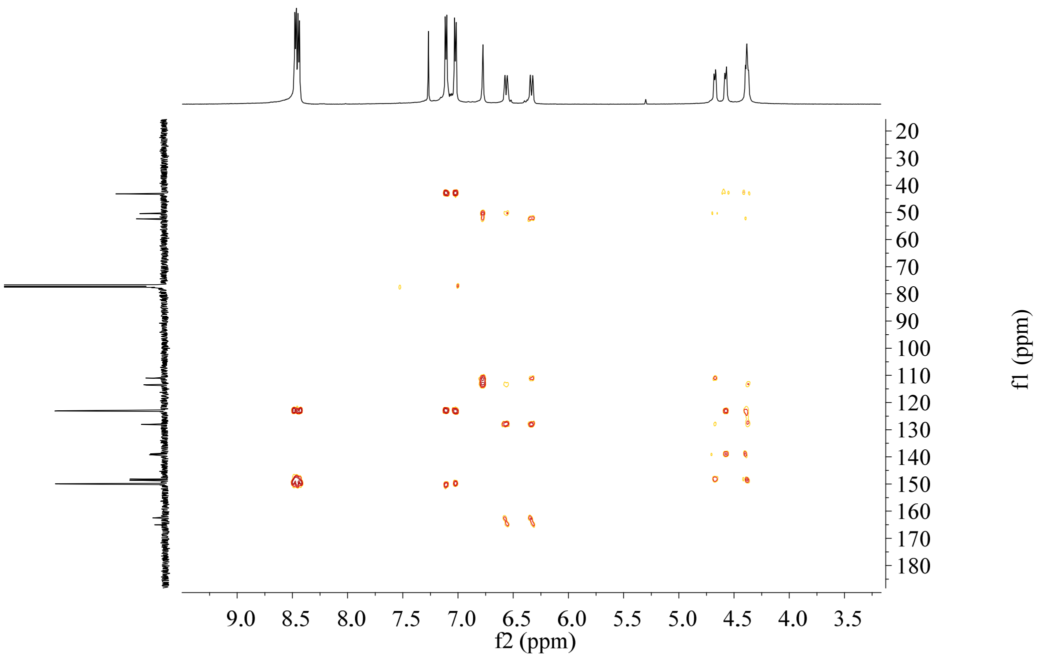

f

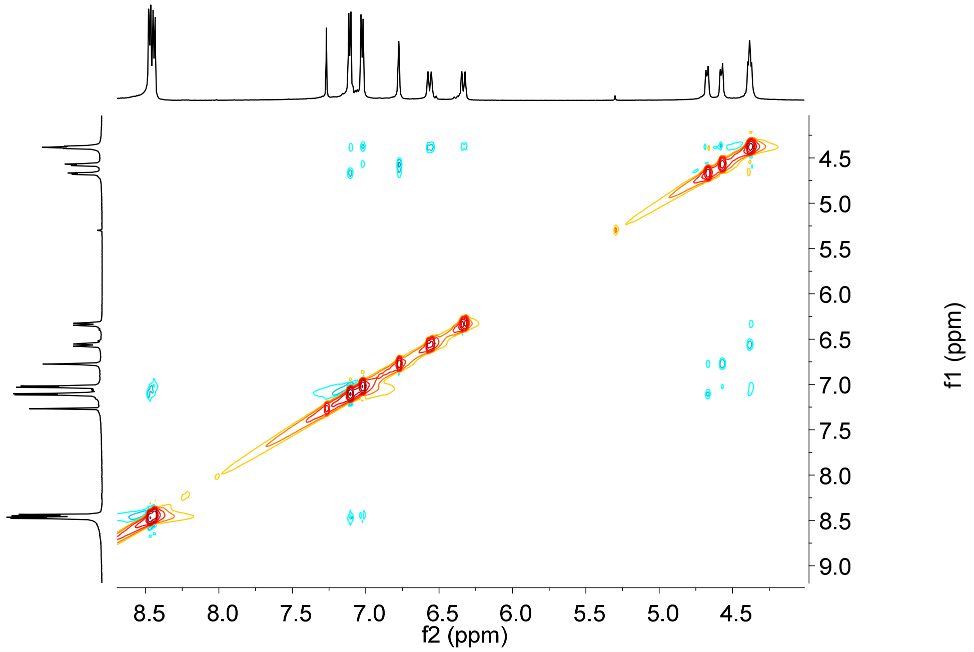

Supplementary Figure 10 | The NMR spectra of the sample isolated from decomposed **CP1-2β** in $CDCl_3$. **a** ^{1}H. **b** ^{19}F. **c** ^{13}C. **d** HSQC. **e** HMBC. **f** NOESY.

a

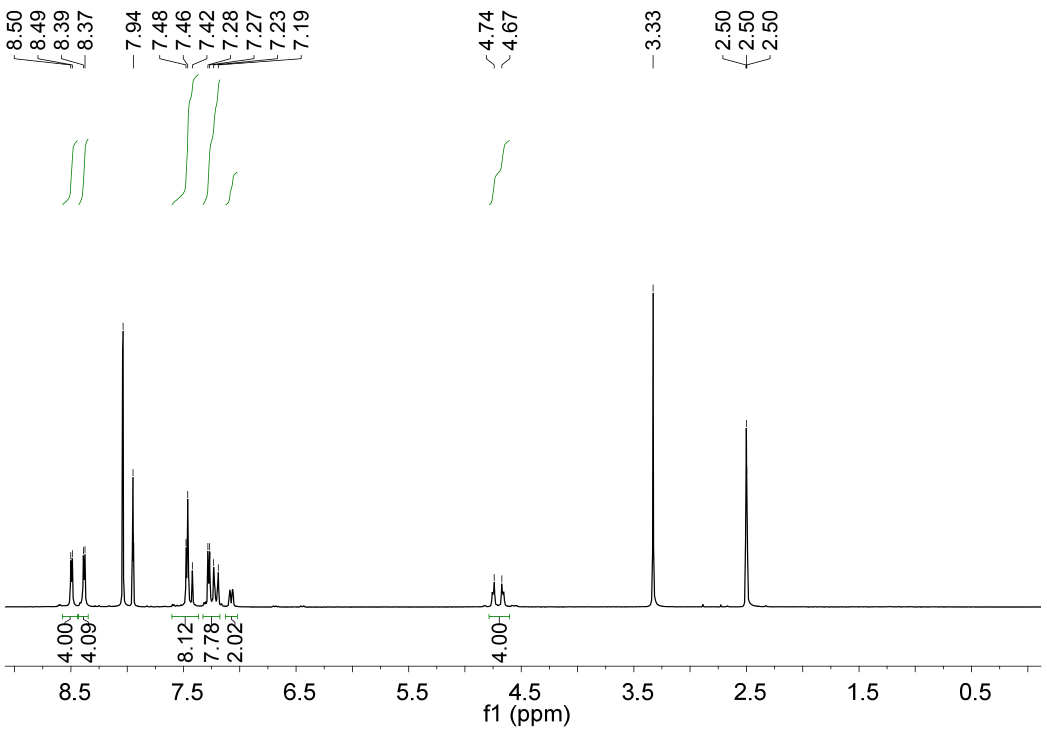

b

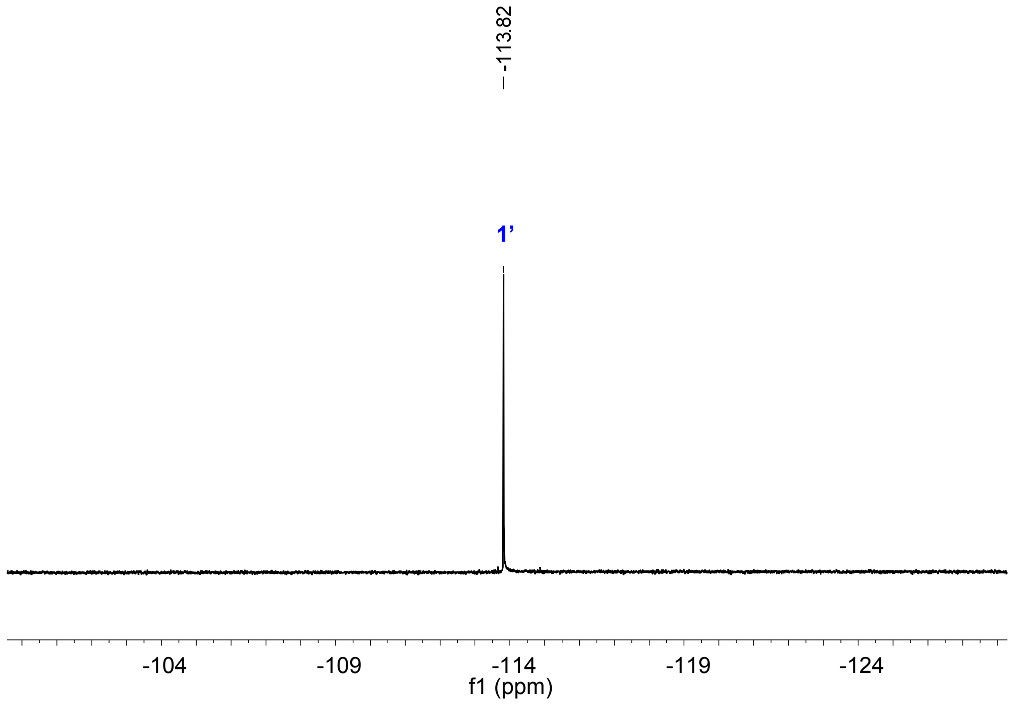

Supplementary Figure 11 | The NMR spectra of the sample obtained from **CP1-2β** heated at 208℃ for 4h in DMSO-d_6. **a** ^{1}H. **b** ^{19}F.

a

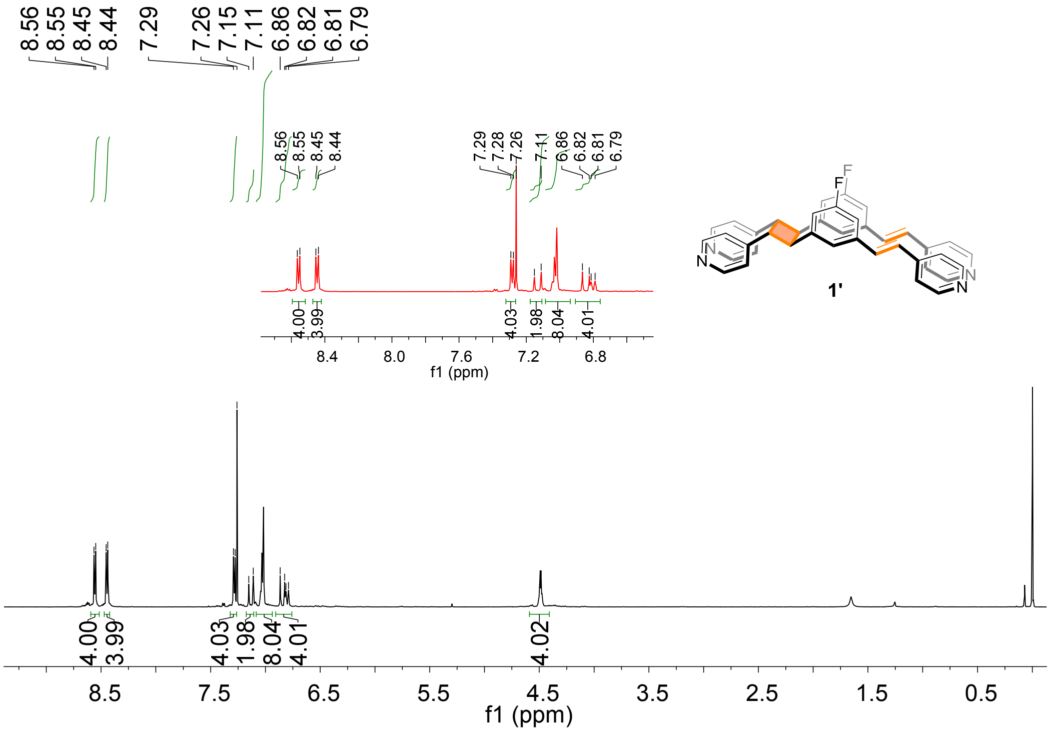
8.56 8.55 8.45 8.44 7.29 7.26 7.15 7.11 6.86 6.82 6.81 6.79
8.56 8.55 8.45 8.44
7.29 7.28 7.26 7.11 6.86 6.82 6.81 6.79
4.00 3.99 4.03 1.98 8.04 4.01
8.4 8.0 7.6 7.2 6.8
f1 (ppm)
F
N
N
1'
4.00 3.99 4.03 1.98 8.04 4.01 4.02
8.5 7.5 6.5 5.5 4.5 3.5 2.5 1.5 0.5
f1 (ppm)

b

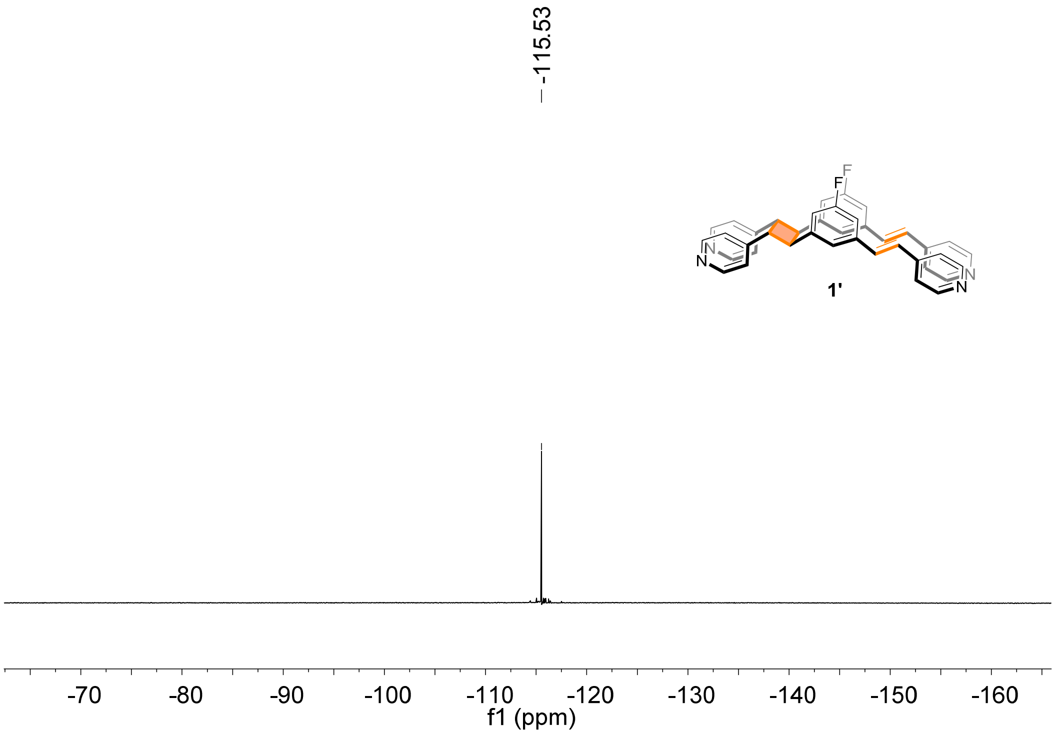
-115.53
F
N
N
1'
-70 -80 -90 -100 -110 -120 -130 -140 -150 -160
f1 (ppm)

c

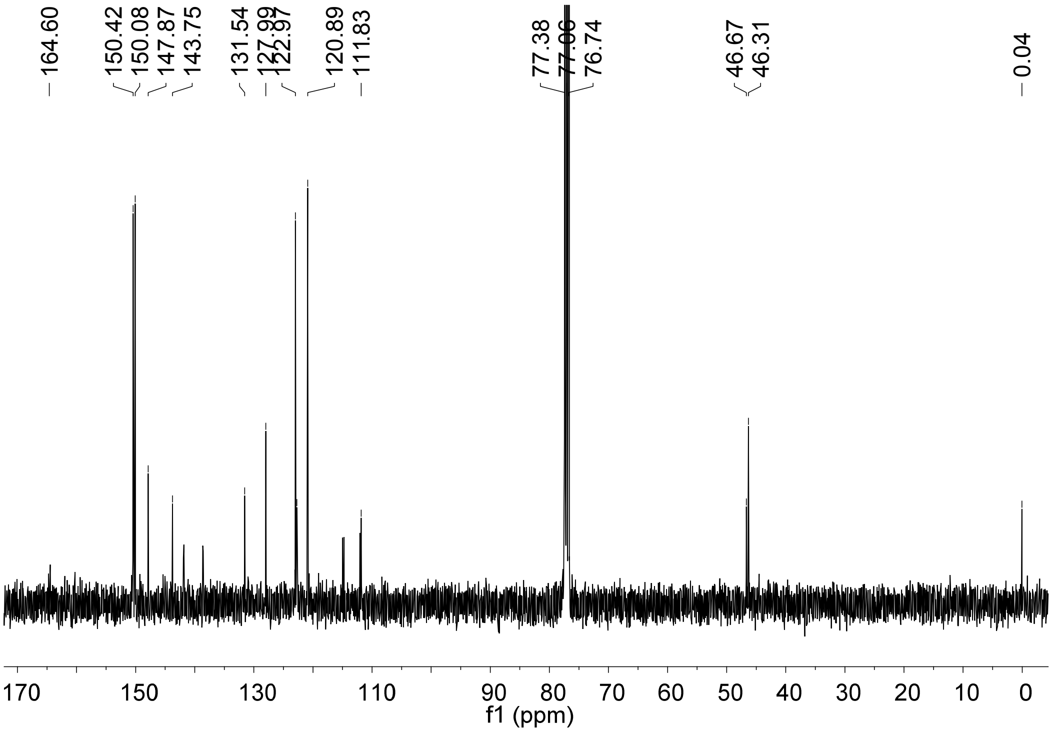
164.60
150.42
150.08
147.87
143.75
131.54
127.99
122.97
120.89
111.83
77.38
77.06
76.74
46.67
46.31
-0.04
170 150 130 110 90 80 70 60 50 40 30 20 10 0
f1 (ppm)

d

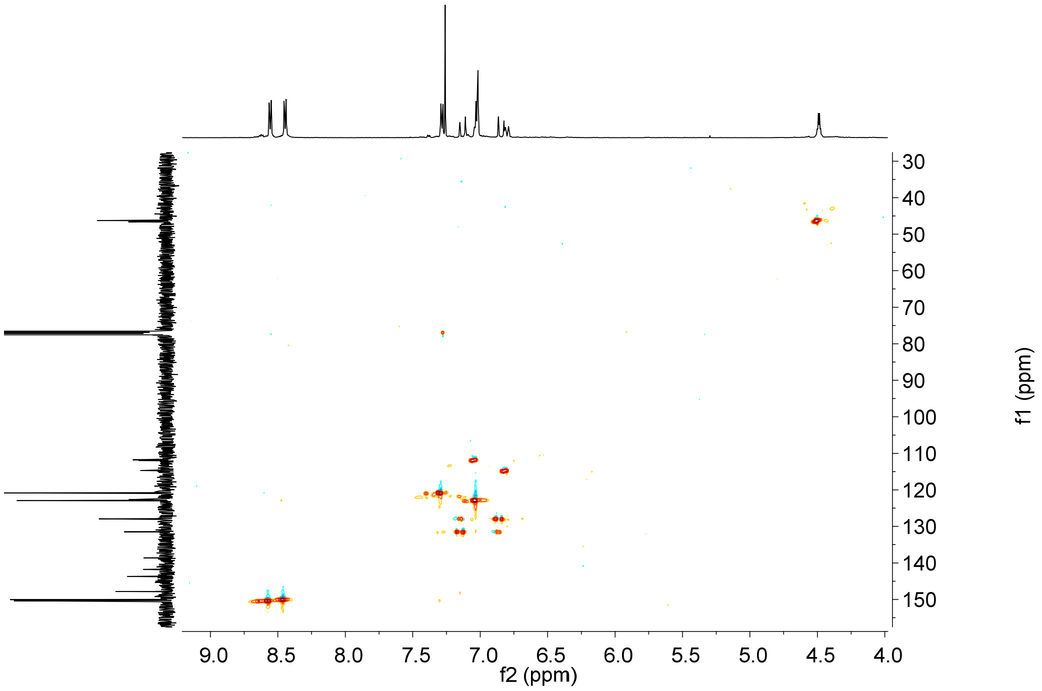
30 40 50 60 70 80 90 100 110 120 130 140 150
f1 (ppm)
9.0 8.5 8.0 7.5 7.0 6.5 6.0 5.5 5.0 4.5 4.0
f2 (ppm)

e

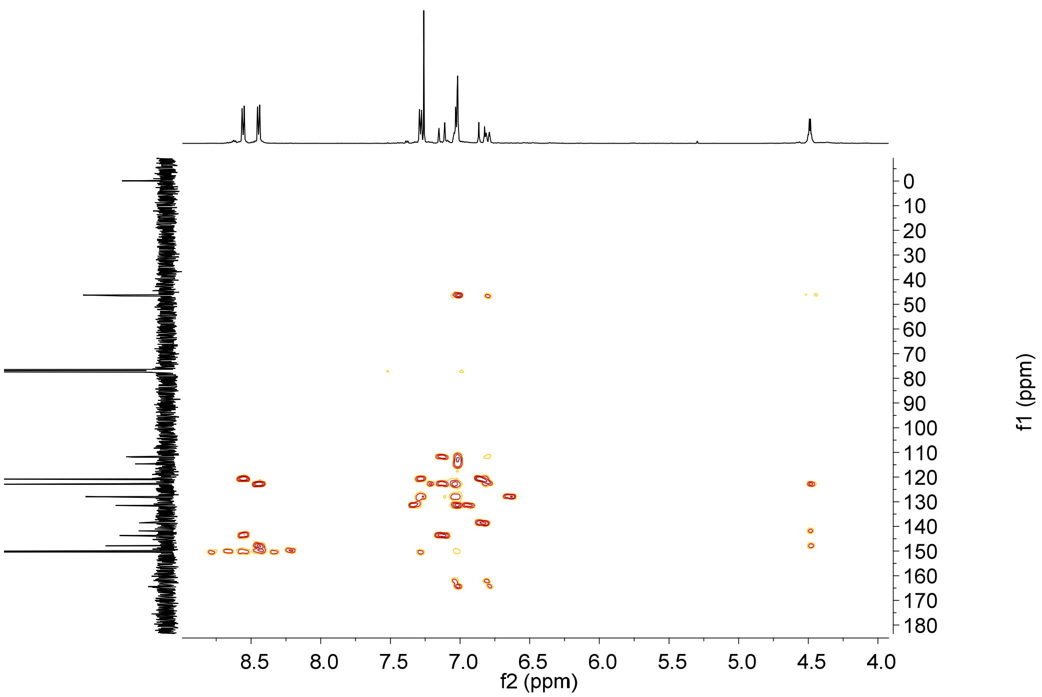

f

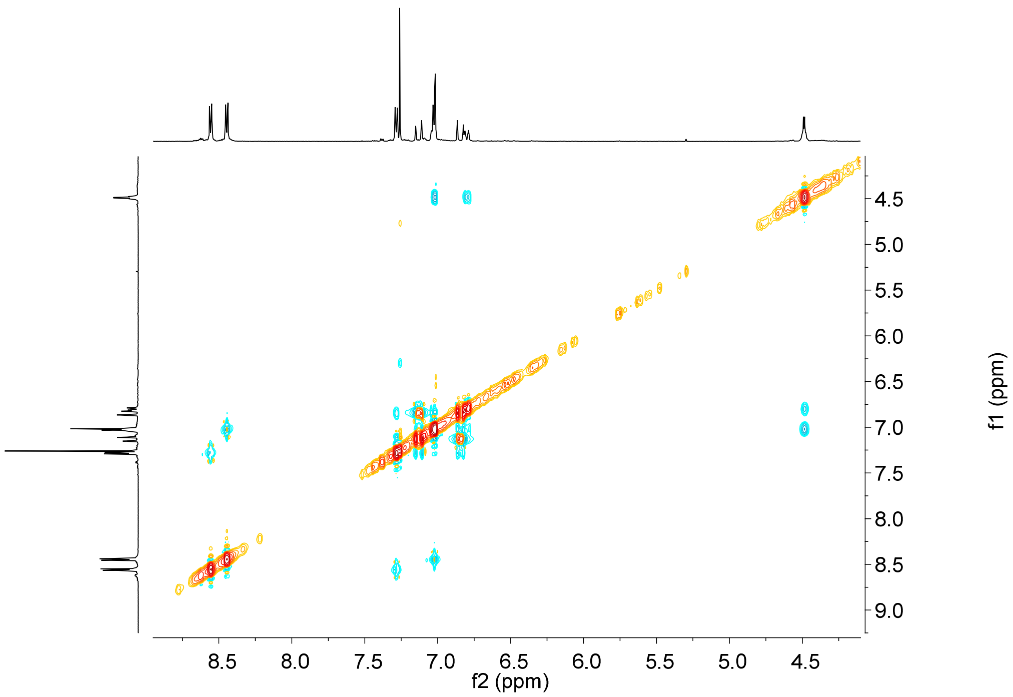

Supplementary Figure 12 | The NMR spectra of the sample isolated from decomposed **CP1-1'** in $CDCl_3$. **a** ^{1}H. **b** ^{19}F. **c** ^{13}C. **d** HSQC. **e** HMBC. **f** NOESY.

a

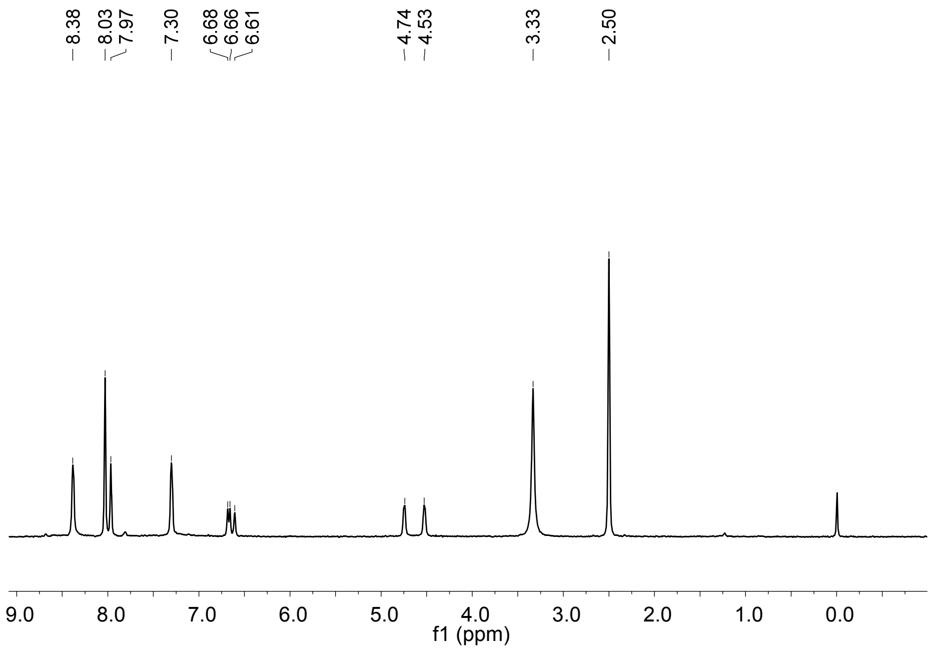

b

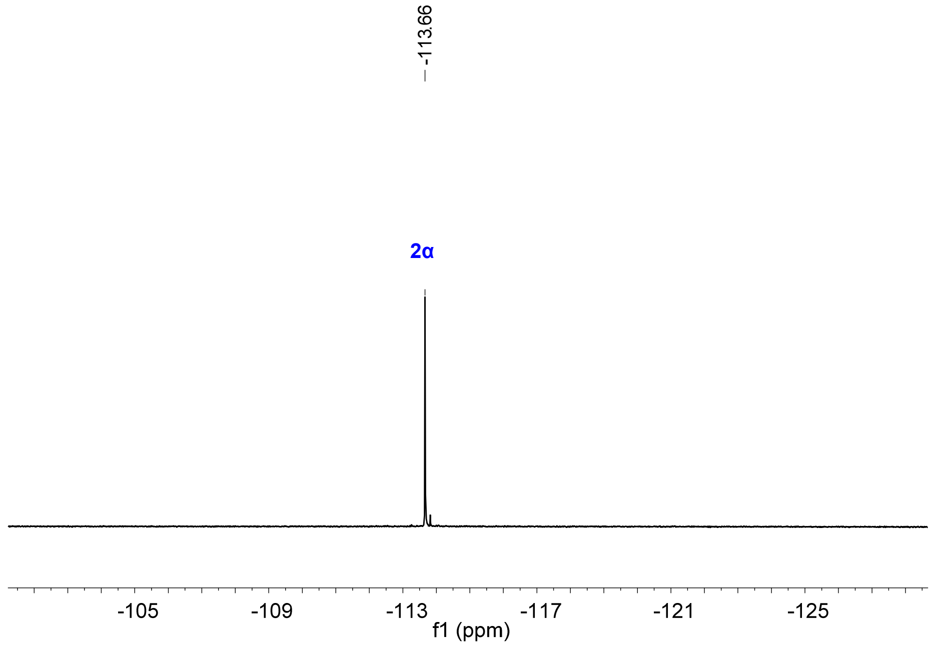

Supplementary Figure 13 | The NMR spectra of the sample obtained from **CP1** irradiated under UV light at 208 ℃ for 3 h or **CP1-1'** irradiated under UV light at 25℃ for 1.5 h (λ = 365 nm) in DMSO-d_6. **a** ^{1}H. **b** ^{19}F.

a

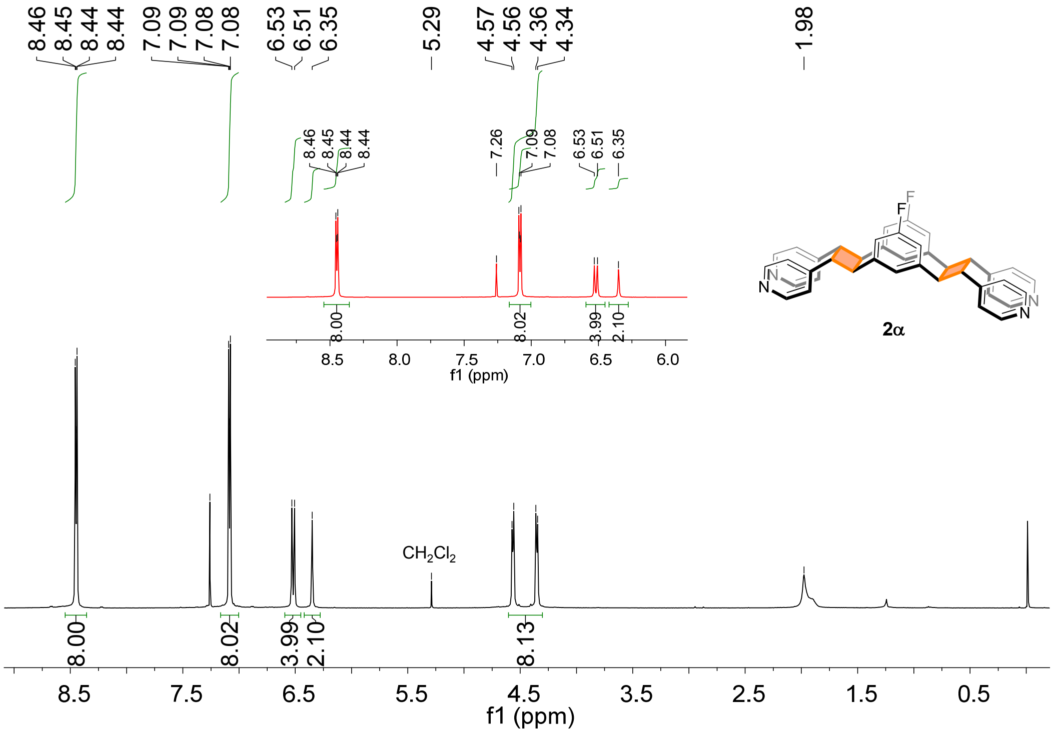
8.46
8.45
8.44
8.44
7.09
7.09
7.08
7.08
6.53
6.51
6.35
5.29
4.57
4.56
4.36
4.34
1.98
7.26
CH2Cl2
8.00
8.02
3.99
2.10
8.13
f1 (ppm)
2α

b

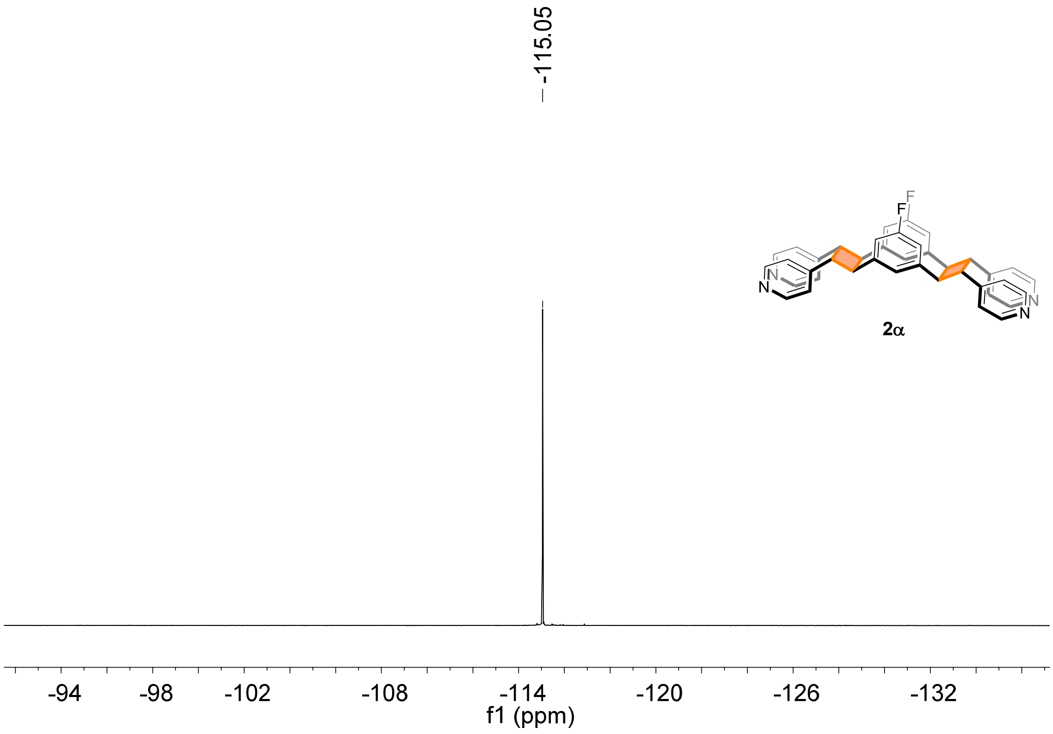
-115.05
-94
-98
-102
-108
-114
-120
-126
-132
f1 (ppm)
2α

c

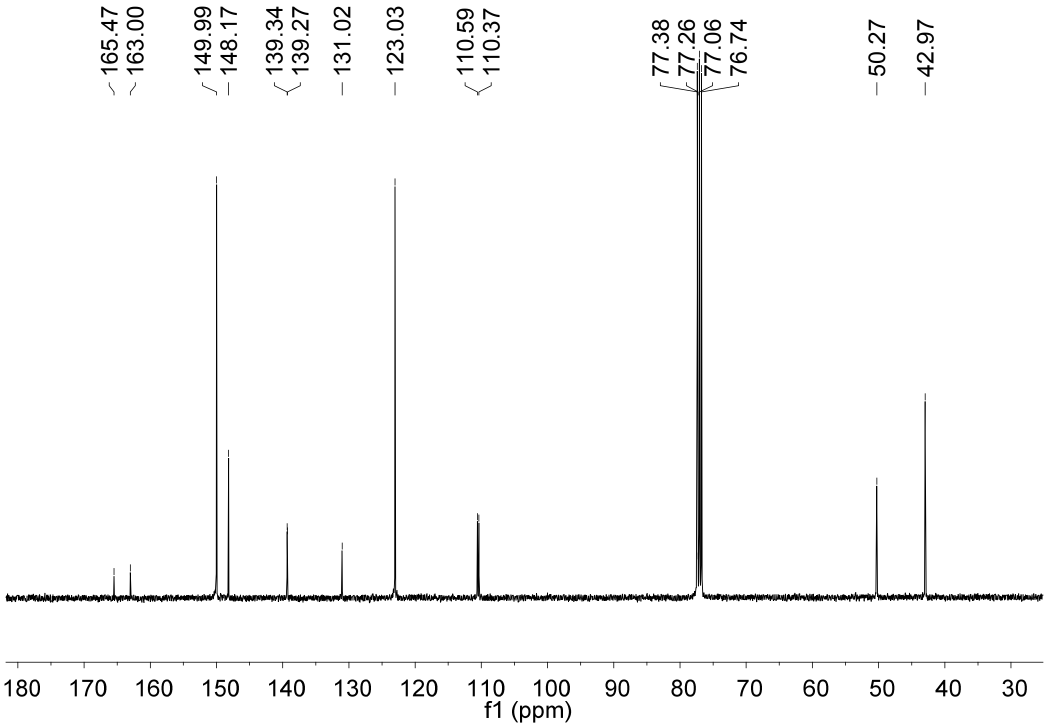
165.47
163.00
149.99
148.17
139.34
139.27
131.02
123.03
110.59
110.37
77.38
77.26
77.06
76.74
50.27
42.97
180 170 160 150 140 130 120 110 100 90 80 70 60 50 40 30
f1 (ppm)

d

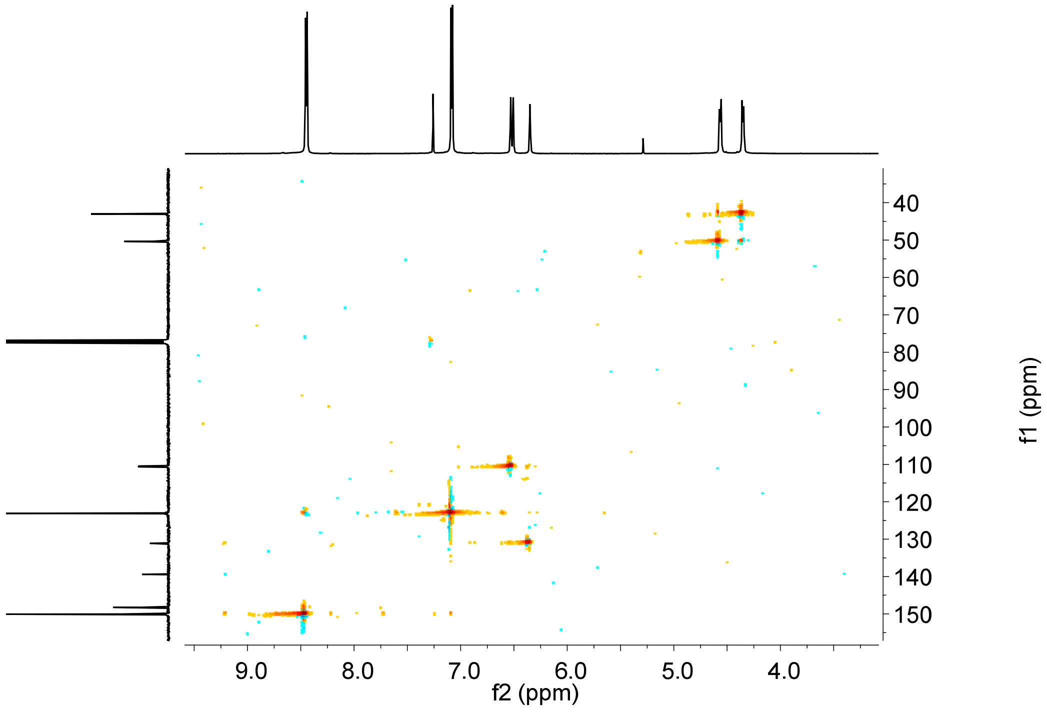
40 50 60 70 80 90 100 110 120 130 140 150
f1 (ppm)
9.0 8.0 7.0 6.0 5.0 4.0
f2 (ppm)

e

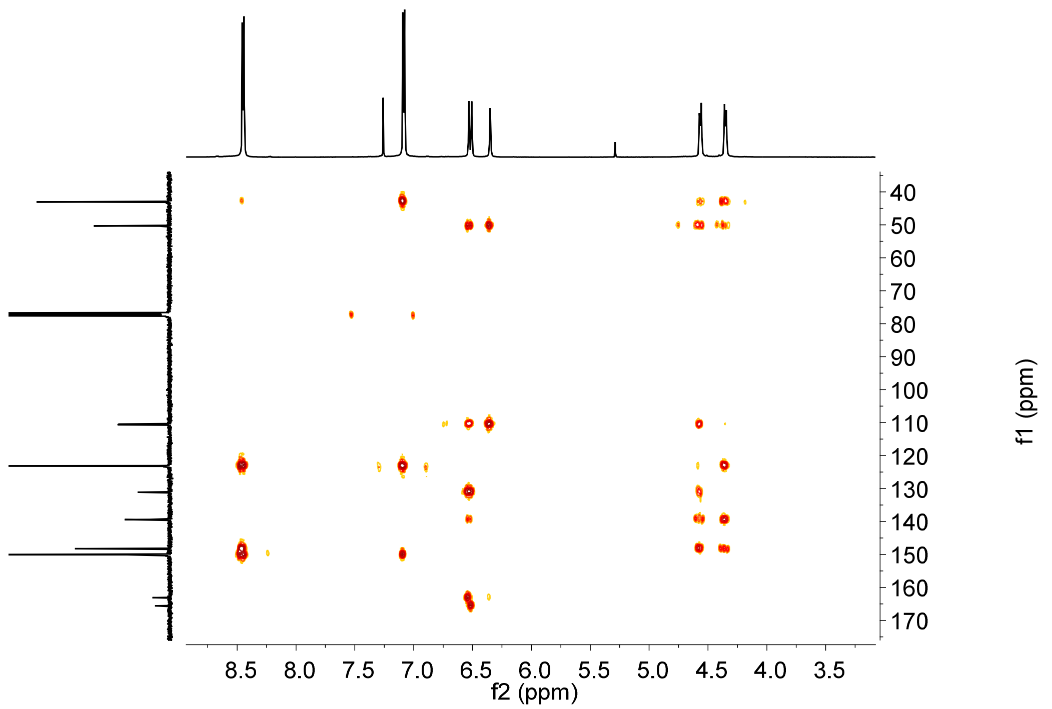

f

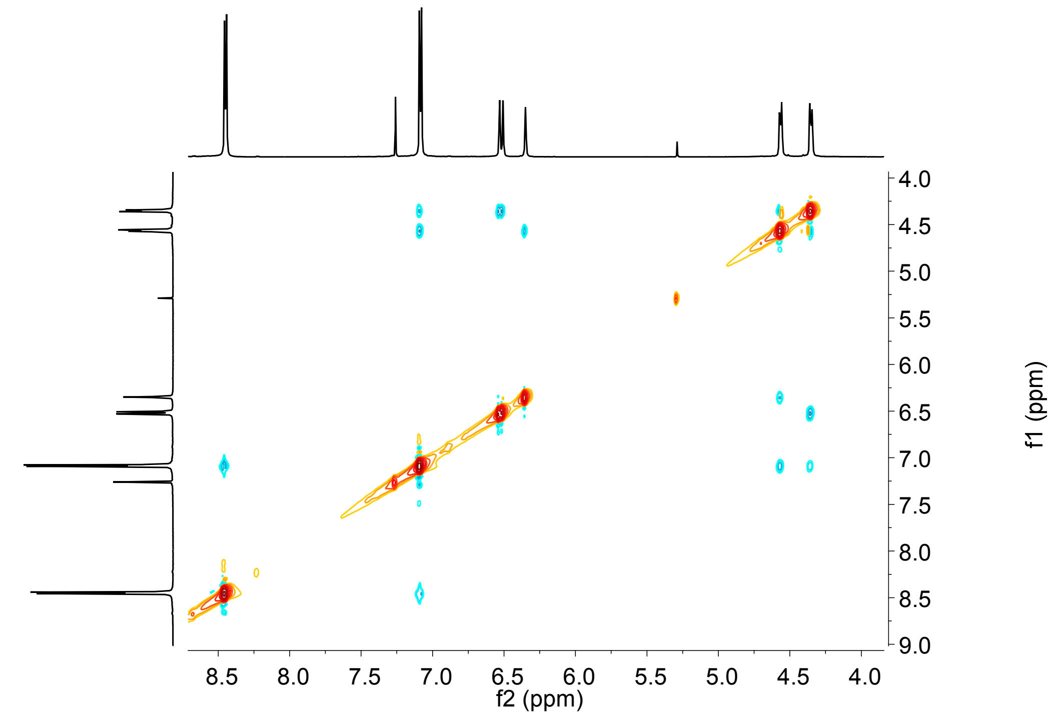

Supplementary Figure 14 | The NMR spectra of the sample isolated from decomposed **CP1-2α** in $CDCl_3$. **a** ^1H. **b** ^{19}F. **c** ^{13}C. **d** HSQC. **e** HMBC. **f** NOESY.

5. Solution NMR spectra of the transformation processes

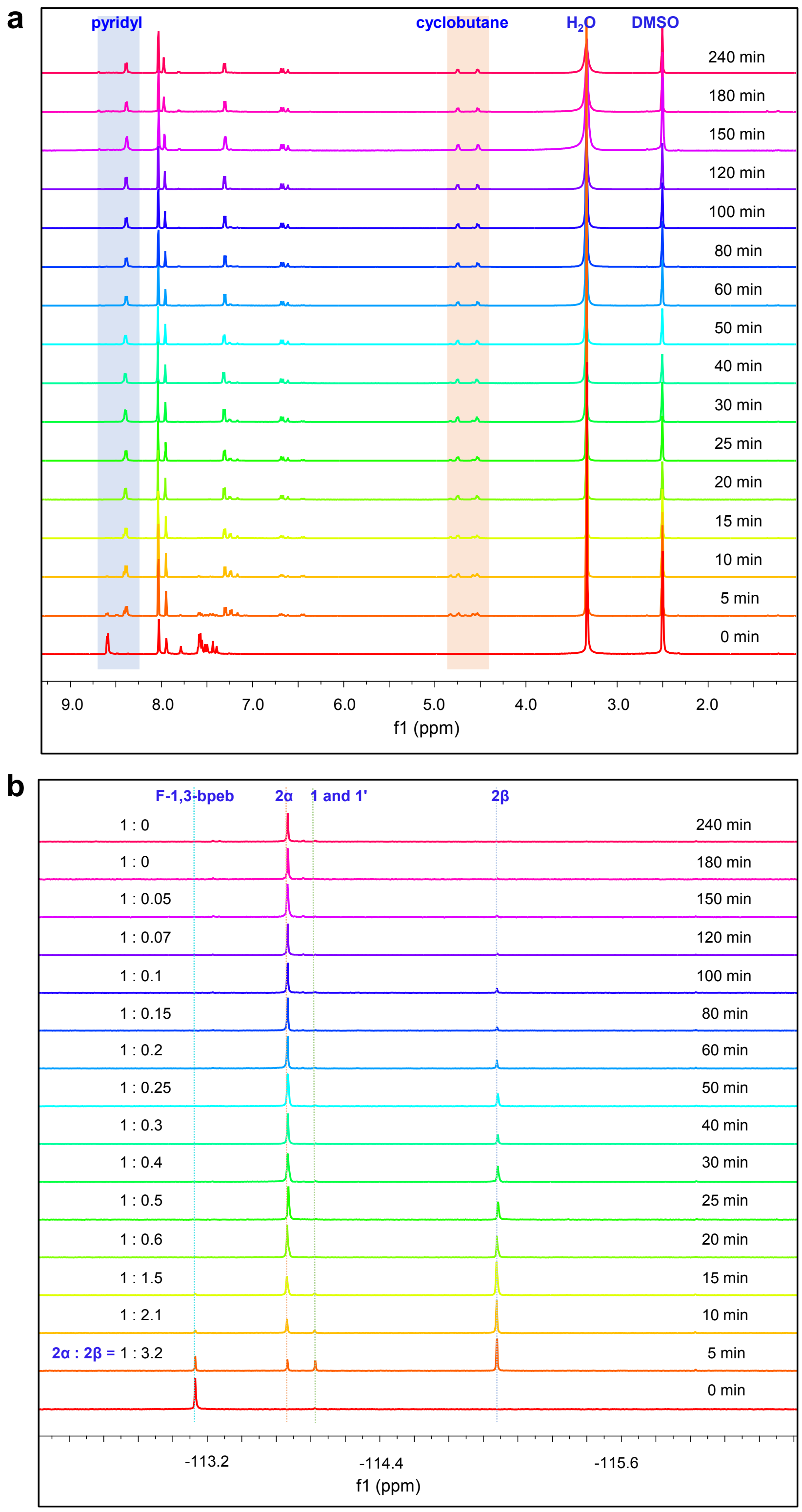

Supplementary Figure 15 | The NMR spectra of **CP1** irradiated under UV light (λ = 365 nm) at 208 °C in DMSO-d_6. **a** ^{1}H. **b** ^{19}F.

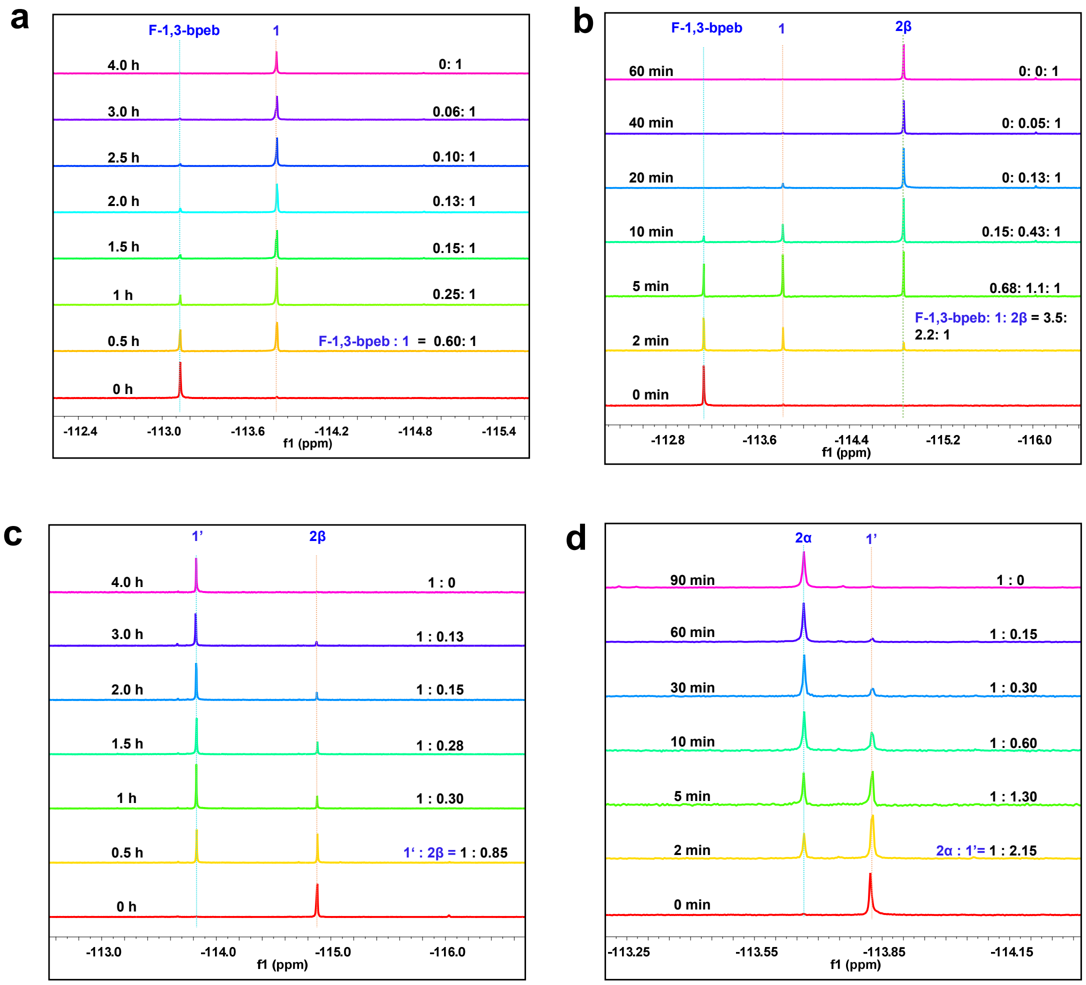

Supplementary Figure 16 | NMR characterization. The ^{19}F NMR spectra for the transformation process from (a) **CP1** to **CP1-1**; (b) **CP1** to **CP1-2β**; (c) **CP1-2β** to **CP1-1'**; (d) **CP1-1'** to **CP1-2α** in DMSO-d_6.

6. Mass spectra of pure compounds 1, 1’, 2α and 2β

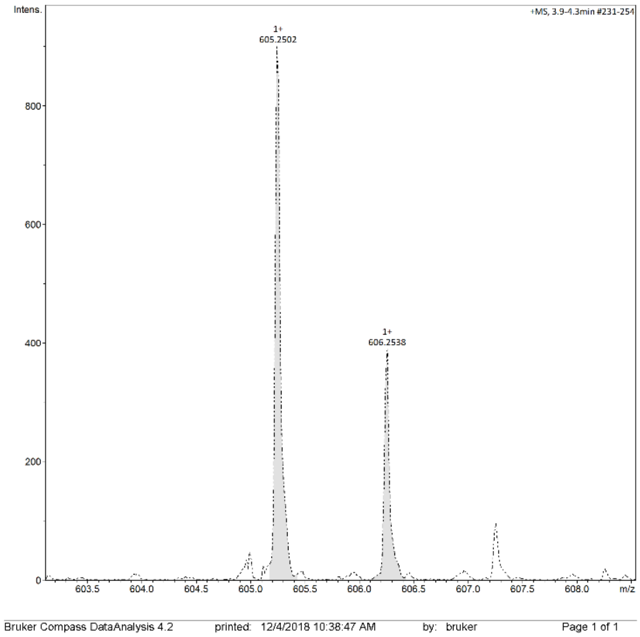

Supplementary Figure 17 | The mass spectrum of **1** in CH_3OH.

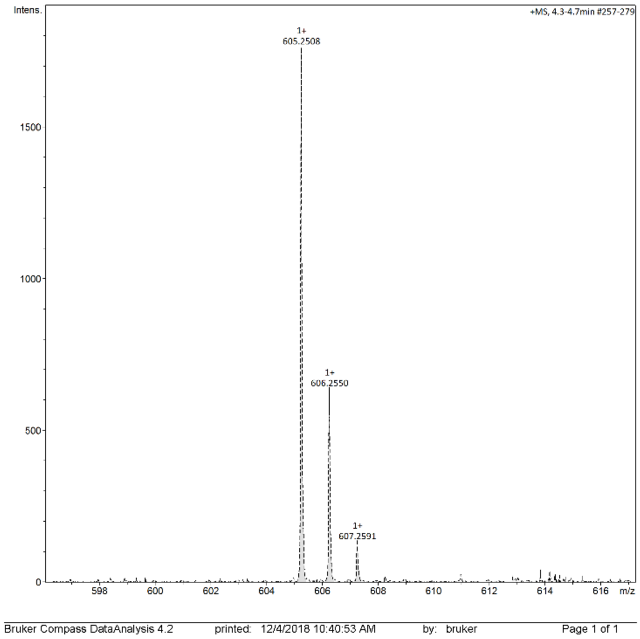

Supplementary Figure 18 | The mass spectrum of **1’** in CH_3OH.

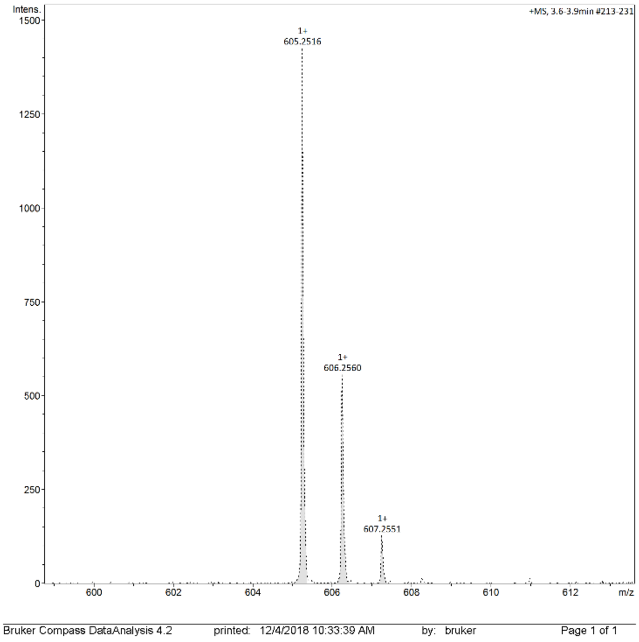

Supplementary Figure 19 | The mass spectrum of **2α** in CH_3OH.

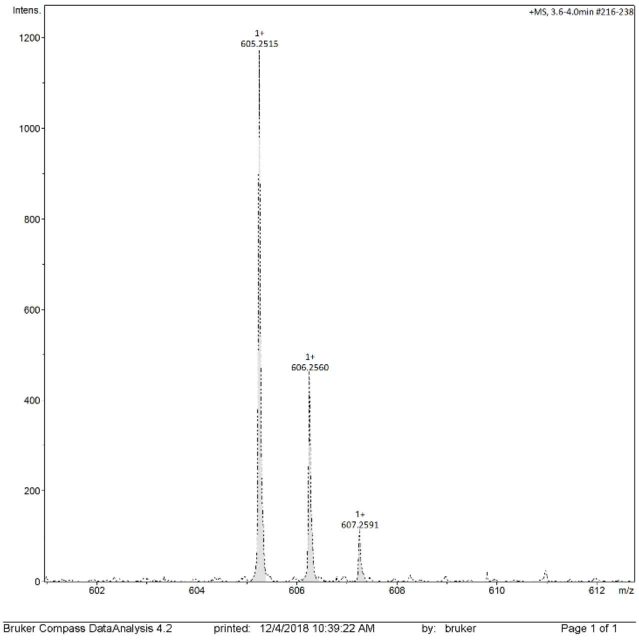

Supplementary Figure 20 | The mass spectrum of **2β** in CH_3OH.

7. Powder X-ray diffraction of CPs

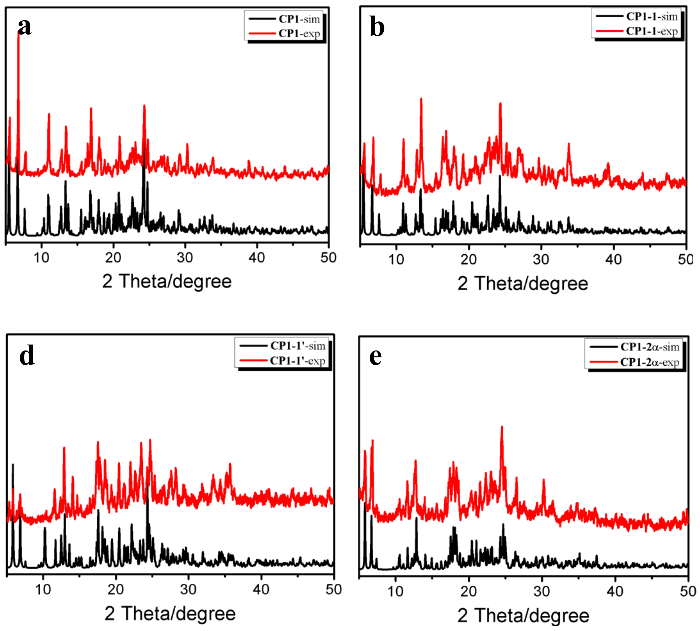

Supplementary Figure 21 | Powder X-ray diffractioncharacterization. The powder X-ray diffraction of (a) **CP1**; (b) **CP1-1**; (c) **CP1-2β**; (d) **CP1-1'**; (e) **CP1-2α**.

8. In-situ powder X-ray diffractionof the transformation processes.

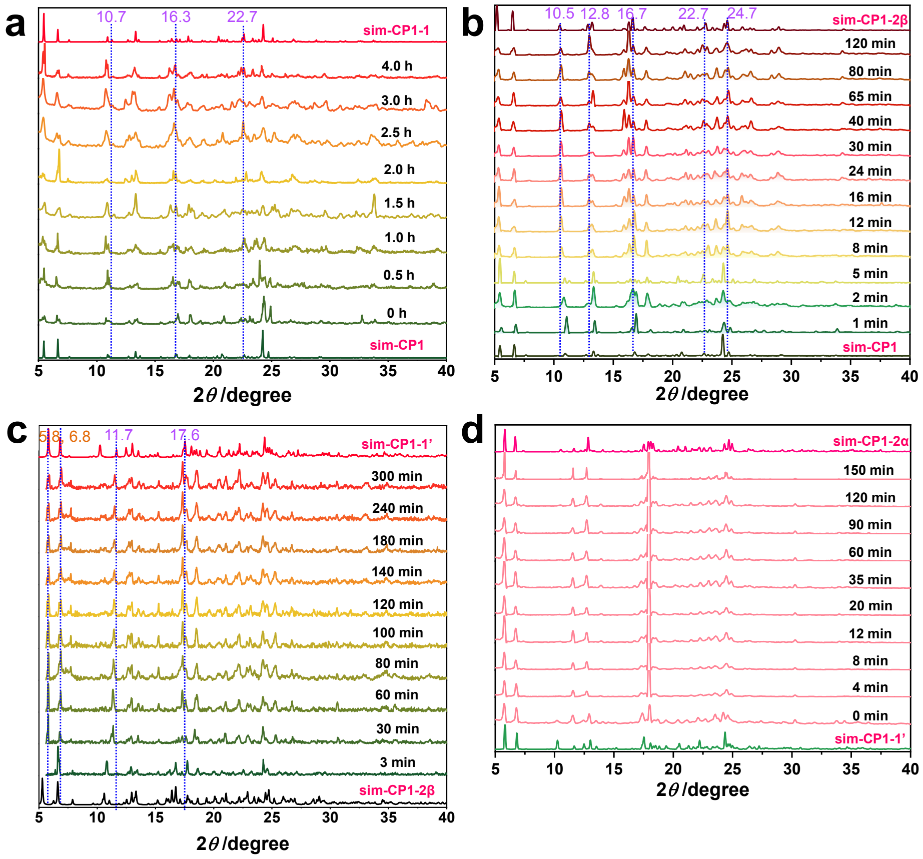

Supplementary Figure 22│In-situ powder X-ray diffraction characterization. a The powder X-ray diffraction of transformation of **CP1** to **CP1-2β** upon exposure to light at -50 ℃. **b** The powder X-ray diffraction of**CP1** to **CP1-2β** upon exposure to light at 25 ℃. **c** The powder X-ray diffraction of**CP1-2β** to **CP1-1'** at 208 ℃. **d** The powder X-ray diffraction of **CP1-1'** to **CP1-2α** upon exposure to light at 25 ℃.

9. Thermogravimetric analysis and differential scanning calorimetry analysis

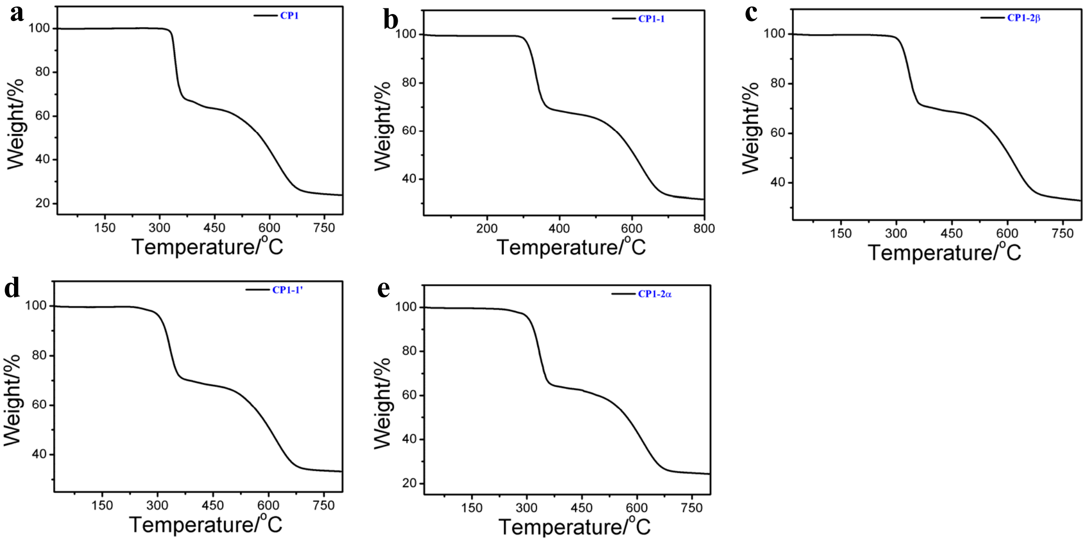

Supplementary Figure 23 | TGA characterization. a The TGA curve of **CP1**. **b** The TGA curve of **CP1-1**. **c** The TGA curve of **CP1-2β**. **d** The TGA curve of **CP1-1'**. **e** The TGA curve of **CP1-2α**.

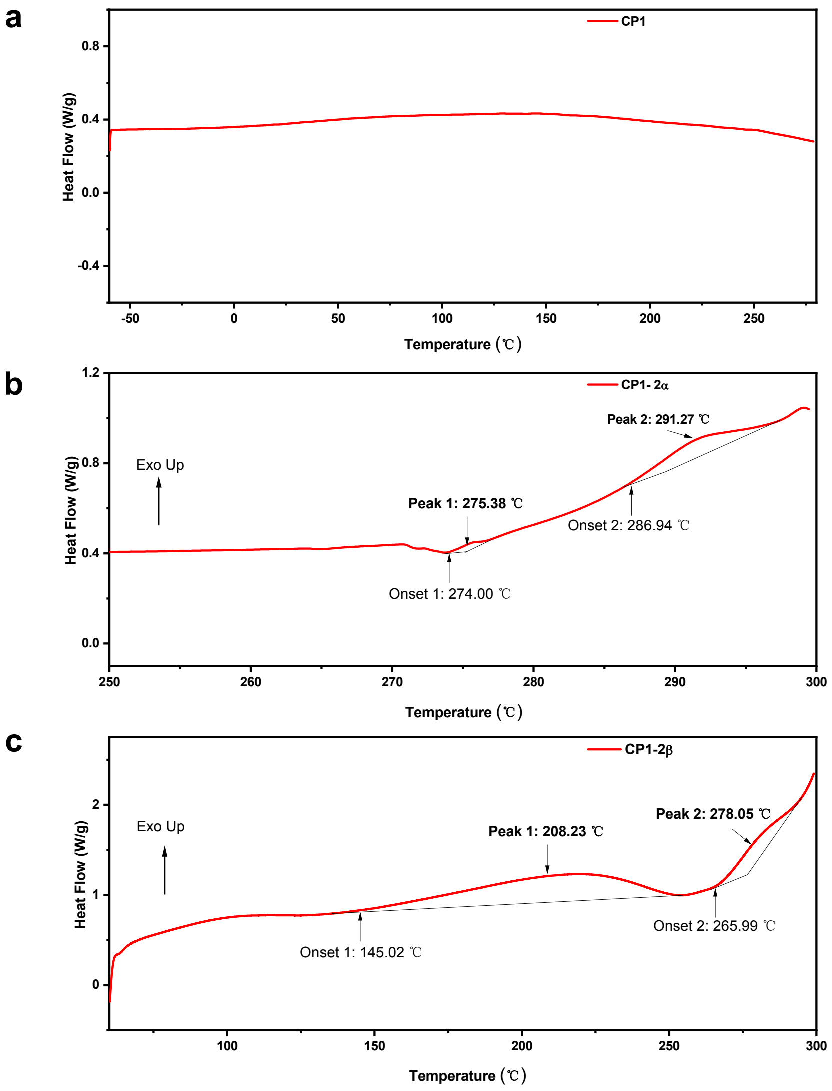

Supplementary Figure 24 | DSC characterization. The DSC thermograms of single crystals of (a) **CP1,** (b) **CP1-2α** and (c) **CP1-2β**.

10. Fourier-transform infrared (FT-IR) spectra analysis

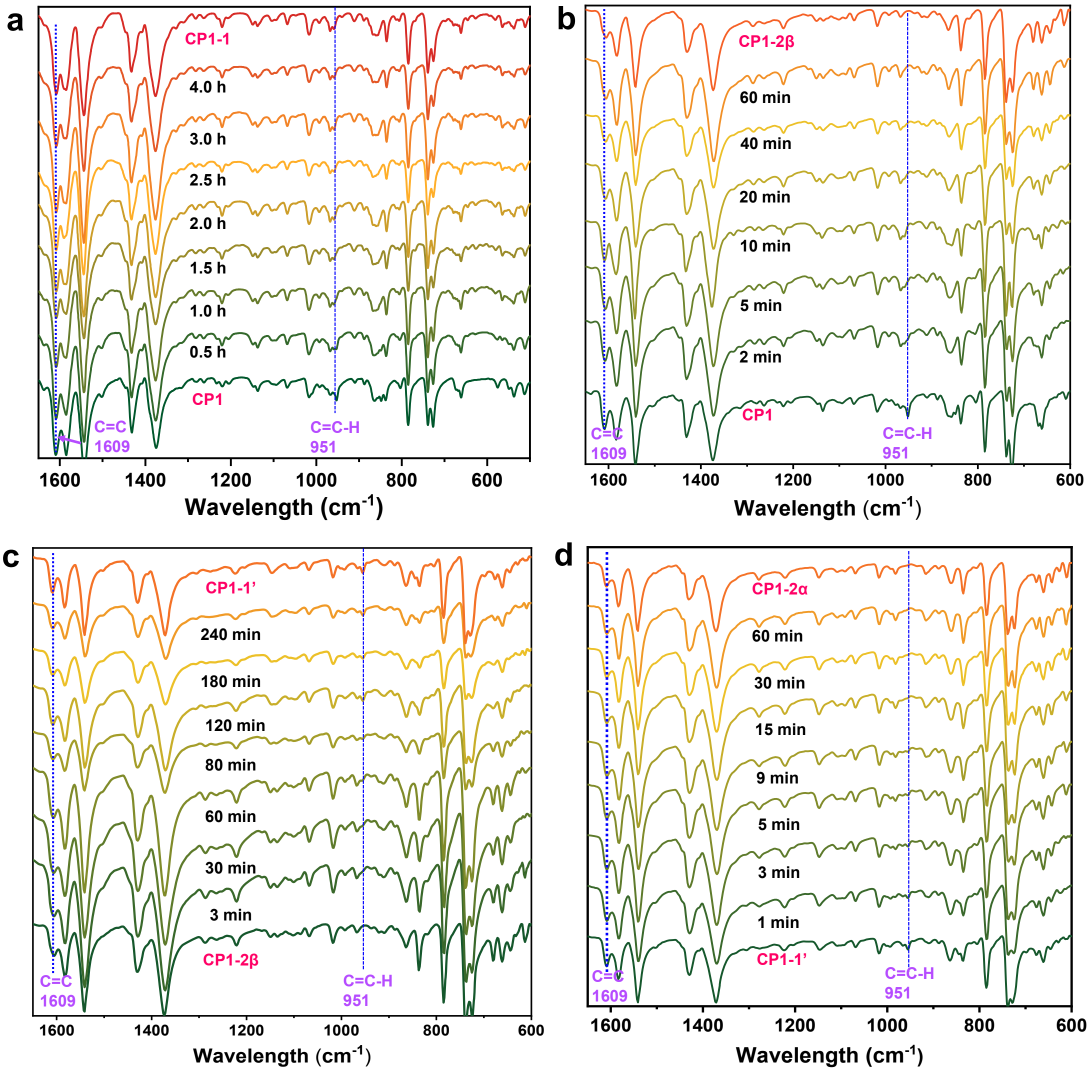

Supplementary Figure 25 | FT-IRcharacterization. a The FT-IR of transformation of **CP1** to **CP1-1** upon exposure to light at -50℃. **b** The FT-IR of **CP1** to **CP1-2β** upon exposure to light at 25 ℃. **c** The FT-IR of **CP1-2β** to **CP1-1'** at 208 ℃. **d** The FT-IR of **CP1-1'** to **CP1-2α** upon exposure to light at 25 ℃.

11. Computational methods and results.

Density functional theory (DFT) calculations were employed to explore reaction pathways of the isomerization reaction. Geometry optimization and frequency calculations were performed for the model illustrated below at the M06-2X/[Lanl2dz(Cd),6-31G*(others)] level, and single-point energy calculations were carried out at the M06-2X/def2-TZVP level. Energy values obtained at M06-2X/[Lanl2dz(Cd),6-31G*(others)] and the M06-2X/def2-TZVP levels are referred to as E1 and E2, respectively. Zero-point energy (ZPE) values also used to evaluate relative energies of different species. Gaussian 09 software was used for DFT calculations.

The following model was built from an X-ray structure of **CP1** (Supplementary Fig. 2). During DFT calculations, the Cd and O atoms in the Cd cluster (blue) were kept fixed at the positions in the X-ray structure.

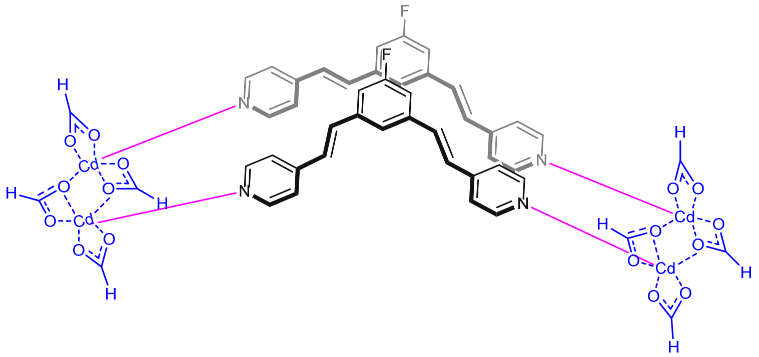

Supplementary Figure 26 | Model of **CP1** for DFT calculations. The Cd and O atoms in the Cd cluster (blue) were kept fixed.

11.1. Stability of **CP1** in the singlet ground and triplet excited states

We first examined the relative stability of several low-lying states of the **CP1**. For the triplet state, we obtained four nearly degenerate states.

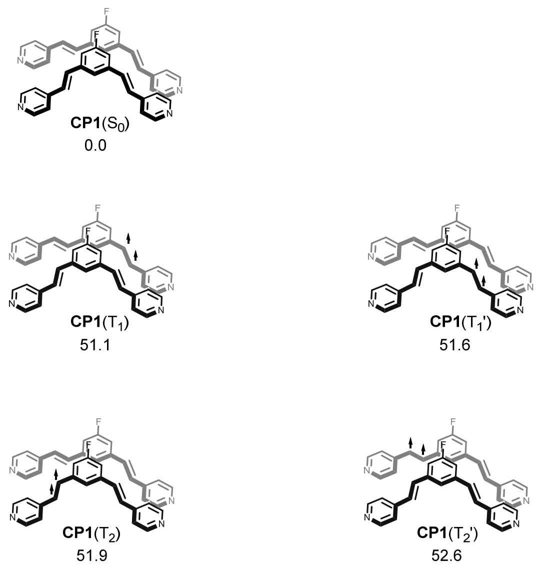

Supplementary Figure 27 | CP1 in the singlet and triplet states and relative energies (E2 + ZPE) in kcal/mol. The Cd clusters are omitted for clarity. The geometry of each state was optimized.

11.2. Comparison of three possible pathways for the transformation process from **CP1** to **CP1-1**

The energy diagram below shows that the C–C bond formation in the T_1 state has the lowest barrier. The barrier is 7.6 kcal/mol on this energy surface, which is lower than the barrier to rotation about a C–C single bond in the S_0 state (9.5 kcal/mol). The barrier in the T_2 state is somewhat higher than that in the T_1 state, suggesting that the C–C bond formation occurs more favorably in the T_1 state. The rotation process of **Int1**(S_0) leads to the **Int2**(S_0) state. However, the higher energy of **Int2**(S_0) suggests that the rotation is not a thermodynamically favorable process.

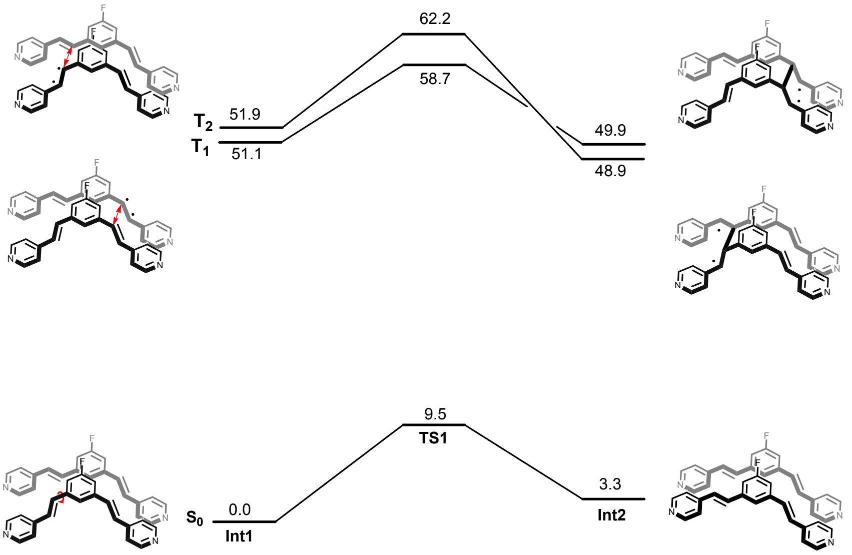

Supplementary Figure 28 | Energy profiles (in kcal/mol) for the transformation process from **CP1** to **CP1-1**.

Once **Int2**(T_1) is generated, it will be converted to an open-shell singlet state that involves a diradical and can complete the second C–C bond formation in the [2+2] cycloaddition (Supplementary Fig. 29). A single-point energy calculation suggests that the open-shell singlet analog of **Int2**(T_1) is nearly as stable as **Int2**(T_1). However, this open-shell singlet species is not characterized as a stationary point on the potential energy surface, as it undergoes C–C bond formation without an energy barrier via spin pairing.

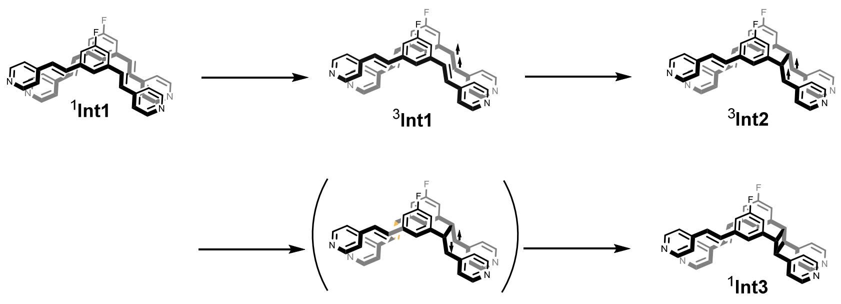

Supplementary Figure 29 | Reaction sequence in the first [2+2] cycloaddition event.

11.3. Reaction pathway in the transformation processes from **CP1** to **CP1-1**, **CP1-1** to **CP1-2β**, **CP1-2β** to **CP1-1’** and **CP1-1’** to **CP1-2α**.

Singlet excited states are typically short-lived, and our coordination polymer contains heavy cadmium ions. As such, we assumed that [2+2] photocycloaddition reactions occur in the T_1 spin state, which may be generated from a photoexcitation to a singlet excited state and a subsequent intersystem crossing event. Indeed, the triplet spin state has been invoked in the majority of known [2+2] photocycloaddition reactions[4]. It should be noted that there are also cases where the singlet manifold is preferentially used for [2+2] photocycloaddition, especially when heavy elements are not involved. Which excited-state pathway is chosen during cycloaddition sometimes has a critical impact on product selectivity. For example, Bach and coworkers have experimentally shown that [2+2] photocycloaddition reactions use different spin states and exhibit

different enantioselectivity depending on the reaction condition[5,6], and Chen, Dolg, and coworkers have performed *ab initio* theoretical studies to rationalize the experimental results[7,8]. However, the geometric features and constraints in our coordination polymer preclude the spin-dependent induction of enantioselectivity.

Our DFT model in Supplementary Fig.26 is based on the X-ray structure of **CP1**, and the Cd and O atoms are held fixed throughout all geometry optimization calculations. We also performed additional calculations on those species for which X-ray structures have been determined, using the same truncated model shown in Supplementary Fig. 26, but by building the model from the respective X-ray structures. Such calculations were performed for **Int3** (**CP1-1**) and **Int6** (**CP1-2β**). Their relative energies calculated in this way (shown by thick blue dashed lines in Fig.4) are somewhat lower than the energies obtained using the original **CP1**-based model. This result suggests that the Cd ions in the crystal structure are not rigidly fixed throughout the reaction but undergo some structural change to adjust to new coordination environments. Indeed, close inspection of X-ray structures (Supplementary Figs. 31 and 32 and Supplementary Table 3) shows that the internuclear distances between Cd ions change, which is more significant in later stages. The energy drop by changing the model tends to be larger when the geometric change is larger. Therefore, the energy diagram obtained using the **CP1**-based model would be somewhat biased toward the endothermic direction.

X-ray structures of **Int10** (**CP1-1'**) and **Int12** (**CP1-2α**) are available. We performed calculations on these species by rebuilding the model from their X-ray structures. The result shows that the energy drops for these species are larger than those observed for **Int3** and **Int5**. This can be understood from the fact that the movement of the Cd ions in the crystal structure from their positions in the crystal

structure of **CP1** is more significant in these species (Supplementary Table 3). Thus, the relative energy of **Int10** is likely be overestimated in the **CP1**-based model.

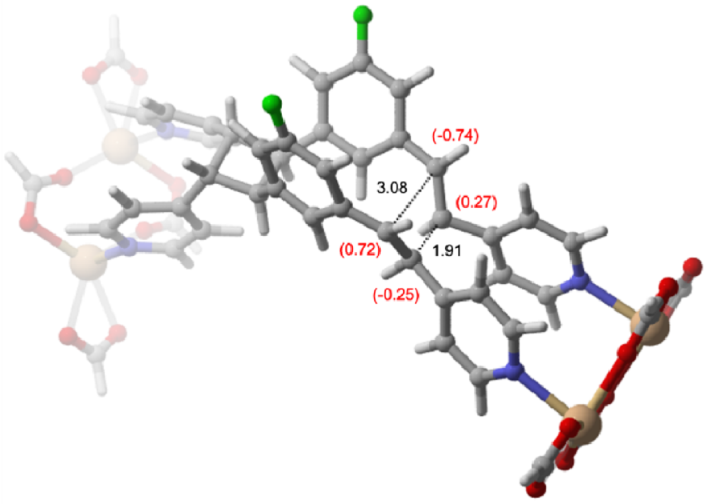

Supplementary Figure 30 | Optimized structure of [1]**TS4**. Key bond distances are shown in Å, and B2-calculated key spin density values are presented in parentheses.

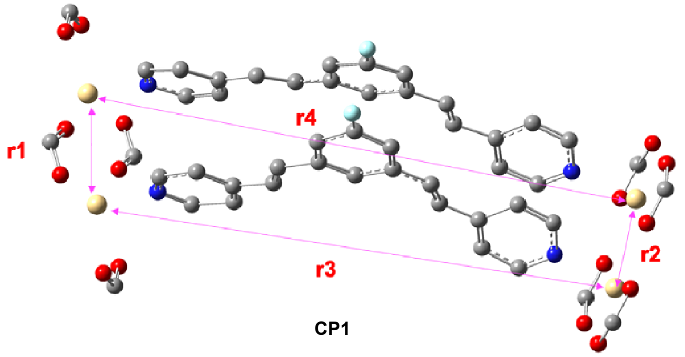

Supplementary Figure 31 | Heavy atoms in the X-ray structure of **CP1**, with only the atoms in the calculation model shown.

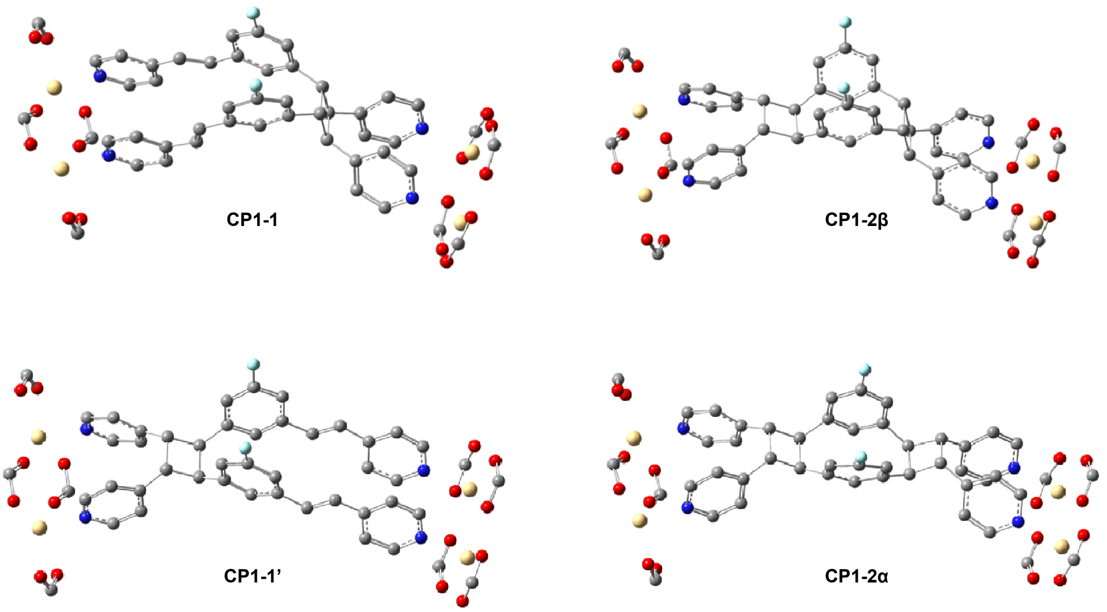

Supplementary Figure 32 | Heavy atoms in the X-ray structures of **CP1-1**, **CP1-2β**, **CP1-1'**, and **CP1-2α**, with only the atoms in the calculation model shown.

Supplementary Table 3. Cd–Cd distances (in Å) defined in Supplementary Fig.33 and deviations of the distances (in Å) with respect to the corresponding distances for **CP1**.

**Cd–Cd distances (in Å) defined in Supplementary Fig.33**				
	r1	r2	r3	r4
**CP1**	3.97	4.02	17.09	18.08
**CP1-1**	3.96	4.23	16.97	17.61
**CP1-2β**	4.24	4.15	17.49	17.69
**CP1-1'**	4.28	3.85	18.50	18.68
**CP1-2α**	4.27	4.11	18.45	18.70
**Deviations of the distances (in Å) with respect to the corresponding distances for CP1**				
	r1	r2	r3	r4
**CP1**	0.00	0.00	0.00	0.00
**CP1-1**	-0.01	0.21	-0.12	-0.47
**CP1-2β**	0.27	0.13	0.40	-0.39
**CP1-1'**	0.31	-0.17	1.41	0.60
**CP1-2α**	0.30	0.09	1.36	0.62

11.4. XYZ coordinates of optimized geometries

=== ^{1}**Int1 (CP1)** ===

Cd	19.415000	24.466000	24.406000
Cd	16.370000	24.133000	21.810000
Cd	29.590000	17.101000	12.821000
Cd	27.131000	15.292000	10.287000
O	30.396000	14.953000	13.259000
O	26.325000	17.440000	9.849000
O	28.931000	14.769000	11.743000
O	27.789000	17.624000	11.364000
O	29.974000	19.426000	13.017000
O	26.747000	12.968000	10.091000
O	31.101000	18.203000	14.445000
O	25.620000	14.190000	8.663000
O	19.734000	23.407000	26.461000
O	16.050000	25.192000	19.755000
O	21.353000	24.735000	25.823000
O	14.431000	23.864000	20.393000
O	20.336000	25.881000	22.872000
O	15.448000	22.718000	23.344000
O	17.349000	23.397000	24.245000
O	18.436000	25.202000	21.971000
C	17.168849	21.064129	21.109476
C	17.846762	19.968077	20.598319
C	19.073137	20.161953	19.950067
C	19.533784	21.476501	19.821737
C	18.791481	22.516588	20.359149
C	19.888575	19.057448	19.433019
C	19.801457	17.786022	19.848426
C	20.637330	16.678403	19.367053
C	20.799805	15.556426	20.182696
C	21.624575	14.526503	19.755264
C	22.272186	14.550765	18.535694
C	22.094119	15.658290	17.692634
C	21.280895	16.710345	18.123031
C	22.721457	15.744843	16.366269
C	23.639830	14.896785	15.881712
C	24.304399	14.995675	14.579157
C	25.414809	14.181769	14.333355
C	26.107230	14.305975	13.138666
C	24.685108	15.961760	12.409237
C	23.924908	15.887787	13.566154
C	26.870671	17.415618	14.600613
C	26.064303	17.403831	15.729834
C	26.445524	16.656632	16.850023
C	27.657360	15.957211	16.766719
C	28.408642	16.026942	15.604008
C	25.584687	16.646602	18.039713
C	25.701406	15.803559	19.075916
C	24.857283	15.808129	20.279376
C	24.109319	16.925834	20.663853
C	23.303795	16.895163	21.805809
C	23.236066	15.718554	22.563707
C	23.985501	14.621237	22.176649
C	24.802027	14.646004	21.059020
C	22.501028	18.048454	22.236775
C	22.681255	19.322955	21.860195
C	21.863271	20.452218	22.322062
C	22.352732	21.758515	22.207007
C	21.599936	22.821050	22.689413
C	19.908698	21.407587	23.352456
C	20.593156	20.293696	22.894081
C	29.740983	14.231467	12.511494
C	26.953122	18.158393	10.622381
C	30.761617	19.304462	13.983913
C	25.902305	13.094183	9.175988
C	19.505688	25.860570	21.937109
C	16.300113	22.704480	24.259473
C	20.866322	23.916897	26.623355
C	14.947544	24.629725	19.560667
N	25.763816	15.195292	12.203127
N	17.635642	22.315126	21.000738
N	28.022345	16.741180	14.536447
N	20.399875	22.649291	23.255372
F	21.791805	13.465575	20.554552
F	23.918020	13.502528	22.903329
H	16.215839	20.958029	21.621691
H	17.409340	18.979768	20.688808
H	20.475432	21.685138	19.323762
H	19.119879	23.549084	20.292066
H	20.652891	19.334200	18.708632
H	19.097137	17.530913	20.639826
H	20.311572	15.478544	21.149220
H	22.880617	13.698325	18.256602
H	21.114686	17.553127	17.457460
H	22.404037	16.593810	15.761818
H	23.989174	14.076208	16.505210
H	25.747038	13.463831	15.076655
H	26.965847	13.683626	12.906236
H	24.438474	16.653432	11.607434
H	23.051083	16.522377	13.668291
H	26.602682	17.980540	13.711726
H	28.038170	15.386933	17.606601
H	29.360238	15.510723	15.506678
H	24.775895	17.375954	18.034130
H	26.460452	15.023040	19.047305
H	24.151838	17.828925	20.063778
H	22.617795	15.653497	23.453413
H	25.367694	13.758514	20.794325
H	21.715762	17.818756	22.956294
H	23.515031	19.582075	21.210358
H	23.328677	21.944379	21.769553
H	21.958791	23.846085	22.639178
H	18.928069	21.323388	23.812807
H	20.130658	19.315351	22.971458
H	25.134585	17.965261	15.734972
H	16.126230	22.042628	25.123288
H	21.451418	23.619840	27.510537
H	14.423365	24.822648	18.608888
H	19.731332	26.441479	21.028303
H	25.385223	12.187537	8.815507
H	26.770520	19.242705	10.667932
H	29.861107	13.137595	12.526209
H	31.163285	20.225378	14.442035

=== ^{3}**Int1** ===

Cd	19.415000	24.466000	24.406000
Cd	16.370000	24.133000	21.810000
Cd	29.590000	17.101000	12.821000
Cd	27.131000	15.292000	10.287000
O	30.396000	14.953000	13.259000
O	26.325000	17.440000	9.849000
O	28.931000	14.769000	11.743000
O	27.789000	17.624000	11.364000
O	29.974000	19.426000	13.017000
O	26.747000	12.968000	10.091000
O	31.101000	18.203000	14.445000
O	25.620000	14.190000	8.663000
O	19.734000	23.407000	26.461000
O	16.050000	25.192000	19.755000
O	21.353000	24.735000	25.823000
O	14.431000	23.864000	20.393000
O	20.336000	25.881000	22.872000
O	15.448000	22.718000	23.344000
O	17.349000	23.397000	24.245000
O	18.436000	25.202000	21.971000
C	17.311961	21.048381	21.130655
C	18.075742	19.986502	20.682615
C	19.311862	20.238783	20.030051
C	19.657422	21.602262	19.842711
C	18.830772	22.592389	20.329419
C	20.212982	19.218043	19.669317
C	20.068787	17.812145	20.079250
C	20.608264	16.714058	19.397148
C	20.613654	15.443297	20.037790

C	21.249623	14.386472	19.433937
C	21.856854	14.488308	18.187999
C	21.841515	15.724728	17.515469
C	21.227696	16.819116	18.116702
C	22.488679	15.872793	16.201227
C	23.405564	15.017571	15.726068
C	24.132523	15.107606	14.458012
C	25.244105	14.277542	14.279384
C	25.997179	14.378208	13.120710
C	24.632036	16.042525	12.300713
C	23.816123	15.995263	13.421373
C	26.836066	17.450301	14.545972
C	25.979424	17.426293	15.637408
C	26.286980	16.632242	16.748100
C	27.482230	15.901919	16.695167
C	28.287588	15.986381	15.568665
C	25.377154	16.613311	17.902996
C	25.386734	15.696498	18.881945
C	24.518716	15.694371	20.070315
C	23.875090	16.847561	20.530511
C	23.089066	16.824368	21.688264
C	22.929941	15.615731	22.379564
C	23.564066	14.477785	21.910570
C	24.362462	14.495809	20.779788
C	22.388290	18.009406	22.196724
C	22.595161	19.287144	21.832961
C	21.833325	20.431463	22.347376
C	22.318619	21.732756	22.172502
C	21.590881	22.810091	22.661293
C	19.933415	21.419530	23.446716
C	20.599228	20.292191	22.998021
C	29.738978	14.231018	12.513423
C	26.953213	18.158417	10.622327
C	30.763379	19.304308	13.982657
C	25.902791	13.094149	9.175403
C	19.504695	25.861869	21.937948
C	16.299429	22.704933	24.259457
C	20.867225	23.915989	26.622442
C	14.948009	24.628545	19.560292
N	25.707460	15.257037	12.157414
N	17.682839	22.331548	20.980733
N	27.971287	16.744783	14.508698
N	20.418013	22.656647	23.284354
F	21.299245	13.209801	20.070499
F	23.404514	13.328816	22.573002
H	16.366100	20.890172	21.642455
H	17.726407	18.970865	20.834296
H	20.584180	21.866937	19.343316
H	19.077419	23.643734	20.221130
H	21.144134	19.513638	19.189383
H	19.623298	17.626883	21.054449
H	20.158505	15.308407	21.013281
H	22.300292	13.600006	17.754690
H	21.210840	17.773162	17.596605
H	22.208869	16.754530	15.625810
H	23.713867	14.184495	16.354201
H	25.527260	13.564628	15.047414
H	26.861452	13.745291	12.940308
H	24.434045	16.724968	11.477974
H	22.946227	16.641266	13.477072
H	26.624017	18.051387	13.665883
H	27.809178	15.297765	17.534291
H	29.228136	15.445870	15.498136
H	24.626149	17.402508	17.917010
H	26.081346	14.859852	18.819031
H	24.002301	17.779040	19.988277
H	22.326802	15.555174	23.280070
H	24.844003	13.578631	20.455708
H	21.646106	17.801551	22.966619
H	23.392552	19.530221	21.132606
H	23.269286	21.904849	21.677421
H	21.945524	23.833107	22.563526
H	18.975163	21.351098	23.954758
H	20.140320	19.318604	23.134722
H	25.067435	18.015806	15.619481
H	16.124643	22.044249	25.124066
H	21.453432	23.616472	27.508165
H	14.424864	24.820098	18.607635
H	19.729273	26.444837	21.030037
H	25.387040	12.187173	8.813862
H	26.769838	19.242673	10.668943
H	29.856663	13.137071	12.530226
H	31.168091	20.225118	14.438218

=== [1]**TS1** ===

Cd	19.415000	24.466000	24.406000
Cd	16.370000	24.133000	21.810000
Cd	29.590000	17.101000	12.821000
Cd	27.131000	15.292000	10.287000
O	30.396000	14.953000	13.259000
O	26.325000	17.440000	9.849000
O	28.931000	14.769000	11.743000
O	27.789000	17.624000	11.364000
O	29.974000	19.426000	13.017000
O	26.747000	12.968000	10.091000
O	31.101000	18.203000	14.445000
O	25.620000	14.190000	8.663000
O	19.734000	23.407000	26.461000
O	16.050000	25.192000	19.755000
O	21.353000	24.735000	25.823000
O	14.431000	23.864000	20.393000
O	20.336000	25.881000	22.872000
O	15.448000	22.718000	23.344000
O	17.349000	23.397000	24.245000
O	18.436000	25.202000	21.971000
C	17.290940	21.024159	21.181925
C	18.056850	19.960651	20.735120
C	19.250980	20.213071	20.026508
C	19.548713	21.564222	19.755496
C	18.733020	22.562880	20.254511
C	20.170439	19.193198	19.608495
C	20.293731	17.886690	20.199664
C	20.627036	16.723380	19.326641
C	20.362643	15.445437	19.826020
C	20.763685	14.335315	19.103384
C	21.409583	14.440178	17.885268
C	21.660235	15.713964	17.361958
C	21.249887	16.844074	18.079901
C	22.374955	15.871043	16.084575
C	23.252343	14.970180	15.619515
C	24.038633	15.072216	14.387537
C	25.168833	14.259672	14.250540
C	25.964565	14.377695	13.122126
C	24.605507	16.023300	12.254199
C	23.749085	15.959439	13.343303
C	26.812155	17.427240	14.495802
C	25.929919	17.385950	15.565261
C	26.226254	16.601024	16.685519
C	27.434537	15.890736	16.661600
C	28.264320	15.989396	15.554847
C	25.280224	16.571345	17.808734
C	25.348846	15.763599	18.876781
C	24.402432	15.760881	20.002317
C	23.553121	16.840151	20.269100
C	22.635628	16.798697	21.319512
C	22.586288	15.667817	22.139396
C	23.453000	14.616012	21.887450
C	24.354392	14.637761	20.837236
C	21.687849	17.926241	21.560862
C	22.290499	19.230813	21.648954
C	21.667641	20.367245	22.253568
C	22.246591	21.650519	22.147089
C	21.585677	22.748432	22.670305
C	19.855738	21.431914	23.440672
C	20.443227	20.283148	22.948718
C	29.737894	14.230923	12.514564
C	26.954331	18.158640	10.621389
C	30.763206	19.304301	13.982760
C	25.903125	13.094010	9.175337
C	19.508820	25.855682	21.935177

C	16.296655	22.708973	24.262146
C	20.867633	23.915313	26.621878
C	14.947900	24.628048	19.560149
N	25.696926	15.254169	12.149526
N	17.630197	22.305494	20.972049
N	27.960076	16.742217	14.487266
N	20.401725	22.646252	23.292147
F	20.510816	13.120136	19.599487
F	23.411694	13.541994	22.682938
H	16.370412	20.867022	21.738491
H	17.724455	18.947279	20.933593
H	20.443091	21.826659	19.199287
H	18.957779	23.613257	20.096343
H	20.942555	19.512362	18.911670
H	19.464098	17.644157	20.864644
H	19.870689	15.301675	20.783054
H	21.669836	13.537105	17.345347
H	21.442092	17.825954	17.656082
H	22.196627	16.797283	15.538574
H	23.475972	14.098421	16.231963
H	25.432350	13.548311	15.026941
H	26.846570	13.760899	12.974736
H	24.426089	16.703275	11.425096
H	22.863549	16.585809	13.366477
H	26.610059	18.021896	13.609214
H	27.748566	15.281866	17.502084
H	29.213349	15.461658	15.504871
H	24.444416	17.263765	17.719828
H	26.143059	15.021521	18.940045
H	23.597755	17.724388	19.639603
H	21.893236	15.596199	22.971737
H	25.003305	13.783270	20.675152
H	21.022635	17.706434	22.396591
H	23.248735	19.391262	21.161746
H	23.200478	21.781255	21.645798
H	21.995131	23.752648	22.597663
H	18.909490	21.403488	23.973626
H	19.938176	19.334158	23.090235
H	25.007437	17.957771	15.524639
H	16.117656	22.054926	25.130948
H	21.454498	23.614401	27.506717
H	14.425366	24.819077	18.607070
H	19.738861	26.428803	21.022403
H	25.387517	12.187072	8.813411
H	26.772446	19.243220	10.666350
H	29.853710	13.136801	12.533121
H	31.167664	20.225127	14.438547

=== ^{3}**TS1** ===

Cd	19.415000	24.466000	24.406000
Cd	16.370000	24.133000	21.810000
Cd	29.590000	17.101000	12.821000
Cd	27.131000	15.292000	10.287000
O	30.396000	14.953000	13.259000
O	26.325000	17.440000	9.849000
O	28.931000	14.769000	11.743000
O	27.789000	17.624000	11.364000
O	29.974000	19.426000	13.017000
O	26.747000	12.968000	10.091000
O	31.101000	18.203000	14.445000
O	25.620000	14.190000	8.663000
O	19.734000	23.407000	26.461000
O	16.050000	25.192000	19.755000
O	21.353000	24.735000	25.823000
O	14.431000	23.864000	20.393000
O	20.336000	25.881000	22.872000
O	15.448000	22.718000	23.344000
O	17.349000	23.397000	24.245000
O	18.436000	25.202000	21.971000
C	17.296013	21.035274	21.209358
C	18.042847	19.961122	20.759050
C	19.225134	20.192605	20.013884
C	19.536457	21.546662	19.739735
C	18.740116	22.553348	20.246378
C	20.103671	19.161261	19.588290
C	20.050381	17.792368	20.042528
C	20.460982	16.668188	19.241911
C	20.264243	15.375088	19.765786
C	20.757270	14.282228	19.083923
C	21.419092	14.398500	17.872404
C	21.605423	15.674422	17.319984
C	21.127542	16.793846	18.004376
C	22.324244	15.838979	16.045860
C	23.223536	14.955119	15.591026
C	24.016868	15.064314	14.364951
C	25.155450	14.261840	14.239136
C	25.959014	14.384224	13.117124
C	24.592372	16.016553	12.235000
C	23.727827	15.947448	13.317205
C	26.800611	17.443301	14.479274
C	25.903895	17.403387	15.536954
C	26.179336	16.609331	16.656019
C	27.384982	15.894516	16.646764
C	28.230465	15.992903	15.551157
C	25.217101	16.573633	17.766429
C	25.212720	15.686398	18.771982
C	24.252611	15.666909	19.887157
C	23.502919	16.790845	20.247232
C	22.582653	16.743216	21.302433
C	22.417115	15.539916	22.004931
C	23.176537	14.438662	21.650639
C	24.089331	14.477318	20.609552
C	21.752055	17.889714	21.669249
C	22.171291	19.213492	21.528256
C	21.553192	20.352317	22.154707
C	22.086718	21.642083	21.958583
C	21.465447	22.740386	22.528316
C	19.845992	21.413615	23.488825
C	20.401448	20.263107	22.962080
C	29.739169	14.231003	12.513415
C	26.954464	18.158666	10.621151
C	30.763656	19.304305	13.982433
C	25.903175	13.093988	9.175288
C	19.507242	25.857384	21.935863
C	16.298413	22.707554	24.261068
C	20.868034	23.915275	26.621491
C	14.947957	24.628127	19.560223
N	25.690908	15.256241	12.140870
N	17.642511	22.311905	20.982892
N	27.944567	16.750829	14.482344
N	20.356100	22.633448	23.272610
F	20.591842	13.065144	19.612553
F	23.020655	13.299693	22.332265
H	16.384146	20.890800	21.783506
H	17.700268	18.954353	20.970677
H	20.420537	21.797207	19.161794
H	18.971457	23.600630	20.077798
H	20.907497	19.447499	18.914907
H	19.328537	17.565236	20.822406
H	19.750604	15.224184	20.709822
H	21.740796	13.498413	17.361642
H	21.265733	17.776175	17.561626
H	22.125676	16.756236	15.492129
H	23.463946	14.092539	16.209742
H	25.418618	13.554213	15.019074
H	26.847686	13.775088	12.978274
H	24.414349	16.693481	11.403123
H	22.837353	16.566958	13.332496
H	26.614472	18.043346	13.592819
H	27.686074	15.287129	17.493197
H	29.178010	15.461515	15.512925
H	24.428709	17.323576	17.715329
H	25.950545	14.885390	18.782395
H	23.639832	17.717903	19.699196
H	21.717235	15.458109	22.830599
H	24.657875	13.585367	20.367557
H	21.015844	17.680417	22.441540
H	23.054422	19.421229	20.928354
H	22.983071	21.779288	21.361149
H	21.851086	23.748274	22.397948

H	18.954336	21.381066	24.108473
H	19.923888	19.310953	23.165373
H	24.985601	17.981167	15.486535
H	16.121283	22.049744	25.127580
H	21.455025	23.613972	27.506155
H	14.425254	24.819246	18.607192
H	19.735904	26.433373	21.024458
H	25.387611	12.187043	8.813319
H	26.772797	19.243308	10.665916
H	29.857159	13.137105	12.529854
H	31.168935	20.225062	14.437546

=== ^{3}**Int2** ===

Cd	19.415000	24.466000	24.406000
Cd	16.370000	24.133000	21.810000
Cd	29.590000	17.101000	12.821000
Cd	27.131000	15.292000	10.287000
O	30.396000	14.953000	13.259000
O	26.325000	17.440000	9.849000
O	28.931000	14.769000	11.743000
O	27.789000	17.624000	11.364000
O	29.974000	19.426000	13.017000
O	26.747000	12.968000	10.091000
O	31.101000	18.203000	14.445000
O	25.620000	14.190000	8.663000
O	19.734000	23.407000	26.461000
O	16.050000	25.192000	19.755000
O	21.353000	24.735000	25.823000
O	14.431000	23.864000	20.393000
O	20.336000	25.881000	22.872000
O	15.448000	22.718000	23.344000
O	17.349000	23.397000	24.245000
O	18.436000	25.202000	21.971000
C	17.335403	21.041949	21.113529
C	18.144331	19.998197	20.696871
C	19.370279	20.279942	20.051280
C	19.641523	21.642239	19.793218
C	18.780236	22.617735	20.257720
C	20.362660	19.303475	19.735797
C	20.471210	17.949955	20.350816
C	20.649486	16.817741	19.353938
C	20.243494	15.543103	19.751986
C	20.491943	14.461216	18.924359
C	21.142477	14.593445	17.710724
C	21.554585	15.865702	17.300886
C	21.275850	16.972683	18.115621
C	22.316934	16.032700	16.049898
C	23.144148	15.089471	15.573901
C	23.971185	15.176069	14.364858
C	25.082923	14.334131	14.250991
C	25.903661	14.428581	13.137384
C	24.605029	16.104893	12.238923
C	23.728921	16.069904	13.314792
C	26.828643	17.447736	14.506484
C	25.955235	17.405956	15.582957
C	26.253852	16.607984	16.693250
C	27.450785	15.879767	16.650334
C	28.271068	15.978717	15.537610
C	25.318109	16.578494	17.823844
C	25.441341	15.829241	18.929027
C	24.496091	15.820602	20.054118
C	23.586128	16.859952	20.275540
C	22.658336	16.802346	21.314087
C	22.661263	15.702180	22.171787
C	23.589745	14.691777	21.967643
C	24.503077	14.728108	20.929485
C	21.644854	17.909338	21.511715
C	22.323160	19.228341	21.698329
C	21.707946	20.351073	22.318721
C	22.308399	21.630743	22.264249
C	21.634604	22.727686	22.769693
C	19.856189	21.421271	23.442457
C	20.452281	20.272179	22.961520
C	29.736209	14.230614	12.515935
C	26.955232	18.158788	10.620953
C	30.763403	19.304261	13.982639
C	25.904279	13.093853	9.174108
C	19.507698	25.857991	21.936539
C	16.295270	22.710667	24.263146
C	20.867962	23.913979	26.621183
C	14.948725	24.627008	19.559543
N	25.676345	15.304832	12.153485
N	17.655044	22.332634	20.930929
N	27.966775	16.747550	14.481755
N	20.419383	22.631591	23.332548
F	20.086163	13.247633	19.311231
F	23.596485	13.645622	22.800543
H	16.398113	20.859261	21.633012
H	17.827140	18.978389	20.886089
H	20.555868	21.930041	19.283879
H	18.987784	23.673566	20.114886
H	21.215640	19.657625	19.161324
H	19.548450	17.741941	20.899408
H	19.748452	15.376295	20.703950
H	21.292956	13.717070	17.090272
H	21.590137	17.957364	17.779503
H	22.223932	16.992541	15.541509
H	23.283118	14.182098	16.159894
H	25.311932	13.616965	15.033001
H	26.773155	13.789307	13.012235
H	24.457019	16.784259	11.403108
H	22.859059	16.718401	13.319753
H	26.627209	18.054046	13.627641
H	27.760954	15.250536	17.476902
H	29.211404	15.437233	15.471433
H	24.446400	17.221594	17.713698
H	26.278793	15.140416	19.024202
H	23.600009	17.729169	19.623760
H	21.962502	15.619758	22.998300
H	25.202015	13.907419	20.805912
H	21.095081	17.676248	22.430100
H	23.313083	19.363451	21.272183
H	23.284635	21.758089	21.807096
H	22.058269	23.727833	22.729420
H	18.886176	21.396453	23.931151
H	19.929142	19.327227	23.061297
H	25.038814	17.988160	15.556557
H	16.113825	22.060501	25.134247
H	21.456270	23.610880	27.504371
H	14.427377	24.815845	18.605359
H	19.735377	26.435200	21.025727
H	25.391925	12.186256	8.809360
H	26.773292	19.243399	10.665862
H	29.849905	13.136377	12.536473
H	31.166820	20.225157	14.439274

=== ^{1}**Int3 (CP1-1)** ===

Cd	19.415000	24.466000	24.406000
Cd	16.370000	24.133000	21.810000
Cd	29.590000	17.101000	12.821000
Cd	27.131000	15.292000	10.287000
O	30.396000	14.953000	13.259000
O	26.325000	17.440000	9.849000
O	28.931000	14.769000	11.743000
O	27.789000	17.624000	11.364000
O	29.974000	19.426000	13.017000
O	26.747000	12.968000	10.091000
O	31.101000	18.203000	14.445000
O	25.620000	14.190000	8.663000
O	19.734000	23.407000	26.461000
O	16.050000	25.192000	19.755000
O	21.353000	24.735000	25.823000
O	14.431000	23.864000	20.393000
O	20.336000	25.881000	22.872000
O	15.448000	22.718000	23.344000
O	17.349000	23.397000	24.245000
O	18.436000	25.202000	21.971000
C	17.190482	21.030376	21.029478
C	18.000741	19.971233	20.625563
C	19.193973	20.237059	19.954546

C	19.445897	21.572388	19.618592
C	18.593115	22.570170	20.061513
C	20.307599	19.240180	19.742053
C	20.103024	17.755961	20.120058
C	20.304760	16.666705	19.097949
C	19.873035	15.385697	19.453221
C	20.149316	14.316246	18.618939
C	20.858589	14.463002	17.440082
C	21.304526	15.737173	17.078643
C	20.997789	16.832891	17.898781
C	22.135649	15.921335	15.876476
C	22.991929	14.988194	15.434453
C	23.875840	15.106313	14.269525
C	25.019011	14.302407	14.202736
C	25.878880	14.424506	13.122108
C	24.563746	16.061512	12.173234
C	23.647570	15.995816	13.213050
C	26.773275	17.452849	14.429395
C	25.863627	17.415899	15.475755
C	26.139715	16.651994	16.615726
C	27.347089	15.939183	16.623929
C	28.202892	16.030116	15.537103
C	25.168894	16.637108	17.718169
C	25.339337	16.038892	18.905781
C	24.367350	16.033977	20.009146
C	23.330064	16.969934	20.107498
C	22.397615	16.915538	21.144108
C	22.515490	15.911190	22.108422
C	23.559246	15.003731	22.018737
C	24.487550	15.047514	20.994063
C	21.235151	17.870068	21.222702
C	21.471680	19.349290	20.829978
C	21.249694	20.422105	21.857240
C	21.885598	21.659153	21.729743
C	21.444998	22.737320	22.487231
C	19.887571	21.428158	23.562731
C	20.265701	20.306142	22.841352
C	29.736665	14.230735	12.515831
C	26.956128	18.158894	10.620074
C	30.762564	19.304325	13.983141
C	25.904170	13.093814	9.174478
C	19.505432	25.862049	21.937905
C	16.295133	22.711327	24.263583
C	20.870907	23.911553	26.618479
C	14.951121	24.621949	19.557167
N	25.661144	15.294462	12.130448
N	17.502542	22.307091	20.793416
N	27.924941	16.774218	14.456635
N	20.437176	22.628633	23.358010
F	19.713300	13.100097	18.963112
F	23.672139	14.054966	22.954788
H	16.278552	20.863006	21.597180
H	17.705120	18.962588	20.891393
H	20.343247	21.841616	19.068961
H	18.786162	23.620126	19.860043
H	20.715605	19.383749	18.737915
H	19.139882	17.581816	20.606016
H	19.328459	15.207888	20.375605
H	21.033971	13.597367	16.811198
H	21.351202	17.815390	17.596669
H	22.070640	16.887912	15.376350
H	23.104428	14.071418	16.011173
H	25.238653	13.591448	14.992983
H	26.772241	13.813339	13.029961
H	24.428085	16.736752	11.331935
H	22.756168	16.613643	13.183953
H	26.590336	18.034809	13.530590
H	27.632925	15.319101	17.465999
H	29.150345	15.497675	15.512603
H	24.237195	17.163353	17.516569
H	26.254049	15.481173	19.099377
H	23.257884	17.754008	19.359987
H	21.816183	15.831927	22.934626
H	25.284458	14.311567	20.964992
H	20.796952	17.782348	22.219768
H	22.441572	19.503667	20.349747
H	22.677732	21.801465	21.000757
H	21.875562	23.729671	22.382183
H	19.099148	21.389366	24.309770
H	19.739262	19.373149	23.011516
H	24.938429	17.980390	15.405296
H	16.111848	22.063979	25.136209
H	21.461872	23.601929	27.497550
H	14.435768	24.801829	18.597951
H	19.727688	26.447353	21.031244
H	25.390535	12.186478	8.810726
H	26.775778	19.243894	10.663402
H	29.851077	13.136589	12.535768
H	31.166038	20.225099	14.439969

=== ^{1}**Int3** (**CP1-1**, using its X-ray structure) ===

Cd	21.053000	24.654000	21.773000
Cd	24.128000	24.010000	24.604000
Cd	13.895000	15.373000	36.046000
Cd	11.393000	17.126000	33.519000
O	15.355000	14.312000	37.737000
O	9.932000	18.187000	31.828000
O	14.343000	13.048000	36.246000
O	10.945000	19.451000	33.319000
O	14.722000	17.493000	36.505000
O	10.565000	15.006000	33.060000
O	13.155000	17.653000	34.973000
O	12.132000	14.846000	34.592000
O	20.375000	25.984000	23.441000
O	24.806000	22.679000	22.936000
O	19.098000	24.825000	20.420000
O	26.083000	23.839000	25.957000
O	20.749000	23.561000	19.717000
O	24.432000	25.103000	26.660000
O	23.133000	23.658000	21.887000
O	22.047000	25.006000	24.489000
F	16.360854	14.213277	22.964369
F	20.225367	12.897301	26.430898
N	13.011415	16.932441	31.830849
N	20.072386	22.762156	22.772097
N	15.230593	15.229052	34.085215
N	22.993638	22.140008	25.503100
C	13.618643	18.049437	31.413573
H	13.433404	18.941303	32.008447
C	14.408788	18.082778	30.274172
H	14.865262	19.016659	29.960819
C	14.576171	16.918630	29.514099
C	13.958390	15.752992	29.980569
H	14.086272	14.807291	29.464755
C	13.182842	15.807483	31.127873
H	12.659973	14.929370	31.495472
C	15.367836	16.958751	28.280152
H	16.077167	17.781428	28.215363
C	15.231094	16.105784	27.253754
H	14.477420	15.322301	27.317412
C	16.022205	16.122868	26.016583
C	15.789029	15.153965	25.034384
H	15.006145	14.410615	25.143358
C	16.587927	15.139539	23.902516
C	17.628620	16.036881	23.715959
H	18.242282	15.960029	22.823989
C	17.864435	17.010763	24.688699
C	17.041520	17.056769	25.809948
H	17.232747	17.804767	26.570846
C	19.071756	17.901250	24.594475
H	19.479702	17.838304	23.582466
C	18.919349	19.361594	25.086569
H	17.950172	19.537446	25.560549
C	19.206623	20.495970	24.146612
C	18.662184	21.755575	24.408588
H	17.912748	21.883817	25.183813
C	19.129155	22.861440	23.712792
H	18.765678	23.865483	23.916561
C	20.538519	21.553724	22.449036
H	21.269220	21.527337	21.644550

C	20.138737	20.401539	23.112326
H	20.597164	19.455289	22.844688
C	15.383780	14.026370	33.517159
H	14.916595	13.196627	34.041355
C	16.115364	13.845861	32.350546
H	16.216556	12.850234	31.929815
C	16.728113	14.941410	31.731933
C	16.555421	16.192226	32.339745
H	16.991811	17.092633	31.922883
C	15.809145	16.285774	33.501626
H	15.671483	17.241202	33.997452
C	17.496856	14.736045	30.498961
H	17.484526	13.717391	30.116101
C	18.176897	15.682628	29.835289
H	18.170294	16.705653	30.208889
C	18.932896	15.485452	28.590975
C	19.250422	14.213635	28.103867
H	19.009315	13.306762	28.646164
C	19.915482	14.112550	26.895485
C	20.267290	15.222038	26.142912
H	20.761513	15.075974	25.187168
C	19.959827	16.498525	26.618344
C	19.318543	16.612961	27.851805
H	19.065882	17.590922	28.251989
C	20.203611	17.655749	25.681120
H	21.159760	17.488520	25.179134
C	20.049918	19.109076	26.181353
H	19.619323	19.181522	27.183370
C	21.204192	20.071757	26.085752
C	21.027479	21.359738	26.603444
H	20.151353	21.598419	27.199553
C	21.928934	22.361909	26.283716
H	21.794049	23.385723	26.621536
C	23.227778	20.895206	25.080380
H	24.112989	20.763272	24.463845
C	22.366980	19.836523	25.353953
H	22.597616	18.860626	24.942117
C	15.137500	13.208894	37.207080
C	10.202647	19.296605	32.314743
C	14.001982	18.171012	35.746652
C	11.267262	14.330123	33.838575
C	21.101629	25.816690	24.447204
C	24.060645	22.826667	21.940656
C	19.588068	24.019519	19.604759
C	25.559482	24.579234	26.813274
H	9.781208	20.204355	31.847246
H	11.136967	13.236694	33.860222
H	15.667318	12.320496	37.593169
H	14.125329	19.265714	35.733642
H	18.973817	23.688713	18.749797
H	24.244430	22.181363	21.065555
H	26.108043	24.780031	27.749260
H	20.869268	26.411299	25.345906

=== ^{3}**Int3** ===

Cd	19.415000	24.466000	24.406000
Cd	16.370000	24.133000	21.810000
Cd	29.590000	17.101000	12.821000
Cd	27.131000	15.292000	10.287000
O	30.396000	14.953000	13.259000
O	26.325000	17.440000	9.849000
O	28.931000	14.769000	11.743000
O	27.789000	17.624000	11.364000
O	29.974000	19.426000	13.017000
O	26.747000	12.968000	10.091000
O	31.101000	18.203000	14.445000
O	25.620000	14.190000	8.663000
O	19.734000	23.407000	26.461000
O	16.050000	25.192000	19.755000
O	21.353000	24.735000	25.823000
O	14.431000	23.864000	20.393000
O	20.336000	25.881000	22.872000
O	15.448000	22.718000	23.344000
O	17.349000	23.397000	24.245000
O	18.436000	25.202000	21.971000

C	17.186692	21.020435	21.091858
C	18.002773	19.961723	20.699527
C	19.180065	20.228880	20.002759
C	19.415688	21.558935	19.636956
C	18.559825	22.556435	20.071422
C	20.297234	19.243573	19.776197
C	20.119623	17.751682	20.136083
C	20.401265	16.705525	19.092304
C	19.962634	15.396269	19.339962
C	20.417063	14.359867	18.529906
C	21.331435	14.551560	17.517074
C	21.786317	15.867820	17.253512
C	21.250913	16.934392	18.023665
C	22.851398	16.129196	16.370630
C	23.767099	15.088848	15.876400
C	24.355299	15.080947	14.596823
C	25.389808	14.161318	14.292656
C	26.044356	14.249413	13.082851
C	24.747784	16.047456	12.419182
C	24.024023	16.028619	13.595206
C	26.936021	17.652607	14.585726
C	26.127503	17.740857	15.709817
C	26.383146	16.926587	16.818738
C	27.486203	16.064873	16.735628
C	28.255706	16.051745	15.582280
C	25.481221	16.996745	17.976030
C	25.389754	16.080812	18.952857
C	24.413108	16.067066	20.051509
C	23.401347	17.028208	20.184524
C	22.423762	16.931150	21.174706
C	22.485501	15.866433	22.079024
C	23.506914	14.936879	21.964726
C	24.462425	15.009472	20.966014
C	21.253400	17.879937	21.243991
C	21.475497	19.358450	20.846041
C	21.263478	20.432761	21.872929
C	21.882459	21.675781	21.722987
C	21.442723	22.754600	22.479081
C	19.921989	21.434979	23.595504
C	20.300617	20.311614	22.876672
C	29.738551	14.231346	12.512745
C	26.957418	18.158998	10.617935
C	30.762823	19.304204	13.983472
C	25.898599	13.095100	9.179193
C	19.504930	25.862755	21.938123
C	16.295862	22.710214	24.262944
C	20.871283	23.911351	26.618228
C	14.950811	24.621871	19.557220
N	25.751944	15.192007	12.172037
N	17.482345	22.294421	20.822073
N	27.982656	16.824914	14.520475
N	20.452264	22.639686	23.368504
F	19.977272	13.121823	18.779455
F	23.561667	13.926726	22.839422
H	16.284522	20.857822	21.676271
H	17.726082	18.954386	20.990485
H	20.301909	21.824283	19.067819
H	18.738864	23.604616	19.847956
H	20.682188	19.400490	18.765606
H	19.156242	17.533469	20.604074
H	19.299849	15.165822	20.167849
H	21.691574	13.701927	16.947196
H	21.624503	17.933077	17.816409
H	23.147732	17.167314	16.225374
H	24.135720	14.367682	16.604962
H	25.677226	13.404262	15.015652
H	26.838041	13.561222	12.807263
H	24.544285	16.771055	11.633822
H	23.223623	16.743356	13.756052
H	26.756633	18.259466	13.702126
H	27.769403	15.430602	17.568484
H	29.131569	15.414570	15.488011
H	24.809207	17.852823	17.975799
H	26.058696	15.221368	18.930318
H	23.358612	17.844995	19.473204

H	21.749592	15.747559	22.867886
H	25.228747	14.243392	20.906643
H	20.809699	17.792909	22.238700
H	22.435988	19.520222	20.349777
H	22.659093	21.820992	20.978117
H	21.858459	23.751512	22.358892
H	19.148165	21.393965	24.357773
H	19.789072	19.373898	23.064332
H	25.294027	18.437537	15.716379
H	16.114247	22.060517	25.134210
H	21.462507	23.600977	27.496869
H	14.435562	24.802040	18.598040
H	19.726413	26.449293	21.032115
H	25.375175	12.189612	8.824771
H	26.780664	19.244798	10.657376
H	29.857035	13.137388	12.529523
H	31.165842	20.225236	14.440320

=== ^{1}**TS2** ===

Cd	19.415000	24.466000	24.406000
Cd	16.370000	24.133000	21.810000
Cd	29.590000	17.101000	12.821000
Cd	27.131000	15.292000	10.287000
O	30.396000	14.953000	13.259000
O	26.325000	17.440000	9.849000
O	28.931000	14.769000	11.743000
O	27.789000	17.624000	11.364000
O	29.974000	19.426000	13.017000
O	26.747000	12.968000	10.091000
O	31.101000	18.203000	14.445000
O	25.620000	14.190000	8.663000
O	19.734000	23.407000	26.461000
O	16.050000	25.192000	19.755000
O	21.353000	24.735000	25.823000
O	14.431000	23.864000	20.393000
O	20.336000	25.881000	22.872000
O	15.448000	22.718000	23.344000
O	17.349000	23.397000	24.245000
O	18.436000	25.202000	21.971000
C	17.155470	21.016658	21.041872
C	17.955034	19.950015	20.635275
C	19.139923	20.205820	19.946689
C	19.392323	21.536850	19.593808
C	18.552099	22.542354	20.042161
C	20.255744	19.210909	19.740973
C	20.080875	17.729596	20.154583
C	20.232956	16.640844	19.123344
C	19.564340	15.435642	19.339511
C	19.741952	14.387579	18.447686
C	20.572493	14.480793	17.348379
C	21.262882	15.678598	17.125262
C	21.069855	16.748451	18.009446
C	22.204766	15.840894	16.008213
C	22.648202	14.859552	15.206429
C	23.618909	15.018048	14.116819
C	24.196140	13.878714	13.542812
C	25.144892	14.012353	12.537533
C	24.980261	16.304672	12.607474
C	24.027341	16.258950	13.612426
C	27.417718	15.528851	14.671633
C	26.551923	15.260161	15.721015
C	26.287022	16.258808	16.662341
C	26.910355	17.498205	16.486061
C	27.758778	17.685099	15.404714
C	25.362731	16.008596	17.782898
C	25.528618	16.504215	19.011896
C	24.532424	16.294670	20.095093
C	23.419926	17.134703	20.155092
C	22.444736	16.984834	21.143708
C	22.593729	15.959172	22.077948
C	23.704573	15.131646	22.009424
C	24.684952	15.280645	21.041106
C	21.241438	17.885918	21.220894
C	21.432404	19.365054	20.806258
C	21.210469	20.439215	21.829982
C	21.843196	21.676808	21.696107
C	21.414933	22.750847	22.465165
C	19.874797	21.434972	23.560346
C	20.241127	20.316958	22.827469
C	29.770793	14.234261	12.487765
C	27.002864	18.164882	10.573447
C	30.785470	19.303302	13.966298
C	25.907498	13.093603	9.164651
C	19.505673	25.861385	21.937466
C	16.295497	22.710859	24.263276
C	20.870969	23.911429	26.618380
C	14.951401	24.621175	19.556842
N	25.539872	15.207919	12.079851
N	17.471508	22.289988	20.792467
N	28.003741	16.720959	14.510308
N	20.422536	22.635604	23.352167
F	19.079180	13.245511	18.661357
F	23.835806	14.156933	22.916503
H	16.250881	20.859160	21.624063
H	17.660744	18.944099	20.913404
H	20.284012	21.796935	19.030688
H	18.748907	23.590176	19.832181
H	20.646054	19.327448	18.725733
H	19.136372	17.559892	20.677561
H	18.900184	15.297572	20.186988
H	20.653509	13.628554	16.683723
H	21.603551	17.674427	17.814562
H	22.588996	16.851325	15.873367
H	22.307594	13.838883	15.369003
H	23.909027	12.887403	13.880165
H	25.610410	13.151627	12.063212
H	25.313093	17.252080	12.199829
H	23.605072	17.186709	13.982173
H	27.637846	14.782604	13.913976
H	26.717606	18.316410	17.171728
H	28.254389	18.636110	15.228953
H	24.508615	15.364037	17.576348
H	26.413637	17.096931	19.245474
H	23.330923	17.914109	19.405048
H	21.865864	15.799394	22.867083
H	25.538577	14.612159	21.029139
H	20.827191	17.799381	22.228576
H	22.388635	19.541772	20.308390
H	22.623983	21.821482	20.955549
H	21.842785	23.744195	22.358651
H	19.099223	21.393618	24.320576
H	19.719575	19.381702	23.000831
H	26.091492	14.281372	15.807297
H	16.112983	22.062357	25.135245
H	21.462061	23.601587	27.497312
H	14.436983	24.799854	18.596904
H	19.728508	26.445589	21.030264
H	25.405126	12.183832	8.792002
H	26.894993	19.259670	10.545743
H	29.930222	13.146061	12.461470
H	31.223592	20.222964	14.393142

=== ^{3}**TS2** ===

Cd	19.415000	24.466000	24.406000
Cd	16.370000	24.133000	21.810000
Cd	29.590000	17.101000	12.821000
Cd	27.131000	15.292000	10.287000
O	30.396000	14.953000	13.259000
O	26.325000	17.440000	9.849000
O	28.931000	14.769000	11.743000
O	27.789000	17.624000	11.364000
O	29.974000	19.426000	13.017000
O	26.747000	12.968000	10.091000
O	31.101000	18.203000	14.445000
O	25.620000	14.190000	8.663000
O	19.734000	23.407000	26.461000
O	16.050000	25.192000	19.755000
O	21.353000	24.735000	25.823000
O	14.431000	23.864000	20.393000
O	20.336000	25.881000	22.872000

O	15.448000	22.718000	23.344000
O	17.349000	23.397000	24.245000
O	18.436000	25.202000	21.971000
C	17.085022	21.044963	21.004145
C	17.864488	19.974605	20.574421
C	19.038099	20.228459	19.867350
C	19.300565	21.557060	19.515597
C	18.480694	22.566721	19.987903
C	20.137101	19.227082	19.639520
C	19.942992	17.739700	20.017155
C	20.106892	16.709990	18.929494
C	19.316107	15.553163	18.955074
C	19.531393	14.553108	18.007996
C	20.515799	14.633996	17.049868
C	21.328330	15.795998	17.005219
C	21.082482	16.829731	17.949201
C	22.419189	15.913624	16.119816
C	23.049967	14.776035	15.417889
C	23.708141	14.841642	14.168971
C	24.615713	13.816464	13.800442
C	25.446647	13.990490	12.711072
C	24.497771	16.039301	12.220734
C	23.631848	15.958908	13.295870
C	26.865216	16.995102	14.509286
C	25.989534	16.761971	15.552043
C	26.485760	16.403448	16.807924
C	27.871570	16.335845	16.964503
C	28.687046	16.590543	15.866374
C	25.508312	16.098221	17.866489
C	25.551580	16.480446	19.144653
C	24.395123	16.177092	20.031373
C	23.433487	17.169014	20.258168
C	22.254387	16.888517	20.944950
C	22.060821	15.597311	21.447777
C	23.030730	14.633544	21.238807
C	24.194510	14.891706	20.526724
C	21.113886	17.865715	21.079988
C	21.324890	19.349325	20.697005
C	21.125463	20.415630	21.738969
C	21.761380	21.652652	21.605971
C	21.359273	22.722700	22.395988
C	19.834958	21.409890	23.509296
C	20.175123	20.295417	22.755361
C	29.730461	14.230695	12.524913
C	26.963236	18.161470	10.611137
C	30.749102	19.305934	13.992048
C	25.894063	13.096313	9.180180
C	19.505167	25.861956	21.937729
C	16.295029	22.711582	24.263786
C	20.869800	23.913401	26.619816
C	14.953028	24.618345	19.555413
N	25.422904	15.100073	11.950953
N	17.414775	22.314443	20.757527
N	28.188340	16.894390	14.661663
N	20.387126	22.610152	23.306729
F	18.754460	13.465157	18.051391
F	22.832264	13.401548	21.721019
H	16.190165	20.896229	21.603577
H	17.568573	18.968777	20.850491
H	20.184785	21.809193	18.937428
H	18.682285	23.615197	19.785459
H	20.521006	19.350816	18.622799
H	18.993820	17.553262	20.525396
H	18.541275	15.410382	19.701228
H	20.651698	13.821242	16.344566
H	21.732868	17.700517	17.927023
H	22.979472	16.847049	16.160434
H	23.214878	13.873475	16.007265
H	24.698873	12.919457	14.405995
H	26.165123	13.232495	12.410893
H	24.483020	16.888680	11.542199
H	22.922269	16.758537	13.486111
H	26.517574	17.265407	13.516110
H	28.311267	16.054142	17.915568
H	29.770815	16.551725	15.931645
H	24.619914	15.563192	17.529057
H	26.380471	17.074601	19.528634
H	23.614738	18.153471	19.839896
H	21.158549	15.328900	21.989196
H	24.916016	14.099149	20.362888
H	20.704482	17.765230	22.089174
H	22.269366	19.536075	20.182332
H	22.527927	21.801901	20.851363
H	21.795581	23.712245	22.288133
H	19.075896	21.363372	24.285590
H	19.648391	19.363796	22.933065
H	24.925351	16.860019	15.387230
H	16.111829	22.064358	25.136564
H	21.458610	23.607801	27.501658
H	14.442079	24.791421	18.592511
H	19.727519	26.447438	21.031264
H	25.365157	12.191578	8.832385
H	26.800968	19.249039	10.639785
H	29.832700	13.135895	12.554880
H	31.129205	20.227275	14.467879

=== [1]**Int4** ===

Cd	19.415000	24.466000	24.406000
Cd	16.370000	24.133000	21.810000
Cd	29.590000	17.101000	12.821000
Cd	27.131000	15.292000	10.287000
O	30.396000	14.953000	13.259000
O	26.325000	17.440000	9.849000
O	28.931000	14.769000	11.743000
O	27.789000	17.624000	11.364000
O	29.974000	19.426000	13.017000
O	26.747000	12.968000	10.091000
O	31.101000	18.203000	14.445000
O	25.620000	14.190000	8.663000
O	19.734000	23.407000	26.461000
O	16.050000	25.192000	19.755000
O	21.353000	24.735000	25.823000
O	14.431000	23.864000	20.393000
O	20.336000	25.881000	22.872000
O	15.448000	22.718000	23.344000
O	17.349000	23.397000	24.245000
O	18.436000	25.202000	21.971000
C	17.089813	21.044169	20.986978
C	17.866196	19.970131	20.559934
C	19.051835	20.216516	19.869240
C	19.327025	21.546037	19.529834
C	18.508445	22.559726	19.998121
C	20.150870	19.206689	19.663637
C	19.921906	17.723925	20.031897
C	20.130372	16.632877	19.009652
C	19.692325	15.353367	19.361100
C	19.990364	14.279621	18.538637
C	20.732920	14.418648	17.380228
C	21.186599	15.691281	17.020283
C	20.847471	16.791457	17.822414
C	22.053876	15.876124	15.843765
C	22.857889	14.918500	15.353332
C	23.753058	15.040185	14.194790
C	24.763173	14.089400	14.009391
C	25.643261	14.216627	12.943382
C	24.587806	16.130225	12.217732
C	23.667176	16.074883	13.253951
C	26.907337	16.903479	14.740126
C	26.159354	16.604618	15.865604
C	26.771337	15.955781	16.947436
C	28.124018	15.627604	16.816244
C	28.803967	15.969296	15.656289
C	26.049108	15.636705	18.185712
C	24.922603	16.265655	18.544594
C	24.112240	16.070691	19.748239
C	23.088578	16.993625	19.966134
C	22.205184	16.886977	21.038307
C	22.375686	15.835658	21.936368
C	23.414669	14.933646	21.731664
C	24.280774	15.018292	20.656699

C	21.036963	17.824635	21.157849
C	21.288807	19.305238	20.778484
C	21.082137	20.382706	21.803470
C	21.743655	21.605714	21.666591
C	21.350721	22.688792	22.441776
C	19.791103	21.410120	23.550328
C	20.117544	20.284720	22.807552
C	29.724376	14.230118	12.528431
C	26.959977	18.160323	10.616986
C	30.757380	19.304739	13.986961
C	25.900345	13.094830	9.176429
C	19.505082	25.862447	21.938027
C	16.294062	22.713166	24.264614
C	20.870378	23.912213	26.619037
C	14.952724	24.619202	19.555773
N	25.568015	15.227844	12.071324
N	17.430910	22.313296	20.753504
N	28.204042	16.594441	14.634741
N	20.367202	22.597589	23.341962
F	19.546760	13.064922	18.879050
F	23.567584	13.931305	22.605069
H	16.186730	20.896867	21.574310
H	17.555973	18.966606	20.828008
H	20.220597	21.796665	18.965500
H	18.721114	23.607396	19.803269
H	20.582293	19.350845	18.669249
H	18.948200	17.560103	20.499636
H	19.129071	15.178004	20.272775
H	20.927212	13.545771	16.767395
H	21.202327	17.773194	17.520847
H	22.050264	16.868603	15.393049
H	22.904678	13.961323	15.869901
H	24.869704	13.259137	14.700298
H	26.430295	13.490039	12.760473
H	24.549392	16.909261	11.460621
H	22.879760	16.819260	13.307714
H	26.466097	17.398672	13.879110
H	28.650503	15.123515	17.620135
H	29.856053	15.737359	15.521498
H	26.503511	14.891124	18.835315
H	24.545675	17.048992	17.888912
H	22.970777	17.797306	19.245823
H	21.717088	15.697041	22.787991
H	25.051498	14.264828	20.537105
H	20.594417	17.714508	22.151133
H	22.268375	19.448801	20.316706
H	22.522798	21.732526	20.920962
H	21.803282	23.670802	22.331405
H	19.026197	21.384860	24.321755
H	19.574454	19.362731	22.985683
H	25.102349	16.846480	15.881039
H	16.109009	22.068462	25.138914
H	21.460578	23.604385	27.499210
H	14.440757	24.793678	18.593671
H	19.726778	26.448975	21.032091
H	25.381609	12.188241	8.818160
H	26.787940	19.246400	10.653621
H	29.816584	13.134509	12.568980
H	31.151462	20.225958	14.451058

=== ^{3}**Int4** ===

Cd	19.415000	24.466000	24.406000
Cd	16.370000	24.133000	21.810000
Cd	29.590000	17.101000	12.821000
Cd	27.131000	15.292000	10.287000
O	30.396000	14.953000	13.259000
O	26.325000	17.440000	9.849000
O	28.931000	14.769000	11.743000
O	27.789000	17.624000	11.364000
O	29.974000	19.426000	13.017000
O	26.747000	12.968000	10.091000
O	31.101000	18.203000	14.445000
O	25.620000	14.190000	8.663000
O	19.734000	23.407000	26.461000
O	16.050000	25.192000	19.755000
O	21.353000	24.735000	25.823000
O	14.431000	23.864000	20.393000
O	20.336000	25.881000	22.872000
O	15.448000	22.718000	23.344000
O	17.349000	23.397000	24.245000
O	18.436000	25.202000	21.971000
C	17.133221	21.047858	21.022788
C	17.929942	19.985706	20.604840
C	19.104316	20.249686	19.902032
C	19.353101	21.580246	19.548107
C	18.515590	22.581737	20.008016
C	20.210240	19.254645	19.678246
C	20.006176	17.760902	20.023652
C	20.225173	16.756731	18.924015
C	19.384418	15.641580	18.825577
C	19.665759	14.658259	17.878676
C	20.775144	14.701410	17.064916
C	21.657342	15.808499	17.170288
C	21.315080	16.850961	18.069684
C	22.900836	15.850029	16.505196
C	23.459312	14.727009	15.734525
C	24.093660	14.856226	14.476733
C	24.693239	13.737512	13.844929
C	25.434999	13.911871	12.693889
C	25.020838	16.183901	12.683096
C	24.255739	16.106225	13.832490
C	27.651845	15.435923	14.797903
C	26.982832	15.052846	15.949633
C	26.794439	15.976859	16.981106
C	27.364060	17.246774	16.814283
C	28.036518	17.540631	15.639160
C	25.951294	15.602089	18.121877
C	25.396537	16.473858	18.976212
C	24.355661	16.146976	19.957549
C	23.432779	17.151542	20.269638
C	22.279554	16.886730	21.004973
C	22.100063	15.599301	21.515927
C	23.054245	14.625276	21.258142
C	24.171332	14.862361	20.475643
C	21.142412	17.868882	21.129910
C	21.374826	19.353346	20.763704
C	21.167354	20.417699	21.803053
C	21.799508	21.655848	21.664120
C	21.385209	22.729637	22.441035
C	19.859925	21.415780	23.555613
C	20.211800	20.297291	22.813781
C	29.782096	14.236454	12.476348
C	26.987434	18.163708	10.588947
C	30.785352	19.303819	13.965298
C	25.898993	13.095183	9.173131
C	19.504543	25.862925	21.938209
C	16.294856	22.711832	24.263917
C	20.870530	23.912198	26.618952
C	14.952635	24.618998	19.555706
N	25.621908	15.120613	12.127557
N	17.446284	22.320555	20.770070
N	28.160135	16.661183	14.636573
N	20.406193	22.616558	23.343693
F	18.833724	13.614032	17.786711
F	22.874624	13.402310	21.768900
H	16.236114	20.889644	21.616340
H	17.644388	18.978040	20.884992
H	20.236788	21.841010	18.973076
H	18.704478	23.631332	19.799755
H	20.614578	19.393218	18.670953
H	19.039471	17.559452	20.490872
H	18.520675	15.519312	19.470937
H	20.965826	13.887828	16.373677
H	21.982726	17.705372	18.128533
H	23.556417	16.695129	16.708048
H	23.477238	13.744798	16.207111
H	24.585579	12.745868	14.274554
H	25.912579	13.081071	12.180763
H	25.168120	17.130486	12.173580
H	23.805672	17.002431	14.245420

H	27.773387	14.752091	13.963684
H	27.299996	18.001189	17.590449
H	28.497357	18.511092	15.481658
H	25.677265	14.549380	18.178546
H	25.614416	17.535570	18.868311
H	23.607036	18.138070	19.852486
H	21.223355	15.335676	22.099615
H	24.876546	14.059120	20.292516
H	20.700467	17.758591	22.123943
H	22.331703	19.533459	20.270168
H	22.569739	21.802190	20.912749
H	21.814772	23.721642	22.328893
H	19.095635	21.373332	24.327148
H	19.689740	19.363837	22.994570
H	26.578510	14.048729	16.028637
H	16.111321	22.064997	25.136924
H	21.460709	23.604050	27.499053
H	14.440862	24.793395	18.593532
H	19.725794	26.450246	21.032648
H	25.381472	12.188257	8.814566
H	26.856578	19.256181	10.583774
H	29.957718	13.151146	12.433338
H	31.222750	20.223711	14.392710

=== ^{3}**TS3** ===

Cd	19.415000	24.466000	24.406000
Cd	16.370000	24.133000	21.810000
Cd	29.590000	17.101000	12.821000
Cd	27.131000	15.292000	10.287000
O	30.396000	14.953000	13.259000
O	26.325000	17.440000	9.849000
O	28.931000	14.769000	11.743000
O	27.789000	17.624000	11.364000
O	29.974000	19.426000	13.017000
O	26.747000	12.968000	10.091000
O	31.101000	18.203000	14.445000
O	25.620000	14.190000	8.663000
O	19.734000	23.407000	26.461000
O	16.050000	25.192000	19.755000
O	21.353000	24.735000	25.823000
O	14.431000	23.864000	20.393000
O	20.336000	25.881000	22.872000
O	15.448000	22.718000	23.344000
O	17.349000	23.397000	24.245000
O	18.436000	25.202000	21.971000
C	17.007128	21.071018	20.952001
C	17.759907	19.988776	20.508953
C	18.937657	20.225527	19.802876
C	19.228076	21.549528	19.457743
C	18.432510	22.572321	19.942400
C	20.019821	19.205248	19.590339
C	19.762671	17.724221	19.936749
C	19.991039	16.654114	18.899610
C	19.515545	15.367355	19.188740
C	19.962563	14.290172	18.431280
C	20.900601	14.419942	17.429490
C	21.377272	15.711813	17.105261
C	20.851946	16.818621	17.822588
C	22.439234	15.925224	16.183420
C	23.382641	14.923892	15.756403
C	24.059060	14.978806	14.471178
C	24.958379	13.960825	14.099866
C	25.710214	14.084496	12.944206
C	24.725653	16.099295	12.435824
C	23.931736	16.054795	13.573067
C	28.235200	15.469136	15.244899
C	27.274293	15.034718	16.141709
C	26.136572	15.815821	16.359052
C	26.039221	17.029524	15.664099
C	27.021021	17.365457	14.750507
C	25.076806	15.349720	17.249356
C	24.471654	16.265425	18.120815
C	23.722309	15.973381	19.301576
C	22.944296	17.019722	19.831571
C	22.011613	16.813093	20.837446
C	21.946764	15.550358	21.435104
C	22.777740	14.535144	20.967654
C	23.632179	14.698467	19.895745
C	20.901086	17.798869	21.061991
C	21.157972	19.282457	20.706788
C	20.965545	20.352883	21.744551
C	21.636978	21.570785	21.605032
C	21.275776	22.651623	22.398316
C	19.725173	21.383085	23.527746
C	20.019535	20.259572	22.766319
C	29.752356	14.232079	12.498015
C	26.964169	18.160277	10.612578
C	30.762294	19.303380	13.985370
C	25.896608	13.095738	9.179197
C	19.503817	25.864188	21.938841
C	16.292926	22.715049	24.265766
C	20.869498	23.913503	26.620095
C	14.954342	24.616317	19.554316
N	25.621642	15.151718	12.134258
N	17.367783	22.333427	20.716419
N	28.093478	16.596267	14.535786
N	20.312500	22.565733	23.321036
F	19.490199	13.072385	18.720213
F	22.707061	13.336722	21.557160
H	16.112429	20.938723	21.555648
H	17.442930	18.988110	20.778731
H	20.116693	21.786533	18.879937
H	18.654301	23.618624	19.749979
H	20.451856	19.350618	18.596816
H	18.789072	17.558625	20.403255
H	18.832560	15.184524	20.012431
H	21.226749	13.535372	16.894715
H	21.246652	17.800753	17.578885
H	22.611108	16.950640	15.861862
H	23.264588	13.928510	16.177730
H	25.090357	13.089471	14.735518
H	26.411554	13.315322	12.631079
H	24.653543	16.921167	11.728095
H	23.218492	16.852244	13.750312
H	29.143558	14.905643	15.060716
H	25.170900	17.670674	15.778578
H	26.953041	18.266580	14.146199
H	25.155514	14.312304	17.568805
H	24.565130	17.321143	17.875245
H	23.012670	17.981801	19.334797
H	21.241454	15.330102	22.230382
H	24.219856	13.853426	19.555194
H	20.476322	17.662001	22.060122
H	22.134862	19.440506	20.246980
H	22.402833	21.695881	20.845354
H	21.739974	23.628104	22.285967
H	18.977734	21.359217	24.315885
H	19.469711	19.342046	22.948270
H	27.411968	14.096801	16.670325
H	16.105944	22.073339	25.141886
H	21.458261	23.608664	27.502178
H	14.445993	24.785001	18.589204
H	19.723714	26.454216	21.034794
H	25.371997	12.190288	8.826747
H	26.798286	19.247812	10.641496
H	29.888524	13.140082	12.495157
H	31.161986	20.225365	14.443489

=== ^{3}**Int5** ===

Cd	19.415000	24.466000	24.406000
Cd	16.370000	24.133000	21.810000
Cd	29.590000	17.101000	12.821000
Cd	27.131000	15.292000	10.287000
O	30.396000	14.953000	13.259000
O	26.325000	17.440000	9.849000
O	28.931000	14.769000	11.743000
O	27.789000	17.624000	11.364000
O	29.974000	19.426000	13.017000
O	26.747000	12.968000	10.091000
O	31.101000	18.203000	14.445000

O	25.620000	14.190000	8.663000
O	19.734000	23.407000	26.461000
O	16.050000	25.192000	19.755000
O	21.353000	24.735000	25.823000
O	14.431000	23.864000	20.393000
O	20.336000	25.881000	22.872000
O	15.448000	22.718000	23.344000
O	17.349000	23.397000	24.245000
O	18.436000	25.202000	21.971000
C	17.053759	21.087226	20.940673
C	17.827384	20.017762	20.503657
C	19.016995	20.273568	19.824537
C	19.302682	21.603903	19.501271
C	18.484837	22.613139	19.976496
C	20.101723	19.258758	19.601647
C	19.835808	17.772191	19.910409
C	20.018276	16.747839	18.818089
C	19.462532	15.476951	19.009791
C	19.828260	14.441273	18.161925
C	20.765866	14.594596	17.158113
C	21.321474	15.869408	16.936927
C	20.876568	16.942334	17.739828
C	22.415009	16.086796	16.027306
C	23.502500	15.086353	15.822905
C	24.045350	14.998602	14.407707
C	24.966557	13.997662	14.086011
C	25.679178	14.073262	12.898276
C	24.552785	15.974482	12.267816
C	23.787804	15.960653	13.430488
C	28.214560	15.632048	15.330882
C	27.179958	15.190292	16.143745
C	25.979981	15.895291	16.158864
C	25.914671	17.090103	15.438776
C	26.980118	17.450456	14.632628
C	24.763371	15.357315	16.870626
C	24.410866	16.292729	17.972404
C	23.735072	15.979392	19.182538
C	23.022548	17.035474	19.793568
C	22.076116	16.810790	20.778801
C	21.962686	15.521124	21.316312
C	22.743426	14.499594	20.787894
C	23.589227	14.679925	19.708826
C	20.980498	17.809126	21.031895
C	21.243212	19.301964	20.716364
C	21.043638	20.355182	21.770439
C	21.712065	21.577103	21.651821
C	21.324435	22.654502	22.437755
C	19.754389	21.375038	23.525688
C	20.076289	20.253689	22.771656
C	29.745252	14.231054	12.506735
C	26.962066	18.160057	10.613718
C	30.759000	19.303450	13.988222
C	25.893341	13.096437	9.182400
C	19.503822	25.864142	21.938961
C	16.293333	22.714312	24.265284
C	20.869717	23.912750	26.619625
C	14.954482	24.616431	19.554341
N	25.512722	15.076970	12.026997
N	17.404931	22.355260	20.723067
N	28.093486	16.714484	14.556073
N	20.337168	22.562904	23.334452
F	19.280215	13.235570	18.352459
F	22.623922	13.272962	21.309423
H	16.147229	20.938866	21.522418
H	17.513685	19.011346	20.754706
H	20.200033	21.854865	18.943360
H	18.697382	23.663295	19.796501
H	20.535117	19.422688	18.611848
H	18.865928	17.603749	20.383353
H	18.779288	15.271609	19.828039
H	21.035303	13.734472	16.555142
H	21.338168	17.911503	17.575295
H	22.634365	17.122231	15.773352
H	23.136829	14.097408	16.116294
H	25.180640	13.195483	14.787202

H	26.422497	13.329424	12.622807
H	24.423765	16.743450	11.510458
H	23.039084	16.730236	13.583338
H	29.154850	15.095000	15.249907
H	25.012825	17.695170	15.436832
H	26.945928	18.331213	13.997070
H	25.010153	14.370545	17.276691
H	24.515472	17.350843	17.744705
H	23.120689	18.016736	19.341203
H	21.245100	15.288206	22.096737
H	24.112033	13.823368	19.297658
H	20.554084	17.651611	22.026675
H	22.219486	19.469298	20.258077
H	22.493256	21.709553	20.909179
H	21.785302	23.633883	22.337381
H	18.985588	21.345318	24.292825
H	19.527808	19.332119	22.937536
H	27.294443	14.274094	16.714987
H	16.107100	22.071512	25.140741
H	21.459242	23.606694	27.500810
H	14.445947	24.785136	18.589304
H	19.723814	26.454159	21.034859
H	25.361890	12.192386	8.836576
H	26.795872	19.247331	10.643627
H	29.872222	13.138197	12.512865
H	31.152078	20.225639	14.451609

=== ^{1}**Int6 (CP1-2β)** ===

Cd	19.415000	24.466000	24.406000
Cd	16.370000	24.133000	21.810000
Cd	29.590000	17.101000	12.821000
Cd	27.131000	15.292000	10.287000
O	30.396000	14.953000	13.259000
O	26.325000	17.440000	9.849000
O	28.931000	14.769000	11.743000
O	27.789000	17.624000	11.364000
O	29.974000	19.426000	13.017000
O	26.747000	12.968000	10.091000
O	31.101000	18.203000	14.445000
O	25.620000	14.190000	8.663000
O	19.734000	23.407000	26.461000
O	16.050000	25.192000	19.755000
O	21.353000	24.735000	25.823000
O	14.431000	23.864000	20.393000
O	20.336000	25.881000	22.872000
O	15.448000	22.718000	23.344000
O	17.349000	23.397000	24.245000
O	18.436000	25.202000	21.971000
C	17.005934	21.085963	20.930611
C	17.763037	20.014627	20.471534
C	18.927476	20.269231	19.749790
C	19.201598	21.596987	19.407610
C	18.401445	22.608454	19.906935
C	20.011212	19.259585	19.512919
C	19.746438	17.765792	19.792212
C	19.983353	16.772215	18.684449
C	19.329536	15.536212	18.709139
C	19.776369	14.523427	17.876172
C	20.920865	14.643957	17.096318
C	21.576983	15.871301	17.065447
C	21.027303	16.944276	17.776043
C	22.990936	16.062733	16.578374
C	23.823283	14.886137	16.016400
C	24.245753	14.857639	14.565478
C	25.201230	13.913680	14.173018
C	25.840198	14.043015	12.949248
C	24.592098	15.896083	12.420961
C	23.895370	15.827272	13.626545
C	28.385810	15.641746	15.412933
C	27.402466	15.183314	16.278877
C	26.185242	15.856186	16.344111
C	26.057944	17.042701	15.616143
C	27.073175	17.419858	14.756141
C	24.999398	15.244412	17.024879
C	24.079867	16.249778	17.741163

C	23.566866	15.945815	19.117698
C	22.921004	17.006890	19.755788
C	21.965069	16.799503	20.746257
C	21.835816	15.512680	21.277075
C	22.604821	14.488376	20.746401
C	23.421106	14.661265	19.634224
C	20.865408	17.803857	20.954281
C	21.140928	19.294687	20.639568
C	20.955480	20.352831	21.694267
C	21.619787	21.575986	21.559629
C	21.261552	22.650364	22.363676
C	19.724978	21.370399	23.494763
C	20.017862	20.251962	22.723408
C	29.744562	14.230979	12.507675
C	26.958387	18.159326	10.616881
C	30.756675	19.303430	13.990283
C	25.895047	13.096016	9.180790
C	19.504030	25.863574	21.938629
C	16.293247	22.714509	24.265493
C	20.869176	23.913939	26.620407
C	14.955361	24.614385	19.553434
N	25.575853	15.050377	12.105098
N	17.350991	22.352265	20.694294
N	28.196427	16.708792	14.630333
N	20.305828	22.557732	23.294503
F	19.132476	13.349943	17.885292
F	22.486362	13.260405	21.264705
H	16.121718	20.942836	21.547116
H	17.462581	19.008656	20.740790
H	20.079445	21.844331	18.817996
H	18.606445	23.658427	19.715793
H	20.447122	19.437778	18.526152
H	18.764295	17.577106	20.230981
H	18.503948	15.338481	19.385641
H	21.277025	13.772237	16.558043
H	21.544912	17.897336	17.727048
H	23.040117	16.939275	15.923332
H	23.401038	13.918873	16.307235
H	25.498369	13.115867	14.848178
H	26.603147	13.341951	12.621777
H	24.385482	16.674462	11.690797
H	23.129155	16.567008	13.833766
H	29.334563	15.128351	15.291110
H	25.146730	17.630951	15.645670
H	26.985334	18.292297	14.113676
H	25.319042	14.396860	17.637038
H	24.493221	17.263054	17.741346
H	23.052755	17.994020	19.326768
H	21.115746	15.283923	22.056672
H	23.881340	13.787603	19.184253
H	20.397862	17.653830	21.931436
H	22.120578	19.450494	20.184085
H	22.379055	21.710655	20.794887
H	21.723401	23.628214	22.253440
H	18.981779	21.337063	24.286619
H	19.472262	19.331850	22.904896
H	27.567479	14.275984	16.851772
H	16.106827	22.072062	25.141171
H	21.457285	23.610269	27.503303
H	14.449233	24.779607	18.586495
H	19.724638	26.452690	21.034122
H	25.367075	12.191265	8.831828
H	26.787282	19.245595	10.651604
H	29.869644	13.137833	12.515438
H	31.144589	20.225970	14.457484

=== ^{1}**Int6** (**CP1-2β**, using its X-ray structure) ===

Cd	21.064000	13.318000	21.193000
Cd	32.855000	21.023000	10.496000
Cd	31.643000	24.478000	12.639000
Cd	21.091000	16.376000	23.992000
O	21.038000	11.444000	19.697000
O	20.204000	13.359000	19.039000
O	20.441000	15.542000	21.398000
O	20.044000	17.505000	22.366000
O	21.657000	14.159000	23.857000
O	22.100000	12.255000	22.875000
O	22.194000	16.511000	25.986000
O	21.167000	18.405000	25.457000
O	31.228000	26.475000	14.037000
O	29.573000	25.466000	12.913000
O	33.876000	24.637000	12.792000
O	33.767000	22.803000	11.605000
O	35.040000	20.162000	10.206000
O	33.393000	19.070000	9.217000
O	30.618000	20.884000	10.244000
O	30.673000	22.675000	11.551000
F	32.582491	14.045361	19.006193
F	32.905600	17.660414	22.111819
C	23.268747	14.849237	19.394077
H	22.348816	15.049267	18.850011
C	24.462498	15.489114	19.092828
H	24.491927	16.228958	18.298105
C	25.599256	15.228426	19.863208
C	25.498549	14.239665	20.841072
H	26.340570	13.969496	21.468386
C	24.272415	13.635202	21.073217
H	24.142505	12.883104	21.845466
N	23.171792	13.964403	20.392040
N	32.754690	19.846037	12.541547
C	23.279228	17.753824	21.939412
H	22.346647	18.181300	21.581883
C	24.473903	17.937837	21.262097
H	24.483960	18.508235	20.337772
C	25.642810	17.332138	21.734550
C	25.557671	16.649631	22.947583
H	26.423712	16.176212	23.397424
C	24.321483	16.525151	23.576993
H	24.206939	15.994099	24.518855
N	23.197068	17.029641	23.060934
C	26.795271	16.126497	19.745159
H	26.833352	16.526057	18.728512
C	26.836378	17.306625	20.816423
H	26.890133	18.273623	20.310091
C	28.217395	16.818967	21.313071
H	28.194259	16.415300	22.328795
C	28.166989	15.617342	20.239987
H	28.098401	14.658631	20.759098
C	29.419910	17.711893	21.151946
C	30.587865	17.412631	21.860257
H	30.585392	16.735094	22.708290
C	31.791723	17.945164	21.426834
C	31.906446	18.682437	20.254151
H	32.897938	18.947919	19.901950
C	30.744321	19.001973	19.557183
C	29.511183	18.600064	20.081857
H	28.620627	18.856843	19.517280
C	30.691255	16.824935	17.698528
C	31.760075	15.965063	17.940947
H	32.743403	16.109763	17.507253
C	31.563537	14.887949	18.796716
C	30.387995	14.703301	19.505846
H	30.337036	13.917699	20.253224
C	29.311944	15.563028	19.266154
C	29.453403	16.536817	18.279483
H	28.629326	17.209049	18.062532
C	30.831782	18.196507	17.093525
H	30.066777	18.340735	16.324107
C	30.727016	19.428026	18.116440
H	29.825269	20.021212	17.935542
C	31.983224	20.052350	17.467833
H	32.798763	20.261810	18.164918
C	32.206682	18.717279	16.631879
H	33.028473	18.159253	17.091422
C	32.462705	18.917129	15.159485
C	31.531708	18.598952	14.171871
H	30.644685	18.016666	14.394230
C	31.707421	19.093085	12.883343
H	30.970834	18.921295	12.102690
C	33.714655	20.060176	13.450000

H	34.568681	20.638147	13.107231
C	33.609132	19.610567	14.756878
H	34.385967	19.858492	15.474698
C	31.751904	21.248951	16.591243
C	30.646924	21.314541	15.737855
H	29.845521	20.584610	15.794962
C	30.614162	22.272976	14.738770
H	29.805027	22.320393	14.015313
C	32.610756	23.178856	15.442834
H	33.366820	23.941965	15.278544
C	32.723191	22.241646	16.462738
H	33.591725	22.255757	17.114125
C	34.417280	23.678423	12.204340
C	34.597216	19.201674	9.529809
C	30.026483	26.341149	13.714476
C	30.064854	21.810804	10.883898
C	20.529085	12.165698	18.822597
C	19.985124	16.710437	21.391524
C	22.147358	13.015352	23.865273
C	21.911926	17.730314	26.187819
N	31.594597	23.166348	14.575497
H	20.358902	11.751356	17.814439
H	22.652580	12.654568	24.775675
H	22.370772	18.209423	27.070894
H	19.513034	17.079897	20.466688
H	29.287068	27.030205	14.159242
H	35.516816	23.607704	12.230757
H	35.311020	18.428330	9.196782
H	28.962717	21.848746	10.858605

=== ^{3}**Int7** ===

Cd	19.415000	24.466000	24.406000
Cd	16.370000	24.133000	21.810000
Cd	29.590000	17.101000	12.821000
Cd	27.131000	15.292000	10.287000
O	30.396000	14.953000	13.259000
O	26.325000	17.440000	9.849000
O	28.931000	14.769000	11.743000
O	27.789000	17.624000	11.364000
O	29.974000	19.426000	13.017000
O	26.747000	12.968000	10.091000
O	31.101000	18.203000	14.445000
O	25.620000	14.190000	8.663000
O	19.734000	23.407000	26.461000
O	16.050000	25.192000	19.755000
O	21.353000	24.735000	25.823000
O	14.431000	23.864000	20.393000
O	20.336000	25.881000	22.872000
O	15.448000	22.718000	23.344000
O	17.349000	23.397000	24.245000
O	18.436000	25.202000	21.971000
C	17.052757	21.058775	21.117405
C	17.810882	19.974540	20.702875
C	18.910579	20.191372	19.871357
C	19.119998	21.487194	19.398834
C	18.319477	22.519524	19.862442
C	19.943890	19.125771	19.599670
C	19.309434	17.805779	19.308866
C	19.739675	16.872782	18.325499
C	19.017232	15.669138	18.129441
C	19.530265	14.704822	17.291365
C	20.774857	14.824714	16.672844
C	21.494250	16.005019	16.852446
C	20.934467	17.039545	17.596665
C	22.939249	16.163067	16.462196
C	23.758674	14.961125	15.943811
C	24.207507	14.904285	14.505188
C	25.158063	13.944954	14.141580
C	25.814577	14.053074	12.925803
C	24.589228	15.912688	12.355463
C	23.875764	15.864981	13.550772
C	28.311910	15.649037	15.395336
C	27.312430	15.216669	16.256105
C	26.111402	15.920322	16.318114
C	26.021415	17.111881	15.592872
C	27.053306	17.464506	14.740117
C	24.908959	15.319147	16.980486
C	23.957215	16.317280	17.671591
C	23.384422	15.949759	19.011238
C	22.866054	16.985612	19.777721
C	22.016311	16.758403	20.880811
C	21.848712	15.420485	21.307745
C	22.433754	14.405711	20.579228
C	23.166811	14.631107	19.416023
C	21.253865	17.809281	21.468936
C	21.089710	19.137654	20.815483
C	20.878381	20.281661	21.786043
C	21.500862	21.509831	21.549771
C	21.172763	22.612445	22.328094
C	19.747684	21.353803	23.619295
C	20.015346	20.207822	22.881264
C	29.745549	14.231123	12.506578
C	26.958910	18.159431	10.616567
C	30.757634	19.303418	13.989517
C	25.893888	13.096260	9.182010
C	19.502463	25.865459	21.939402
C	16.292535	22.716823	24.267211
C	20.870059	23.913512	26.619860
C	14.954051	24.615184	19.554119
N	25.569240	15.053233	12.068438
N	17.334330	22.309089	20.739513
N	28.157971	16.723791	14.615175
N	20.285527	22.542191	23.324523
F	18.836333	13.576041	17.099373
F	22.250606	13.139699	20.973868
H	16.218257	20.949296	21.805173
H	17.572884	18.989618	21.090112
H	19.940839	21.703898	18.721867
H	18.473608	23.552139	19.559624
H	20.505497	19.445573	18.717004
H	18.437555	17.523175	19.889878
H	18.077658	15.486941	18.640186
H	21.148695	13.993132	16.085554
H	21.508089	17.953157	17.715346
H	23.051800	17.031559	15.804360
H	23.306931	14.009498	16.244144
H	25.436746	13.154215	14.832646
H	26.577284	13.343726	12.615853
H	24.402841	16.682726	11.611250
H	23.111586	16.611597	13.739126
H	29.246672	15.109218	15.277317
H	25.128013	17.726980	15.620888
H	26.993402	18.343099	14.103094
H	25.214591	14.474697	17.604100
H	24.377119	17.325842	17.737641
H	23.060110	17.999955	19.446510
H	21.230855	15.173017	22.164451
H	23.524100	13.775074	18.852615
H	20.642804	17.568109	22.333527
H	22.009069	19.377472	20.276791
H	22.208898	21.623445	20.733990
H	21.607230	23.592346	22.146661
H	19.062396	21.344283	24.462572
H	19.509196	19.283968	23.141488
H	27.450005	14.303156	16.826548
H	16.104232	22.077011	25.144466
H	21.458730	23.608422	27.501881
H	14.446689	24.783058	18.588344
H	19.721017	26.458110	21.036815
H	25.363115	12.192099	8.835537
H	26.788289	19.245841	10.650886
H	29.872732	13.138215	12.512471
H	31.147415	20.225854	14.455312

=== ^{1}**TS4** ===

Cd	19.415000	24.466000	24.406000
Cd	16.370000	24.133000	21.810000
Cd	29.590000	17.101000	12.821000
Cd	27.131000	15.292000	10.287000
O	30.396000	14.953000	13.259000

O	26.325000	17.440000	9.849000
O	28.931000	14.769000	11.743000
O	27.789000	17.624000	11.364000
O	29.974000	19.426000	13.017000
O	26.747000	12.968000	10.091000
O	31.101000	18.203000	14.445000
O	25.620000	14.190000	8.663000
O	19.734000	23.407000	26.461000
O	16.050000	25.192000	19.755000
O	21.353000	24.735000	25.823000
O	14.431000	23.864000	20.393000
O	20.336000	25.881000	22.872000
O	15.448000	22.718000	23.344000
O	17.349000	23.397000	24.245000
O	18.436000	25.202000	21.971000
C	17.039598	21.051501	21.107270
C	17.757381	19.956623	20.653382
C	18.876270	20.163320	19.841262
C	19.153116	21.474192	19.445565
C	18.390505	22.515095	19.947881
C	19.836461	19.081282	19.489275
C	19.240334	17.787504	19.222523
C	19.724029	16.811837	18.294936
C	19.015717	15.601605	18.114097
C	19.545302	14.627151	17.295513
C	20.792199	14.750305	16.685372
C	21.502282	15.937521	16.858535
C	20.929150	16.975076	17.588081
C	22.944519	16.108237	16.464521
C	23.775978	14.913289	15.950304
C	24.221930	14.862044	14.510414
C	25.186760	13.918696	14.143268
C	25.837594	14.038751	12.925566
C	24.581224	15.879046	12.360811
C	23.871271	15.817580	13.557476
C	28.324682	15.647823	15.400642
C	27.329497	15.207455	16.262650
C	26.120128	15.896873	16.319812
C	26.016222	17.083121	15.587632
C	27.044285	17.443627	14.733740
C	24.923095	15.287820	16.985286
C	23.963371	16.284166	17.668714
C	23.393179	15.928981	19.012036
C	22.889147	16.972397	19.777346
C	22.044576	16.755429	20.883441
C	21.854736	15.424415	21.310775
C	22.426149	14.398628	20.583405
C	23.161201	14.613779	19.421398
C	21.316122	17.828125	21.492801
C	21.154810	19.122691	20.869539
C	20.882943	20.276629	21.781010
C	21.511057	21.505202	21.552668
C	21.167261	22.610574	22.319507
C	19.696811	21.362947	23.570742
C	19.979041	20.213901	22.845676
C	29.745426	14.231136	12.506761
C	26.959018	18.159476	10.616327
C	30.757725	19.303477	13.989340
C	25.893843	13.096244	9.182104
C	19.503383	25.863893	21.938720
C	16.294802	22.713297	24.264757
C	20.869213	23.914503	26.620639
C	14.953222	24.617595	19.555165
N	25.574489	15.036376	12.070504
N	17.376814	22.306510	20.792427
N	28.158385	16.716310	14.614447
N	20.254336	22.547324	23.293223
F	18.861279	13.490988	17.113951
F	22.225879	13.136660	20.981192
H	16.186375	20.945027	21.772272
H	17.467515	18.965522	20.984782
H	19.991764	21.688479	18.790018
H	18.596995	23.552731	19.699227
H	20.484828	19.409052	18.675882
H	18.348382	17.511038	19.776340
H	18.069800	15.421766	18.613929
H	21.176314	13.916027	16.108437
H	21.494282	17.894750	17.701567
H	23.043669	16.973201	15.800150
H	23.335551	13.956703	16.251823
H	25.480740	13.132025	14.832599
H	26.609502	13.341078	12.612481
H	24.380334	16.647419	11.618621
H	23.094957	16.550996	13.747747
H	29.266024	15.118802	15.286482
H	25.115360	17.687422	15.611258
H	26.973750	18.317451	14.091254
H	25.234742	14.450211	17.615342
H	24.375013	17.296747	17.722736
H	23.096813	17.985067	19.448218
H	21.235619	15.187324	22.169543
H	23.508847	13.753245	18.858417
H	20.736003	17.596417	22.381731
H	21.991409	19.377342	20.220457
H	22.244888	21.612028	20.759259
H	21.613155	23.587336	22.149192
H	18.988820	21.358402	24.394893
H	19.461984	19.292332	23.092484
H	27.477996	14.298865	16.838205
H	16.111035	22.066253	25.137868
H	21.456819	23.611603	27.504154
H	14.443106	24.789414	18.591503
H	19.723959	26.453442	21.034445
H	25.362893	12.192147	8.835754
H	26.788824	19.245930	10.650186
H	29.872363	13.138190	12.512947
H	31.147960	20.225840	14.454830

=== [3]**TS4** ===

Cd	19.415000	24.466000	24.406000
Cd	16.370000	24.133000	21.810000
Cd	29.590000	17.101000	12.821000
Cd	27.131000	15.292000	10.287000
O	30.396000	14.953000	13.259000
O	26.325000	17.440000	9.849000
O	28.931000	14.769000	11.743000
O	27.789000	17.624000	11.364000
O	29.974000	19.426000	13.017000
O	26.747000	12.968000	10.091000
O	31.101000	18.203000	14.445000
O	25.620000	14.190000	8.663000
O	19.734000	23.407000	26.461000
O	16.050000	25.192000	19.755000
O	21.353000	24.735000	25.823000
O	14.431000	23.864000	20.393000
O	20.336000	25.881000	22.872000
O	15.448000	22.718000	23.344000
O	17.349000	23.397000	24.245000
O	18.436000	25.202000	21.971000
C	17.078532	21.062785	21.092769
C	17.741346	19.965229	20.574091
C	18.874893	20.160927	19.759910
C	19.229628	21.494595	19.478056
C	18.515346	22.530988	20.045465
C	19.709013	19.086286	19.285151
C	19.252169	17.729538	19.250112
C	19.761585	16.696910	18.424807
C	19.156860	15.411311	18.465271
C	19.707745	14.387131	17.736948
C	20.892494	14.530028	17.007690
C	21.505471	15.780391	16.959484
C	20.902999	16.858846	17.604432
C	22.916726	15.990248	16.480420
C	23.774803	14.821338	15.946121
C	24.211901	14.794189	14.502237
C	25.190114	13.869153	14.123759
C	25.840092	14.012821	12.908302
C	24.558942	15.841618	12.364908
C	23.848356	15.755447	13.560113
C	28.332076	15.637447	15.396882

C	27.340303	15.176352	16.252500
C	26.115354	15.838009	16.298052
C	25.988092	17.016495	15.557108
C	27.013276	17.397064	14.708664
C	24.928059	15.215159	16.968863
C	23.966543	16.204746	17.656147
C	23.410585	15.868497	19.007272
C	22.867541	16.921222	19.733272
C	22.009905	16.713942	20.832243
C	21.804227	15.384572	21.262428
C	22.410312	14.354947	20.574317
C	23.189505	14.559097	19.436111
C	21.301611	17.796364	21.448057
C	21.362167	19.126748	21.009247
C	20.969312	20.270312	21.820097
C	21.541737	21.526526	21.561628
C	21.133921	22.634953	22.286789
C	19.662086	21.366657	23.520148
C	20.011773	20.208320	22.844979
C	29.745107	14.231095	12.507197
C	26.959435	18.159595	10.615678
C	30.758317	19.303569	13.988673
C	25.893885	13.096233	9.181868
C	19.503412	25.862916	21.938246
C	16.297756	22.709285	24.261810
C	20.867682	23.916648	26.622282
C	14.952262	24.620572	19.556499
N	25.564511	15.017322	12.064914
N	17.471239	22.321444	20.858331
N	28.146944	16.698102	14.604167
N	20.196451	22.561913	23.237964
F	19.126469	13.182464	17.757057
F	22.209281	13.096707	20.982512
H	16.209282	20.953465	21.736575
H	17.382485	18.973498	20.822884
H	20.078734	21.711819	18.836991
H	18.775056	23.571110	19.867518
H	20.466508	19.380573	18.563792
H	18.399875	17.464781	19.868108
H	18.269096	15.227157	19.061100
H	21.301791	13.656094	16.513459
H	21.388333	17.828274	17.544785
H	22.955915	16.856800	15.812521
H	23.369617	13.847773	16.239506
H	25.495702	13.078785	14.803807
H	26.620129	13.328088	12.587148
H	24.347497	16.616559	11.632470
H	23.062619	16.476717	13.757766
H	29.286708	15.130643	15.293504
H	25.071331	17.597059	15.569391
H	26.925361	18.263891	14.058896
H	25.252403	14.383053	17.599874
H	24.359783	17.225495	17.681592
H	23.065224	17.928624	19.383462
H	21.166882	15.155869	22.110172
H	23.573809	13.693726	18.905316
H	20.686116	17.553281	22.310881
H	22.104370	19.371078	20.255562
H	22.301660	21.636945	20.793903
H	21.554845	23.621641	22.109649
H	18.924435	21.358483	24.317486
H	19.523560	19.275039	23.104376
H	27.506733	14.274785	16.834103
H	16.119206	22.053388	25.129679
H	21.453038	23.618306	27.508876
H	14.438534	24.797341	18.595586
H	19.726130	26.449761	21.032471
H	25.363133	12.192081	8.835442
H	26.790345	19.246215	10.648518
H	29.871406	13.138075	12.514058
H	31.150021	20.225785	14.453087

=== ^{1}**Int8** ===

Cd	19.415000	24.466000	24.406000
Cd	16.370000	24.133000	21.810000
Cd	29.590000	17.101000	12.821000
Cd	27.131000	15.292000	10.287000
O	30.396000	14.953000	13.259000
O	26.325000	17.440000	9.849000
O	28.931000	14.769000	11.743000
O	27.789000	17.624000	11.364000
O	29.974000	19.426000	13.017000
O	26.747000	12.968000	10.091000
O	31.101000	18.203000	14.445000
O	25.620000	14.190000	8.663000
O	19.734000	23.407000	26.461000
O	16.050000	25.192000	19.755000
O	21.353000	24.735000	25.823000
O	14.431000	23.864000	20.393000
O	20.336000	25.881000	22.872000
O	15.448000	22.718000	23.344000
O	17.349000	23.397000	24.245000
O	18.436000	25.202000	21.971000
C	16.859104	21.070897	20.928602
C	17.378206	19.954175	20.289515
C	18.624611	20.042439	19.658264
C	19.300711	21.265707	19.737948
C	18.705719	22.336673	20.386346
C	19.235514	18.931977	18.917798
C	18.567240	17.933303	18.322873
C	19.202104	16.861945	17.540375
C	18.495639	15.683381	17.268556
C	19.132409	14.664593	16.581831
C	20.456287	14.751782	16.169061
C	21.159483	15.926205	16.427839
C	20.514106	16.977370	17.077028
C	22.620480	16.100995	16.114617
C	23.480126	14.902195	15.672047
C	23.970948	14.814885	14.254178
C	24.955277	13.868250	13.957779
C	25.684808	13.988618	12.787972
C	24.452973	15.820432	12.129442
C	23.665013	15.757490	13.274409
C	28.087714	15.639615	15.303201
C	27.034252	15.222959	16.107283
C	25.825168	15.917695	16.075801
C	25.773676	17.077802	15.297385
C	26.861532	17.417380	14.507895
C	24.597595	15.336223	16.716713
C	23.592838	16.337965	17.334519
C	23.007762	15.979123	18.669560
C	22.589386	17.011136	19.502573
C	21.871844	16.765061	20.677783
C	21.580980	15.443697	21.027698
C	22.031173	14.421781	20.207306
C	22.729220	14.659293	19.033214
C	21.373191	17.874646	21.498670
C	21.889441	19.112370	21.485420
C	21.336523	20.277595	22.177429
C	21.969257	21.517758	22.035886
C	21.404984	22.644624	22.615792
C	19.671582	21.402268	23.490349
C	20.159076	20.234177	22.934498
C	29.743358	14.230991	12.508823
C	26.961805	18.160053	10.613321
C	30.762234	19.303675	13.984991
C	25.890281	13.097079	9.185215
C	19.505960	25.861247	21.937059
C	16.298675	22.706271	24.259713
C	20.867671	23.914698	26.621674
C	14.948520	24.629092	19.559984
N	25.472060	14.986578	11.920189
N	17.506675	22.243079	20.970696
N	27.986232	16.694137	14.487005
N	20.271187	22.587332	23.319143
F	18.456032	13.539615	16.321282
F	21.765065	13.156894	20.549986
H	15.893955	21.045721	21.428214
H	16.818191	19.025303	20.293825
H	20.277971	21.386167	19.280512

H	19.187687	23.307892	20.451333
H	20.319521	18.978780	18.832138
H	17.482389	17.886136	18.408102
H	17.469895	15.546395	17.594816
H	20.898781	13.908520	15.649549
H	21.062256	17.900836	17.238171
H	22.738366	16.939919	15.420515
H	23.049514	13.948708	15.996348
H	25.208278	13.091924	14.674613
H	26.482042	13.298671	12.525741
H	24.302559	16.584666	11.371268
H	22.872885	16.485334	13.412139
H	29.032177	15.103808	15.268911
H	24.874287	17.683630	15.244564
H	26.835324	18.273605	13.839546
H	24.891046	14.529032	17.393632
H	23.978685	17.360933	17.379792
H	22.775993	18.036129	19.193916
H	21.021427	15.200306	21.924719
H	23.013201	13.810326	18.418927
H	20.513729	17.646029	22.125798
H	22.785325	19.308134	20.899083
H	22.891254	21.604549	21.469348
H	21.857278	23.628852	22.520944
H	18.767247	21.416076	24.089683
H	19.614009	19.309580	23.085833
H	27.143197	14.329755	16.715032
H	16.123779	22.045050	25.124195
H	21.455406	23.613229	27.505729
H	14.425377	24.820234	18.607221
H	19.729658	26.444057	21.029291
H	25.351287	12.194612	8.846595
H	26.796108	19.247393	10.642959
H	29.868043	13.137865	12.517852
H	31.162724	20.225227	14.442865

=== ^{3}**Int8** ===

Cd	19.415000	24.466000	24.406000
Cd	16.370000	24.133000	21.810000
Cd	29.590000	17.101000	12.821000
Cd	27.131000	15.292000	10.287000
O	30.396000	14.953000	13.259000
O	26.325000	17.440000	9.849000
O	28.931000	14.769000	11.743000
O	27.789000	17.624000	11.364000
O	29.974000	19.426000	13.017000
O	26.747000	12.968000	10.091000
O	31.101000	18.203000	14.445000
O	25.620000	14.190000	8.663000
O	19.734000	23.407000	26.461000
O	16.050000	25.192000	19.755000
O	21.353000	24.735000	25.823000
O	14.431000	23.864000	20.393000
O	20.336000	25.881000	22.872000
O	15.448000	22.718000	23.344000
O	17.349000	23.397000	24.245000
O	18.436000	25.202000	21.971000
C	16.926338	21.084689	20.910888
C	17.470359	19.980770	20.285503
C	18.743877	20.085923	19.652658
C	19.371565	21.365027	19.723428
C	18.741414	22.408729	20.358320
C	19.374787	19.030766	18.984528
C	18.789314	17.701081	18.749880
C	19.352724	16.726142	17.931172
C	18.691917	15.466091	17.752374
C	19.301809	14.487240	17.019776
C	20.583194	14.634492	16.462437
C	21.247112	15.852396	16.617352
C	20.617583	16.893137	17.287244
C	22.687911	16.057474	16.228459
C	23.555359	14.875071	15.748874
C	24.028649	14.809309	14.322060
C	25.011082	13.868004	14.001257
C	25.720711	13.996786	12.819557
C	24.478363	15.831732	12.196218
C	23.710183	15.761095	13.355097
C	28.154989	15.645369	15.331609
C	27.117521	15.215155	16.148712
C	25.900927	15.895940	16.131809
C	25.824610	17.057300	15.357638
C	26.896039	17.408890	14.552211
C	24.686477	15.300790	16.782305
C	23.693151	16.293643	17.426562
C	23.132776	15.933835	18.771607
C	22.684441	16.967753	19.585857
C	21.947476	16.726308	20.749775
C	21.675527	15.403686	21.112049
C	22.159393	14.380058	20.313438
C	22.874172	14.613870	19.147843
C	21.407209	17.839484	21.537373
C	21.877890	19.095327	21.480555
C	21.311354	20.262344	22.155978
C	21.959654	21.497616	22.039589
C	21.398193	22.624390	22.622442
C	19.632220	21.394214	23.445236
C	20.114069	20.226636	22.881259
C	29.743892	14.231003	12.508416
C	26.961360	18.160004	10.613713
C	30.761206	19.303673	13.985921
C	25.891473	13.096800	9.184023
C	19.505051	25.861857	21.937552
C	16.297473	22.707699	24.260307
C	20.866933	23.915565	26.622374
C	14.950162	24.626583	19.558836
N	25.492836	14.998916	11.960066
N	17.537982	22.281326	20.952756
N	28.027473	16.697395	14.516235
N	20.251790	22.573185	23.306015
F	18.681553	13.315023	16.840936
F	21.908736	13.114900	20.667092
H	15.957586	21.035675	21.401808
H	16.913834	19.051414	20.282858
H	20.343459	21.516845	19.263554
H	19.187488	23.397000	20.415280
H	20.362331	19.232015	18.582740
H	17.843556	17.469316	19.228047
H	17.718842	15.282148	18.194760
H	21.015004	13.794661	15.929658
H	21.145674	17.835605	17.378741
H	22.762728	16.906758	15.541171
H	23.143319	13.911141	16.065302
H	25.277113	13.085418	14.706510
H	26.513127	13.307915	12.540153
H	24.313545	16.601238	11.446368
H	22.921910	16.489543	13.511205
H	29.105400	15.121672	15.283195
H	24.916390	17.650661	15.317191
H	26.850621	18.263247	13.882233
H	24.992291	14.486132	17.444554
H	24.075284	17.318305	17.463622
H	22.853075	17.990915	19.262639
H	21.102492	15.160833	22.000687
H	23.177994	13.762832	18.545925
H	20.566737	17.598088	22.185578
H	22.762946	19.301484	20.881721
H	22.894871	21.579963	21.494413
H	21.864642	23.603803	22.547363
H	18.715478	21.412011	24.024915
H	19.550342	19.308321	23.001129
H	27.244403	14.322640	16.753950
H	16.121340	22.048607	25.126289
H	21.453700	23.616197	27.507809
H	14.429738	24.812981	18.603494
H	19.728448	26.445955	21.030433
H	25.355315	12.193735	8.842735
H	26.795256	19.247236	10.643758
H	29.869020	13.137917	12.516804
H	31.159556	20.225379	14.445415

=== [1]**TS5** ===

Cd	19.415000	24.466000	24.406000
Cd	16.370000	24.133000	21.810000
Cd	29.590000	17.101000	12.821000
Cd	27.131000	15.292000	10.287000
O	30.396000	14.953000	13.259000
O	26.325000	17.440000	9.849000
O	28.931000	14.769000	11.743000
O	27.789000	17.624000	11.364000
O	29.974000	19.426000	13.017000
O	26.747000	12.968000	10.091000
O	31.101000	18.203000	14.445000
O	25.620000	14.190000	8.663000
O	19.734000	23.407000	26.461000
O	16.050000	25.192000	19.755000
O	21.353000	24.735000	25.823000
O	14.431000	23.864000	20.393000
O	20.336000	25.881000	22.872000
O	15.448000	22.718000	23.344000
O	17.349000	23.397000	24.245000
O	18.436000	25.202000	21.971000
C	17.635379	22.271106	19.529739
C	18.002978	21.144881	18.812458
C	18.034786	19.901211	19.456320
C	17.697738	19.867899	20.810445
C	17.346257	21.043063	21.456099
C	18.447306	18.656693	18.795163
C	18.686308	18.510340	17.489925
C	19.255213	17.254555	16.946619
C	18.494671	16.098220	16.766728
C	19.135466	14.956066	16.312986
C	20.500648	14.910196	16.060400
C	21.255104	16.064544	16.252315
C	20.619383	17.231195	16.673111
C	22.744187	16.121027	16.095717
C	23.563799	14.882717	15.707969
C	24.063676	14.777641	14.295632
C	25.067193	13.853079	13.999056
C	25.773669	13.978393	12.815411
C	24.483044	15.770191	12.153149
C	23.714471	15.697924	13.308507
C	28.129378	15.612537	15.331317
C	27.087823	15.196079	16.151587
C	25.882917	15.899682	16.152371
C	25.833747	17.075061	15.395895
C	26.908292	17.412987	14.587412
C	24.655321	15.303277	16.785083
C	23.630210	16.300523	17.382854
C	22.946838	15.913742	18.663879
C	22.515623	16.933186	19.508027
C	21.780327	16.670578	20.669694
C	21.508583	15.342182	21.009712
C	21.946958	14.335005	20.164755
C	22.649814	14.589647	18.996368
C	21.318473	17.772967	21.527384
C	21.108239	19.016048	21.069734
C	20.753927	20.199906	21.856706
C	20.733208	21.438922	21.209991
C	20.409253	22.579911	21.925409
C	20.114748	21.367182	23.860657
C	20.433900	20.178815	23.220851
C	29.744038	14.231176	12.508229
C	26.959944	18.159673	10.615279
C	30.761833	19.303626	13.985608
C	25.889928	13.097052	9.186185
C	19.480591	25.903136	21.960397
C	16.276025	22.744611	24.282455
C	20.869112	23.920713	26.624322
C	14.941778	24.630759	19.562498
N	25.520733	14.961043	11.942015
N	17.318210	22.222178	20.831219
N	28.022955	16.676741	14.527938
N	20.103782	22.547194	23.224561
F	18.414939	13.844898	16.124537
F	21.680034	13.064175	20.486880
H	17.572092	23.253037	19.065619
H	18.261819	21.246553	17.764388
H	17.716973	18.932059	21.360386
H	17.066013	21.046531	22.503446
H	18.605783	17.802550	19.453257
H	18.549992	19.338768	16.796239
H	17.429801	16.078246	16.969755
H	20.935506	13.977165	15.718197
H	21.208139	18.132341	16.833698
H	22.985441	16.946948	15.420253
H	23.087403	13.951798	16.033606
H	25.350734	13.092238	14.720768
H	26.584788	13.307448	12.547504
H	24.302562	16.520877	11.387989
H	22.899354	16.399211	13.449057
H	29.067367	15.067960	15.272276
H	24.947701	17.700494	15.374531
H	26.876605	18.281444	13.935034
H	24.945757	14.492952	17.459892
H	24.027537	17.314441	17.491029
H	22.785242	17.957309	19.268950
H	20.957317	15.082092	21.907251
H	22.955734	13.748200	18.382427
H	21.162074	17.537155	22.578819
H	21.227202	19.204474	20.003549
H	20.962936	21.511509	20.151556
H	20.385329	23.553844	21.448179
H	19.864559	21.404371	24.918848
H	20.428070	19.253379	23.786420
H	27.200826	14.292836	16.743706
H	16.062139	22.145221	25.182342
H	21.450778	23.627413	27.514733
H	14.416576	24.828585	18.612092
H	19.663490	26.553688	21.090096
H	25.349241	12.195085	8.848738
H	26.791594	19.246490	10.647596
H	29.869116	13.137986	12.516676
H	31.161147	20.225320	14.444166

=== [1]**Int9** ===

Cd	19.415000	24.466000	24.406000
Cd	16.370000	24.133000	21.810000
Cd	29.590000	17.101000	12.821000
Cd	27.131000	15.292000	10.287000
O	30.396000	14.953000	13.259000
O	26.325000	17.440000	9.849000
O	28.931000	14.769000	11.743000
O	27.789000	17.624000	11.364000
O	29.974000	19.426000	13.017000
O	26.747000	12.968000	10.091000
O	31.101000	18.203000	14.445000
O	25.620000	14.190000	8.663000
O	19.734000	23.407000	26.461000
O	16.050000	25.192000	19.755000
O	21.353000	24.735000	25.823000
O	14.431000	23.864000	20.393000
O	20.336000	25.881000	22.872000
O	15.448000	22.718000	23.344000
O	17.349000	23.397000	24.245000
O	18.436000	25.202000	21.971000
C	17.481857	22.215813	19.509429
C	17.722982	21.064735	18.777755
C	17.716936	19.826399	19.431771
C	17.443363	19.814282	20.800817
C	17.218829	21.013222	21.457053
C	18.041440	18.570215	18.750957
C	18.874279	18.509266	17.706253
C	19.381111	17.262809	17.125433
C	18.611152	16.099786	17.014447
C	19.226930	14.947295	16.559955
C	20.581398	14.888948	16.247629
C	21.342728	16.048146	16.362908
C	20.724491	17.230319	16.763330
C	22.827341	16.101049	16.178915
C	23.648220	14.866244	15.782644

C	24.136926	14.770140	14.364272
C	25.143522	13.854951	14.049110
C	25.827465	13.985175	12.852136
C	24.513990	15.767541	12.214432
C	23.766557	15.689322	13.383382
C	28.193222	15.622168	15.359845
C	27.167665	15.198674	16.195834
C	25.959667	15.895909	16.215420
C	25.892543	17.074800	15.466115
C	26.951456	17.419631	14.640981
C	24.743140	15.288480	16.856497
C	23.719428	16.280243	17.463374
C	23.041850	15.892258	18.747809
C	22.564628	16.911384	19.569378
C	21.834329	16.643124	20.732519
C	21.616223	15.311838	21.099534
C	22.096168	14.306618	20.276032
C	22.793220	14.565322	19.104394
C	21.329063	17.745108	21.567719
C	21.021250	18.954428	21.076536
C	20.643959	20.146399	21.843184
C	20.561559	21.369155	21.170801
C	20.256358	22.522835	21.876402
C	20.084457	21.353326	23.850112
C	20.384390	20.154207	23.220850
C	29.744801	14.231266	12.507516
C	26.959413	18.159560	10.615843
C	30.760566	19.303604	13.986764
C	25.890713	13.096854	9.185588
C	19.484679	25.898391	21.957547
C	16.274977	22.746861	24.283691
C	20.867974	23.922778	26.625835
C	14.940126	24.633252	19.563757
N	25.552696	14.965150	11.982221
N	17.244195	22.191185	20.827739
N	28.066421	16.686644	14.560046
N	20.028888	22.520903	23.192121
F	18.496324	13.834808	16.427869
F	21.878360	13.032238	20.621646
H	17.445961	23.197587	19.041676
H	17.878974	21.128836	17.706231
H	17.424459	18.880031	21.352987
H	16.999280	21.038501	22.517975
H	17.669444	17.652509	19.202911
H	19.323230	19.428183	17.330285
H	17.553848	16.088072	17.256202
H	20.999783	13.943873	15.918429
H	21.320812	18.136597	16.855832
H	23.060261	16.929759	15.504386
H	23.177237	13.932349	16.108025
H	25.445380	13.094620	14.763811
H	26.637361	13.318454	12.570477
H	24.313044	16.517196	11.453317
H	22.948507	16.384410	13.536973
H	29.132683	15.082715	15.283076
H	25.003673	17.696227	15.461596
H	26.905864	18.290726	13.992922
H	25.044410	14.477490	17.525485
H	24.113824	17.295494	17.570510
H	22.793147	17.941872	19.313906
H	21.072173	15.048389	22.000442
H	23.133517	13.724483	18.507842
H	21.230016	17.540086	22.632519
H	21.085239	19.109425	20.000091
H	20.733643	21.421934	20.100060
H	20.173920	23.480833	21.374126
H	19.888056	21.409523	24.918935
H	20.418149	19.242377	23.807162
H	27.294062	14.294431	16.783670
H	16.059756	22.149844	25.184876
H	21.447337	23.633710	27.519029
H	14.412951	24.834592	18.615174
H	19.672976	26.540339	21.082174
H	25.351744	12.194548	8.846410
H	26.790291	19.246211	10.648830
H	29.870832	13.138148	12.514911
H	31.157036	20.225497	14.447496

=== [1]**TS6** ===

Cd	19.415000	24.466000	24.406000
Cd	16.370000	24.133000	21.810000
Cd	29.590000	17.101000	12.821000
Cd	27.131000	15.292000	10.287000
O	30.396000	14.953000	13.259000
O	26.325000	17.440000	9.849000
O	28.931000	14.769000	11.743000
O	27.789000	17.624000	11.364000
O	29.974000	19.426000	13.017000
O	26.747000	12.968000	10.091000
O	31.101000	18.203000	14.445000
O	25.620000	14.190000	8.663000
O	19.734000	23.407000	26.461000
O	16.050000	25.192000	19.755000
O	21.353000	24.735000	25.823000
O	14.431000	23.864000	20.393000
O	20.336000	25.881000	22.872000
O	15.448000	22.718000	23.344000
O	17.349000	23.397000	24.245000
O	18.436000	25.202000	21.971000
C	17.598110	22.255921	19.423464
C	17.788942	21.151749	18.609262
C	17.223618	19.922843	18.977781
C	16.407611	19.899805	20.112600
C	16.272483	21.050925	20.875426
C	17.545518	18.682766	18.259780
C	18.764660	18.498025	17.737021
C	19.295490	17.305750	17.075235
C	18.501240	16.239914	16.630552
C	19.122404	15.163303	16.027320
C	20.501259	15.091242	15.844647
C	21.288353	16.148953	16.287466
C	20.675390	17.245929	16.890662
C	22.778940	16.186963	16.127559
C	23.565823	14.932301	15.718453
C	24.057633	14.824473	14.302697
C	25.039759	13.878783	13.999718
C	25.745836	13.991669	12.814463
C	24.492609	15.812586	12.160722
C	23.726591	15.755226	13.319241
C	28.150595	15.585427	15.328059
C	27.106600	15.170658	16.146092
C	25.910802	15.888361	16.155626
C	25.871876	17.072021	15.411431
C	26.947003	17.407419	14.604959
C	24.674497	15.315832	16.790318
C	23.690140	16.351880	17.394609
C	23.039917	16.012042	18.703201
C	22.906058	17.016973	19.655520
C	22.147688	16.830159	20.814799
C	21.550515	15.594540	21.052650
C	21.740214	14.581576	20.120503
C	22.460044	14.763869	18.951769
C	21.923338	18.011561	21.674379
C	20.711596	18.546486	21.848453
C	20.503901	19.866000	22.464863
C	19.637110	20.767440	21.841325
C	19.535859	22.053754	22.341030
C	21.004414	21.600290	24.050304
C	21.198284	20.301759	23.596269
C	29.743686	14.231093	12.508643
C	26.959931	18.159774	10.615350
C	30.761797	19.303555	13.985851
C	25.890141	13.097071	9.185756
C	19.513224	25.858938	21.936430
C	16.297981	22.712114	24.266419
C	20.854720	23.938833	26.638179
C	14.952819	24.615275	19.553971
N	25.511591	14.981125	11.943231
N	16.889214	22.195264	20.557248
N	28.052700	16.659247	14.536835

N	20.196361	22.463892	23.427819
F	18.370483	14.146851	15.590155
F	21.183215	13.386803	20.351547
H	18.017793	23.227947	19.175415
H	18.367297	21.244536	17.696651
H	15.915321	18.983920	20.423322
H	15.668105	21.074892	21.777324
H	16.798004	17.892096	18.255339
H	19.492240	19.299906	17.856689
H	17.421105	16.242159	16.727364
H	20.920500	14.221322	15.349193
H	21.292443	18.071615	17.238638
H	23.020050	17.008360	15.445400
H	23.066460	14.010189	16.036564
H	25.308677	13.109890	14.718532
H	26.542106	13.304289	12.542871
H	24.325299	16.568453	11.397724
H	22.928938	16.475686	13.464715
H	29.083443	15.033397	15.261481
H	24.992483	17.706541	15.397536
H	26.923448	18.283564	13.962509
H	24.938236	14.496045	17.464569
H	24.129408	17.350378	17.477278
H	23.349428	17.992191	19.469693
H	20.957041	15.407558	21.940969
H	22.529249	13.939244	18.248690
H	22.803445	18.540389	22.040036
H	19.845431	18.074386	21.385702
H	19.079445	20.479352	20.955496
H	18.916757	22.799676	21.855513
H	21.505649	21.971590	24.938493
H	21.864328	19.631769	24.129713
H	27.210472	14.260799	16.729593
H	16.114565	22.065998	25.138657
H	21.423159	23.673017	27.546902
H	14.448121	24.780948	18.586331
H	19.737131	26.436680	21.026482
H	25.350414	12.194832	8.847540
H	26.791441	19.246537	10.647854
H	29.867475	13.137749	12.517985
H	31.160599	20.225432	14.444551

=== ^{1}**Int10 (CP1-1’)** ===

Cd	19.415000	24.466000	24.406000
Cd	16.370000	24.133000	21.810000
Cd	29.590000	17.101000	12.821000
Cd	27.131000	15.292000	10.287000
O	30.396000	14.953000	13.259000
O	26.325000	17.440000	9.849000
O	28.931000	14.769000	11.743000
O	27.789000	17.624000	11.364000
O	29.974000	19.426000	13.017000
O	26.747000	12.968000	10.091000
O	31.101000	18.203000	14.445000
O	25.620000	14.190000	8.663000
O	19.734000	23.407000	26.461000
O	16.050000	25.192000	19.755000
O	21.353000	24.735000	25.823000
O	14.431000	23.864000	20.393000
O	20.336000	25.881000	22.872000
O	15.448000	22.718000	23.344000
O	17.349000	23.397000	24.245000
O	18.436000	25.202000	21.971000
C	18.375679	21.680054	20.749531
C	18.704106	20.432291	20.246887
C	17.679496	19.549039	19.878963
C	16.363459	19.965707	20.098392
C	16.120931	21.232131	20.611413
C	17.953980	18.248346	19.252031
C	19.073806	18.020816	18.553905
C	19.490118	16.800190	17.859846
C	18.730177	15.623002	17.786355
C	19.267737	14.533409	17.128542
C	20.541521	14.536688	16.560872
C	21.295775	15.700757	16.637221
C	20.746130	16.821856	17.259751
C	22.724467	15.835130	16.195510
C	23.563582	14.633848	15.715604
C	24.013896	14.582886	14.279019
C	25.047048	13.703801	13.942847
C	25.759658	13.905156	12.773459
C	24.412920	15.672500	12.176657
C	23.638284	15.525740	13.323521
C	28.206761	15.543626	15.308253
C	27.172766	15.056110	16.098523
C	25.923822	15.670547	16.046921
C	25.802494	16.818099	15.256536
C	26.869751	17.224354	14.476763
C	24.722104	15.068727	16.715939
C	23.769571	16.114319	17.339630
C	23.293143	15.900060	18.742134
C	23.161699	17.021740	19.552311
C	22.548687	16.960897	20.804457
C	22.113591	15.724610	21.289456
C	22.295503	14.605479	20.492921
C	22.863105	14.664079	19.226750
C	22.320740	18.240398	21.476855
C	21.324689	18.522601	22.324397
C	21.024970	19.873615	22.809533
C	20.023638	20.054914	23.766192
C	19.668053	21.338840	24.150166
C	21.227159	22.268933	22.731955
C	21.650544	21.023300	22.303401
C	29.740253	14.230749	12.512452
C	26.960686	18.160019	10.613581
C	30.762825	19.303722	13.984460
C	25.891062	13.096809	9.184513
C	19.503673	25.864792	21.938440
C	16.305893	22.692950	24.249093
C	20.872512	23.909281	26.617188
C	14.953630	24.625180	19.555418
N	25.484892	14.916801	11.938308
N	17.111710	22.079934	20.919696
N	28.042143	16.583025	14.483740
N	20.238841	22.424569	23.619339
F	18.544554	13.410046	17.046545
F	21.893776	13.416491	20.956354
H	19.143719	22.396268	21.028853
H	19.747526	20.148747	20.159341
H	15.529100	19.320194	19.843907
H	15.112305	21.605315	20.765861
H	17.177478	17.490317	19.334839
H	19.788951	18.836961	18.464278
H	17.742183	15.538469	18.225094
H	20.903800	13.632355	16.082850
H	21.339194	17.731953	17.319831
H	22.785277	16.658348	15.476654
H	23.149019	13.672805	16.038963
H	25.358897	12.926194	14.634621
H	26.593904	13.271343	12.488016
H	24.206487	16.448076	11.443407
H	22.804180	16.199740	13.485623
H	29.187328	15.077786	15.292174
H	24.867616	17.363946	15.187964
H	26.791591	18.068794	13.797477
H	25.027865	14.271638	17.399619
H	24.182183	17.124938	17.278373
H	23.494871	17.986592	19.177377
H	21.656075	15.614213	22.266564
H	22.932577	13.751814	18.641063
H	22.988530	19.038462	21.158831
H	20.637192	17.738816	22.637665
H	19.516602	19.200226	24.202049
H	18.907912	21.523913	24.902021
H	21.673540	23.184373	22.351347
H	22.438976	20.963709	21.562193
H	27.335404	14.178793	16.717154
H	16.147726	22.006639	25.097557
H	21.467022	23.594638	27.492131
H	14.436605	24.805253	18.597396

H	19.721013	26.457491	21.036314
H	25.352692	12.194246	8.845241
H	26.794852	19.247161	10.643571
H	29.856835	13.136613	12.528733
H	31.164332	20.225325	14.441254

=== ^{1}**Int10** (**CP1-1’** using its X-ray structure) ===

Cd	28.084000	9.147000	12.773000
Cd	25.225000	6.943000	10.478000
Cd	15.920000	16.601000	24.551000
Cd	13.273000	15.579000	21.950000
O	29.689000	10.226000	14.206000
O	23.620000	5.864000	9.044000
O	28.402000	11.476000	12.906000
O	24.907000	4.614000	10.344000
O	27.013000	6.582000	11.939000
O	26.296000	9.508000	11.311000
O	28.767000	7.013000	13.190000
O	24.542000	9.077000	10.060000
O	13.979000	15.282000	24.450000
O	15.214000	16.898000	22.051000
O	12.282000	14.260000	23.553000
O	16.911000	17.920000	22.948000
O	16.189000	15.696000	26.657000
O	13.005000	16.484000	19.844000
O	17.701000	17.213000	26.085000
O	11.493000	14.967000	20.416000
F	20.738868	6.340256	21.495133
F	17.407309	5.615611	17.307317
N	23.832304	6.794600	12.357865
N	14.535021	13.695611	21.398968
N	26.556287	8.935214	14.544882
N	17.039615	14.715366	23.672498
C	24.217452	5.977060	13.345424
H	25.033818	5.301522	13.106890
C	23.641817	6.023672	14.603988
H	24.025552	5.388184	15.396915
C	22.632797	6.955658	14.863346
C	22.156450	7.705817	13.789397
H	21.335998	8.405368	13.901572
C	22.795566	7.608381	12.558762
H	22.504783	8.226766	11.713688
C	22.243879	7.233265	16.289028
H	21.885282	6.320055	16.776224
C	21.334085	8.437062	16.592455
H	21.353140	9.161270	15.771659
C	19.908940	8.198633	17.007022
C	19.289417	6.956019	16.914418
H	19.769100	6.095644	16.459897
C	17.998571	6.811163	17.415495
C	17.314561	7.840046	18.031993
H	16.315760	7.658220	18.412728
C	17.934638	9.094193	18.121961
C	19.204919	9.261359	17.572767
H	19.679008	10.237379	17.636279
C	17.334890	10.248244	18.793591
H	17.844742	11.189352	18.598209
C	16.276332	10.241711	19.614069
H	15.761639	9.308306	19.833775
C	15.719690	11.441526	20.247758
C	16.304538	12.711538	20.134905
H	17.236860	12.863552	19.603321
C	15.680859	13.800733	20.715050
H	16.094450	14.802917	20.637705
C	13.974106	12.489058	21.538218
H	13.043434	12.460456	22.099324
C	14.536022	11.346287	20.987116
H	14.039823	10.388875	21.110260
C	25.420350	9.639377	14.545426
H	25.327146	10.392419	13.767760
C	24.401699	9.405469	15.456305
H	23.484201	9.981374	15.381406
C	24.543319	8.380456	16.396133
C	25.766049	7.710105	16.433942
H	25.941603	6.920671	17.158363
C	26.742406	8.010992	15.492929
H	27.690921	7.481521	15.460028
C	23.377495	7.876520	17.196586
H	23.735314	7.201777	17.979281
C	22.354061	8.928738	17.694272
H	22.689208	9.956935	17.527880
C	21.405730	9.963651	19.749789
H	21.578180	10.931036	19.282467
C	18.370487	12.444288	22.727572
C	21.839738	8.808467	19.100179
C	21.610347	7.570635	19.701642
H	21.916570	6.634319	19.244805
C	20.940120	7.529577	20.916270
C	20.455472	8.664267	21.542640
H	19.936192	8.566921	22.489587
C	20.676394	9.908126	20.941097
C	20.111391	11.152258	21.479767
H	20.628649	12.065424	21.190144
C	18.997128	11.217689	22.221667
H	18.469915	10.295841	22.462424
C	18.848160	13.733783	22.455412
H	19.747138	13.895308	21.870943
C	18.155451	14.831097	22.940936
H	18.489872	15.847778	22.748424
C	16.587766	13.492224	23.962888
H	15.693259	13.453051	24.576629
C	17.212808	12.341499	23.505548
H	16.798623	11.368319	23.750152
C	27.989827	6.216530	12.620637
C	25.306787	9.872459	10.646645
C	29.283586	11.334349	13.792872
C	23.968846	4.760190	9.519520
C	13.019578	14.479324	24.525739
C	16.199483	17.665617	21.963554
C	17.238643	16.361574	26.873953
C	11.996547	15.765548	19.598182
H	23.416665	3.858353	9.202969
H	29.718291	12.248701	14.232773
H	28.179725	5.137230	12.745894
H	25.083674	10.949818	10.569969
H	17.778055	16.168827	27.817768
H	12.841040	13.954824	25.477313
H	16.441373	18.111393	20.986288
H	11.543269	15.852791	18.595510

=== ^{3}**Int10** ===

Cd	19.415000	24.466000	24.406000
Cd	16.370000	24.133000	21.810000
Cd	29.590000	17.101000	12.821000
Cd	27.131000	15.292000	10.287000
O	30.396000	14.953000	13.259000
O	26.325000	17.440000	9.849000
O	28.931000	14.769000	11.743000
O	27.789000	17.624000	11.364000
O	29.974000	19.426000	13.017000
O	26.747000	12.968000	10.091000
O	31.101000	18.203000	14.445000
O	25.620000	14.190000	8.663000
O	19.734000	23.407000	26.461000
O	16.050000	25.192000	19.755000
O	21.353000	24.735000	25.823000
O	14.431000	23.864000	20.393000
O	20.336000	25.881000	22.872000
O	15.448000	22.718000	23.344000
O	17.349000	23.397000	24.245000
O	18.436000	25.202000	21.971000
C	17.923966	22.186388	19.672779
C	18.136763	21.077606	18.868129
C	17.501901	19.867315	19.182410
C	16.671501	19.857014	20.307206
C	16.506922	21.013405	21.054420
C	17.758997	18.608677	18.472785
C	18.475338	18.469785	17.348508
C	19.014486	17.179451	16.898959
C	18.398083	15.955067	17.182777

C	19.110813	14.792855	16.955144
C	20.427543	14.790622	16.507767
C	21.036708	16.005638	16.206507
C	20.295397	17.181135	16.345495
C	22.502831	16.146070	15.913068
C	23.379990	14.930470	15.568645
C	23.898728	14.795042	14.165352
C	24.917106	13.869187	13.925172
C	25.679073	13.983742	12.776287
C	24.406568	15.753176	12.026702
C	23.585188	15.692363	13.146326
C	28.008577	15.672130	15.284086
C	26.930894	15.280553	16.069643
C	25.722024	15.970428	15.976557
C	25.691139	17.096939	15.149538
C	26.804365	17.417023	14.389150
C	24.472840	15.427940	16.613351
C	23.463920	16.478461	17.125944
C	22.858056	16.328108	18.493341
C	22.017431	17.355700	18.892991
C	21.303733	17.325713	20.108417
C	21.530835	16.237438	20.986928
C	22.378451	15.232094	20.576316
C	23.031401	15.232225	19.341917
C	20.365881	18.353161	20.350551
C	19.684912	18.611059	21.621830
C	19.837804	19.856542	22.282799
C	19.271664	20.095047	23.556261
C	19.395187	21.341647	24.131484
C	20.557699	22.160656	22.311262
C	20.527558	20.933650	21.676626
C	29.741374	14.230922	12.511137
C	26.963865	18.160291	10.611229
C	30.762586	19.303769	13.984632
C	25.889336	13.097159	9.186923
C	19.501949	25.868514	21.941259
C	16.300037	22.710228	24.262668
C	20.874677	23.908249	26.615756
C	14.950685	24.621716	19.557006
N	25.462422	14.954113	11.879409
N	17.135915	22.156275	20.754442
N	27.932865	16.700935	14.433048
N	19.996096	22.371924	23.506225
F	18.525516	13.617880	17.212683
F	22.579455	14.185553	21.387656
H	18.394877	23.141627	19.455845
H	18.805398	21.162153	18.018172
H	16.150337	18.950099	20.597768
H	15.850565	21.045918	21.918492
H	17.435984	17.711826	19.000062
H	18.836876	19.349509	16.817770
H	17.388716	15.894904	17.574906
H	20.936594	13.838225	16.414450
H	20.769938	18.134023	16.117985
H	22.631624	16.935128	15.166550
H	22.957739	13.986721	15.926115
H	25.173254	13.123384	14.672516
H	26.508070	13.316613	12.557823
H	24.255935	16.491479	11.243217
H	22.761608	16.391631	13.235056
H	28.954588	15.137352	15.298270
H	24.791173	17.694384	15.041512
H	26.798980	18.251448	13.693796
H	24.745626	14.657968	17.338675
H	23.857899	17.496834	17.052624
H	21.884288	18.216031	18.243379
H	21.036238	16.164430	21.949185
H	23.659043	14.384156	19.093952
H	20.166695	19.036229	19.529029
H	19.008142	17.873695	22.049414
H	18.755930	19.298921	24.083952
H	19.004496	21.562085	25.120495
H	21.042227	23.019872	21.854354
H	21.001654	20.815015	20.709610
H	27.024044	14.411647	16.714148
H	16.122328	22.051034	25.128280
H	21.468841	23.592530	27.490140
H	14.436472	24.800482	18.596783
H	19.717056	26.466686	21.041740
H	25.347026	12.195535	8.850954
H	26.801283	19.248261	10.637738
H	29.862924	13.137437	12.523364
H	31.163970	20.225112	14.441928

=== ^{3}**TS7** ===

Cd	19.415000	24.466000	24.406000
Cd	16.370000	24.133000	21.810000
Cd	29.590000	17.101000	12.821000
Cd	27.131000	15.292000	10.287000
O	30.396000	14.953000	13.259000
O	26.325000	17.440000	9.849000
O	28.931000	14.769000	11.743000
O	27.789000	17.624000	11.364000
O	29.974000	19.426000	13.017000
O	26.747000	12.968000	10.091000
O	31.101000	18.203000	14.445000
O	25.620000	14.190000	8.663000
O	19.734000	23.407000	26.461000
O	16.050000	25.192000	19.755000
O	21.353000	24.735000	25.823000
O	14.431000	23.864000	20.393000
O	20.336000	25.881000	22.872000
O	15.448000	22.718000	23.344000
O	17.349000	23.397000	24.245000
O	18.436000	25.202000	21.971000
C	18.261637	21.970431	20.204812
C	18.715018	20.749142	19.744357
C	18.002166	19.576435	20.071604
C	16.809707	19.743114	20.801660
C	16.416686	21.005618	21.195684
C	18.441669	18.262485	19.711669
C	19.610828	18.007927	18.981260
C	19.910232	16.742840	18.327864
C	19.319543	15.512951	18.668704
C	19.807777	14.362105	18.082064
C	20.926318	14.347390	17.248091
C	21.519885	15.559768	16.912628
C	20.951639	16.739031	17.395459
C	22.907445	15.711208	16.364644
C	23.761645	14.527276	15.865320
C	24.180221	14.503448	14.415208
C	25.235008	13.667014	14.038846
C	25.897260	13.896433	12.844215
C	24.464566	15.609468	12.300096
C	23.737072	15.431376	13.473472
C	28.370056	15.555665	15.375294
C	27.374646	15.032492	16.192000
C	26.103572	15.597758	16.161736
C	25.918736	16.736681	15.370346
C	26.946905	17.175667	14.560455
C	24.931507	14.963961	16.852769
C	23.999089	16.009649	17.489783
C	23.478116	15.872151	18.881605
C	22.898652	17.027483	19.389234
C	21.981696	17.008303	20.448562
C	21.777292	15.789378	21.125460
C	22.457197	14.670397	20.683256
C	23.268740	14.665497	19.547450
C	21.207984	18.205885	20.634729
C	20.446540	18.501318	21.820089
C	20.354266	19.803532	22.385323
C	19.537283	20.035230	23.516049
C	19.431999	21.307162	24.041210
C	20.863246	22.168736	22.454848
C	21.053935	20.926176	21.879170
C	29.740533	14.230852	12.512625
C	26.959723	18.159867	10.614319
C	30.760262	19.303733	13.986761
C	25.892368	13.096391	9.183788
C	19.502242	25.866875	21.940069

C	16.301318	22.704891	24.258123
C	20.871953	23.910594	26.617821
C	14.953864	24.618918	19.555458
N	25.556060	14.896780	12.019468
N	17.146264	22.103436	20.929659
N	28.140843	16.578136	14.545848
N	20.054586	22.369927	23.500997
F	19.233717	13.192332	18.386392
F	22.273050	13.512939	21.329016
H	18.807450	22.888146	20.000521
H	19.633012	20.716565	19.168796
H	16.200547	18.882444	21.058958
H	15.505647	21.168946	21.760938
H	17.823466	17.430702	20.039762
H	20.071144	18.860879	18.490624
H	18.504889	15.431989	19.379521
H	21.309067	13.390613	16.909087
H	21.412419	17.687032	17.126230
H	22.919402	16.540366	15.651706
H	23.365209	13.556310	16.179270
H	25.600986	12.899706	14.715296
H	26.745009	13.294635	12.530864
H	24.198274	16.376741	11.577353
H	22.882605	16.071416	13.664387
H	29.367342	15.129046	15.336543
H	24.962708	17.245271	15.317617
H	26.818217	18.006885	13.872101
H	25.266191	14.164135	17.519370
H	24.402410	17.020140	17.387396
H	23.069459	17.969035	18.873081
H	21.103525	15.696486	21.969924
H	23.680120	13.722899	19.200278
H	21.627506	19.069160	20.126223
H	19.941881	17.691357	22.339830
H	18.997139	19.213777	23.976114
H	18.837787	21.514266	24.925083
H	21.371434	23.050029	22.071234
H	21.745114	20.839544	21.049811
H	27.584869	14.166215	16.811609
H	16.130693	22.035679	25.117887
H	21.463915	23.600197	27.495813
H	14.442785	24.792089	18.592482
H	19.718512	26.462999	21.039578
H	25.356539	12.193450	8.841738
H	26.792882	19.246727	10.645291
H	29.856690	13.136556	12.529056
H	31.156330	20.225704	14.447655

=== ^{3}**Int11** ===

Cd	19.415000	24.466000	24.406000
Cd	16.370000	24.133000	21.810000
Cd	29.590000	17.101000	12.821000
Cd	27.131000	15.292000	10.287000
O	30.396000	14.953000	13.259000
O	26.325000	17.440000	9.849000
O	28.931000	14.769000	11.743000
O	27.789000	17.624000	11.364000
O	29.974000	19.426000	13.017000
O	26.747000	12.968000	10.091000
O	31.101000	18.203000	14.445000
O	25.620000	14.190000	8.663000
O	19.734000	23.407000	26.461000
O	16.050000	25.192000	19.755000
O	21.353000	24.735000	25.823000
O	14.431000	23.864000	20.393000
O	20.336000	25.881000	22.872000
O	15.448000	22.718000	23.344000
O	17.349000	23.397000	24.245000
O	18.436000	25.202000	21.971000
C	18.194323	21.975523	20.172855
C	18.593637	20.759731	19.658173
C	17.777195	19.621357	19.842149
C	16.524894	19.832819	20.460766
C	16.201577	21.089189	20.933412
C	18.215698	18.317231	19.487954
C	19.605176	18.078858	19.024355
C	19.827702	16.789494	18.273523
C	19.263858	15.562725	18.627930
C	19.760055	14.412263	18.030705
C	20.872622	14.412673	17.196268
C	21.438735	15.636788	16.848414
C	20.825273	16.804831	17.303307
C	22.832791	15.826780	16.324101
C	23.727283	14.655552	15.869824
C	24.168549	14.602578	14.426942
C	25.228209	13.758322	14.081664
C	25.897451	13.953441	12.884520
C	24.467794	15.649167	12.282982
C	23.733243	15.504559	13.456568
C	28.323601	15.628517	15.397285
C	27.316715	15.154266	16.229593
C	26.057136	15.744700	16.178414
C	25.896639	16.861156	15.351216
C	26.937314	17.255967	14.533820
C	24.869179	15.141176	16.868346
C	23.911804	16.196032	17.452018
C	23.391804	16.111364	18.850534
C	22.718813	17.254015	19.278987
C	21.740501	17.203882	20.264269
C	21.607426	16.035941	21.015977
C	22.411703	14.950519	20.693043
C	23.254390	14.937540	19.587428
C	20.727308	18.325366	20.242970
C	20.121311	18.619553	21.569082
C	20.234319	19.861535	22.254738
C	19.518233	20.066591	23.456736
C	19.495356	21.316341	24.044666
C	20.876935	22.187669	22.423628
C	20.983067	20.967333	21.782828
C	29.739599	14.230799	12.513625
C	26.959985	18.159815	10.614202
C	30.759346	19.303748	13.987462
C	25.892651	13.096323	9.183955
C	19.502355	25.868001	21.940589
C	16.305058	22.697939	24.253539
C	20.873508	23.908640	26.616183
C	14.956436	24.616079	19.553355
N	25.560183	14.928107	12.028476
N	17.040401	22.134512	20.826225
N	28.117941	16.631630	14.538954
N	20.121680	22.379008	23.512818
F	19.204457	13.237214	18.349646
F	22.294824	13.832342	21.418365
H	18.819718	22.860642	20.088359
H	19.555164	20.693045	19.163152
H	15.833229	19.007583	20.596196
H	15.262517	21.288569	21.439554
H	17.532277	17.484592	19.622212
H	19.855328	18.877431	18.317051
H	18.485485	15.465619	19.377735
H	21.281141	13.460746	16.874027
H	21.265081	17.759558	17.023319
H	22.831034	16.637990	15.590296
H	23.348917	13.682752	16.199497
H	25.589199	13.009474	14.780992
H	26.748655	13.344610	12.594671
H	24.206236	16.395532	11.536948
H	22.876753	16.148854	13.621556
H	29.311070	15.177854	15.370760
H	24.948469	17.382241	15.275750
H	26.829646	18.070207	13.822274
H	25.189263	14.365694	17.569762
H	24.292534	17.209491	17.302047
H	22.827034	18.168219	18.699512
H	20.878142	15.928505	21.811564
H	23.741472	14.008124	19.309537
H	21.263513	19.215420	19.907018
H	19.512922	17.849859	22.038057
H	18.960088	19.250357	23.904522
H	18.954593	21.506099	24.966909

H	21.402737	23.063965	22.052529
H	21.616354	20.895922	20.906056
H	27.506645	14.302727	16.875748
H	16.140938	22.020128	25.107548
H	21.467459	23.594036	27.491289
H	14.449455	24.782251	18.586969
H	19.717074	26.465755	21.040906
H	25.357403	12.193275	8.841170
H	26.793138	19.246762	10.645022
H	29.855120	13.136484	12.530835
H	31.154106	20.225720	14.449559

=== ^{1}**Int12** (**CP1-2α**) ===

Cd	19.415000	24.466000	24.406000
Cd	16.370000	24.133000	21.810000
Cd	29.590000	17.101000	12.821000
Cd	27.131000	15.292000	10.287000
O	30.396000	14.953000	13.259000
O	26.325000	17.440000	9.849000
O	28.931000	14.769000	11.743000
O	27.789000	17.624000	11.364000
O	29.974000	19.426000	13.017000
O	26.747000	12.968000	10.091000
O	31.101000	18.203000	14.445000
O	25.620000	14.190000	8.663000
O	19.734000	23.407000	26.461000
O	16.050000	25.192000	19.755000
O	21.353000	24.735000	25.823000
O	14.431000	23.864000	20.393000
O	20.336000	25.881000	22.872000
O	15.448000	22.718000	23.344000
O	17.349000	23.397000	24.245000
O	18.436000	25.202000	21.971000
C	18.367344	22.087028	20.333928
C	19.035308	20.893476	20.124825
C	18.458957	19.699394	20.567410
C	17.160878	19.758086	21.070721
C	16.543527	20.992773	21.217966
C	19.268599	18.442169	20.662697
C	20.312664	18.215087	19.554742
C	20.265899	17.019142	18.645656
C	19.509613	15.866037	18.858737
C	19.809330	14.731226	18.115540
C	20.905485	14.650417	17.264398
C	21.661066	15.801556	17.054070
C	21.251895	16.988502	17.661816
C	23.059147	15.841022	16.507854
C	23.801649	14.614171	15.930786
C	24.188922	14.603763	14.469518
C	25.174978	13.705626	14.049487
C	25.824369	13.911189	12.842583
C	24.508970	15.733873	12.369871
C	23.799067	15.585768	13.559648
C	28.448210	15.487286	15.370991
C	27.456649	14.942176	16.177063
C	26.201267	15.538929	16.202762
C	26.031534	16.727541	15.483934
C	27.051842	17.185138	14.677368
C	25.027274	14.913823	16.896697
C	24.193115	15.977246	17.630916
C	23.710979	15.794674	19.032804
C	23.267173	16.969765	19.629915
C	22.306316	16.970179	20.635924
C	21.971334	15.757789	21.235829
C	22.572156	14.598284	20.756075
C	23.381437	14.576744	19.626293
C	21.474901	18.214800	20.661567
C	20.433197	18.487384	21.756586
C	20.519114	19.788320	22.516326
C	19.593546	20.023975	23.537173
C	19.466526	21.295247	24.073374
C	21.144953	22.103214	22.735613
C	21.357804	20.849192	22.171094
C	29.741739	14.230777	12.511100
C	26.958032	18.159524	10.616426
C	30.759197	19.303436	13.988226
C	25.892425	13.096575	9.182916
C	19.501372	25.870152	21.941653
C	16.294641	22.712124	24.262730
C	20.872984	23.907517	26.615960
C	14.955058	24.616567	19.554253
N	25.536122	14.947618	12.041559
N	17.163046	22.136124	20.904912
N	28.226665	16.557113	14.601304
N	20.188627	22.335908	23.634291
F	19.064630	13.634207	18.300951
F	22.265688	13.435245	21.342893
H	18.814834	23.044011	20.077222
H	20.031870	20.922338	19.699573
H	16.648583	18.856926	21.393604
H	15.550793	21.092156	21.643896
H	18.612826	17.584283	20.832839
H	20.421755	19.101351	18.924652
H	18.717595	15.806354	19.597225
H	21.154321	13.692868	16.818793
H	21.843650	17.886292	17.493007
H	23.145655	16.702783	15.839024
H	23.332627	13.668948	16.224527
H	25.500475	12.898641	14.700198
H	26.616197	13.253487	12.495681
H	24.281352	16.533745	11.669717
H	23.001379	16.285109	13.787379
H	29.433973	15.042456	15.289549
H	25.092030	17.266579	15.486282
H	26.930993	18.060953	14.044660
H	25.342246	14.057062	17.499010
H	24.670698	16.958771	17.579351
H	23.517542	17.916425	19.156486
H	21.228953	15.671168	22.022582
H	23.688825	13.619433	19.216285
H	22.136722	19.064825	20.486365
H	20.321083	17.663375	22.467428
H	18.939695	19.228618	23.883197
H	18.758074	21.516787	24.864430
H	21.740503	22.962229	22.437674
H	22.139316	20.749307	21.427525
H	27.656976	14.039172	16.745215
H	16.114493	22.058799	25.132519
H	21.468610	23.590879	27.489264
H	14.446883	24.785569	18.589157
H	19.713316	26.472291	21.044482
H	25.358709	12.193026	8.839451
H	26.788138	19.245826	10.650424
H	29.858594	13.136603	12.525952
H	31.151937	20.225967	14.451158

=== ^{1}**Int12** (**CP1-2α**, using its X-ray structure) ===

Cd	21.692000	15.263000	22.797000
Cd	24.663000	17.464000	24.931000
Cd	13.030000	24.911000	37.170000
Cd	10.067000	24.179000	34.422000
O	20.994000	17.386000	22.361000
O	25.361000	15.341000	25.368000
O	23.574000	14.930000	24.153000
O	22.781000	17.797000	23.575000
O	21.445000	12.926000	22.697000
O	24.910000	19.801000	25.031000
O	20.156000	14.093000	21.346000
O	26.199000	18.634000	26.382000
O	10.966000	23.835000	37.042000
O	12.131000	25.256000	34.550000
O	13.877000	26.163000	35.500000
O	9.221000	22.927000	36.092000
O	13.272000	23.982000	39.283000
O	9.826000	25.109000	32.310000
O	14.884000	25.351000	38.636000
O	8.214000	23.739000	32.956000
F	20.069559	18.383670	34.250159
F	16.738505	14.608434	33.452169
N	20.279626	15.270132	24.695285

N	23.271411	17.251358	26.807233
N	14.075273	23.001183	36.270863
N	11.016221	22.167476	33.632808
C	20.265608	14.206690	25.506983
H	20.725698	13.305176	25.108412
C	19.724139	14.260804	26.786017
H	19.773499	13.385774	27.427181
C	19.184294	15.458820	27.258992
C	19.122159	16.528661	26.363573
H	18.697071	17.484867	26.653561
C	19.685871	16.397101	25.103243
H	19.688666	17.216300	24.390232
C	18.899763	15.636697	28.723793
H	18.828425	14.655254	29.200265
C	17.777085	16.624936	29.126952
H	17.187369	16.974325	28.273633
C	16.849864	16.290769	30.263126
C	15.783011	17.172591	30.452036
H	15.529938	17.859006	29.647488
C	15.204802	17.370746	31.706738
C	15.516496	16.472247	32.728469
H	15.094736	16.546823	33.725610
C	16.450690	15.476858	32.475840
C	17.175046	15.408920	31.292454
H	17.993064	14.699240	31.223558
C	14.604726	18.728704	31.959792
H	14.132650	19.099828	31.044330
C	13.758852	18.936995	33.235338
H	13.375689	18.020983	33.694752
C	22.495614	18.277955	27.160025
H	22.700314	19.221863	26.659170
C	23.105663	16.074644	27.422138
H	23.805705	15.293649	27.146183
C	22.094691	15.857887	28.342290
H	21.963503	14.872157	28.779025
C	21.464990	18.145242	28.082129
H	20.855032	19.009454	28.318697
C	21.208497	16.893777	28.641829
C	19.960334	16.590351	29.419180
H	20.210085	16.156643	30.392714
C	18.895993	17.688035	29.556083
H	18.968754	18.424317	28.749313
C	18.753105	18.380658	30.886143
C	19.606074	18.138827	31.965050
H	20.542473	17.601332	31.862943
C	19.233676	18.586567	33.224360
C	17.990355	19.148023	33.476572
H	17.703295	19.359290	34.501911
C	17.144690	19.399256	32.396204
C	15.688948	19.733076	32.567431
H	15.474385	20.730320	32.171337
C	15.038123	19.542480	33.948926
H	15.539794	18.734294	34.492798
C	14.838007	20.735507	34.840899
C	15.339259	21.994627	34.514861
H	16.015853	22.133015	33.678113
C	14.929301	23.100031	35.250070
H	15.261165	24.105485	35.005008
C	13.664635	21.787685	36.656353
H	13.007817	21.765190	37.522108
C	14.028323	20.635428	35.976173
H	13.639360	19.673458	36.297130
C	11.944869	22.207690	32.673142
H	12.037784	23.152305	32.142788
C	12.775367	21.130742	32.396639
H	13.535258	21.239594	31.630913
C	12.668507	19.968808	33.166956
C	11.605996	19.897870	34.068216
H	11.442158	19.002473	34.660551
C	10.808368	21.013883	34.276584
H	10.005732	21.020410	35.008484
C	20.528286	13.012200	21.841490
C	25.772775	19.717304	25.943427
C	21.801755	18.175298	22.904192
C	24.576859	14.552752	24.789741
C	13.130990	25.997954	34.513171
C	9.975879	23.079500	37.074173
C	14.385411	24.547848	39.451424
C	8.755946	24.472055	32.104906
C	17.599999	19.131136	31.106443
H	16.958746	19.369354	30.263676
H	8.285178	24.571159	31.111627
H	9.758387	22.519267	37.997639
H	14.947366	24.311293	40.371375
H	13.380681	26.517630	33.574260
H	20.029372	12.082097	21.519198
H	24.781014	13.472407	24.874133
H	26.158873	20.657134	26.374628
H	21.619716	19.256429	22.787488

12. Supplementary references

1. Li, W. X. et al. Post-synthetic modification of a two-dimensional metal-organic framework *via* photodimerization enables highly selective luminescent sensing of aluminum(III). *Inorg. Chem.* **57**, 13453-13460 (2018).

2. Amoroso, A. J., Thompson, A., Maher, J. P., Mccleverty, J. A. & Ward, M. D. Di-, tri-, and tetranucleating pyridyl ligands which facilitate multicenter magnetic exchange between paramagnetic molybdenum centers. *Inorg. Chem.* **34**, 4828-4835 (1995).

3. Sheldrick, G. M. Crystal structure refinement with SHELXL. *Acta Crystallogr. Sect. C: Struct. Chem.* **71**, 3-8 (2015).

4. Lautfy, R. O. & de Mayo, P. Primary bond formation in the addition of cyclopentenone to chloroethylenes. *Can. J. Chem.* **50**, 3465-3471 (1972).

5. Brimioulle, R., Guo, H. & Bach, T. Enantioselective intramolecular [2+2] photocycloaddition reactions of 4-substituted coumarins catalyzed by a chiral Lewis acid. *Chem. Eur. J.* **18**, 7552-7560 (2012).

6. Brimioulle, R., Bauer, A. & Bach, T. Enantioselective Lewis acid catalysis in intramolecular [2+2] photocycloaddition reactions: a mechanistic comparison between representative coumarin and enone substrates. *J. Am. Chem. Soc.* **137**, 5170-5176 (2015).

7. Wang, H. J., Cao, X. Y., Chen, X. B., Fang, W. H. & Dolg, M. Regulatory mechanism of the enantioselective intramolecular enone [2+2] photocycloaddition reaction mediated by a chiral Lewis acid catalyst containing heavy atoms. *Angew. Chem., Int. Ed.* **54**, 14295-14298 (2015).

8. Wang, H. J., Fang, F. H. & Chen, X. B. Mechanism of the enantioselective intramolecular [2+2] photocycloaddition reaction of coumarin catalyzed by a chiral Lewis acid: comparison with enone substrates. *J. Org. Chem.* **81**, 7093-7101 (2016).
